# Translating Patient–Professional Divergence into Care-Improvement Priorities in Pelvic Floor Rehabilitation: A Delphi-Dialogue Study

**DOI:** 10.3390/healthcare14162520

**Published:** 2026-08-12

**Authors:** Lourdes Gil-Fraguas, Soraya Hijazi-Vega, Michelle Catta-Preta, Alex Trejo-Omeñaca, Jan Ferrer-Picó, Isabel Montes-Posada, Jesús Vara-Paniagua, Carolina de Miguel-Benadiba, Josep Maria Monguet-Fierro, Helena Bascuñana-Ambrós

**Affiliations:** 1 Spanish Society of Physical Medicine and Rehabilitation (SERMEF), 28016 Madrid, Spain; lgilf@sescam.jccm.es (L.G.-F.); soraya.hijazy@uclm.es (S.H.-V.); imonpos@gobiernodecanarias.org (I.M.-P.); jesusmaria.vara@salud.madrid.org (J.V.-P.); carolina.demiguel@fundacionhospitalarias.org (C.d.M.-B.); josep@innex.io (J.M.M.-F.); 2 Physical Medicine and Rehabilitation Department, Hospital Universitario de Guadalajara, 19002 Guadalajara, Spain; 3 Physical Medicine and Rehabilitation Department, Complejo Hospitalario Universitario de Albacete, 02006 Albacete, Spain; 4 Innex Labs, 08800 Vilanova i la Geltrú, Spain; michelle@innex.io (M.C.-P.); alex@innex.io (A.T.-O.); 5 Institut pel Futur, 17190 Salt, Spain; jan.ferrer.pico@upc.edu; 6 Escuela Politécnica Superior de Ingeniería de Vilanova i la Geltrú, Universitat Politècnica de Catalunya, 08800 Vilanova i la Geltrú, Spain; 7 Hospital Universitario de Gran Canaria Doctor Negrín, 35010 Las Palmas de Gran Canaria, Spain; 8 Hospital Universitario 12 de Octubre, 28041 Madrid, Spain; 9 Fundación Hospitalarias Madrid, Hospital General Universitario, 28007 Madrid, Spain; 10 Physical Medicine and Rehabilitation Department, Hospital Santa Creu i Sant Pau, Campus Salut Barcelona, 08041 Barcelona, Spain; 11 Department of Psychology and Speech and Language Therapy, Universidad Autónoma de Barcelona, 08193 Barcelona, Spain

**Keywords:** pelvic floor disorders, rehabilitation, patient-professional alignment, Real-Time Delphi, adherence, care delivery, value-based health care

## Abstract

**Highlights:**

**What are the main findings?**

- Patient–professional agreement in pelvic floor rehabilitation was domain-specific rather than uniform. The greatest divergences were identified in digital competence, the timing of patient education, bladder and bowel diary use, and adherence to prescribed rehabilitation programmes, whereas hybrid rehabilitation models, educational resources, and overall follow-up were characterized by substantial stakeholder alignment.
- These findings suggest that differences between patients and rehabilitation professionals may be more closely related to how rehabilitation is delivered and experienced than to the clinical effectiveness of the interventions themselves. Organizational and communication factors therefore warrant further investigation as potential contributors to perceived care quality.

**What are the implications of the main findings?**

- The identified patient–professional divergences highlight areas that may warrant further implementation research, particularly in relation to adherence, self-management, patient education, and digital support.
- The Delphi-Dialogue Patients–Professionals (DDPP) framework offers an exploratory, implementation-oriented approach for integrating stakeholder perspectives with perceived implementation feasibility. It may help inform the identification of candidate areas for future patient-centred service-development and implementation research.

**Abstract:**

**Background/Objectives**: Pelvic floor dysfunctions (PFDs) are highly prevalent chronic conditions associated with substantial functional and psychosocial burden. Although multidisciplinary pelvic floor rehabilitation is recommended as first-line management, its effectiveness depends not only on intervention efficacy but also on care delivery, communication, patient engagement, and long-term adherence. This study applied the Delphi-Dialogue Patients–Professionals (DDPP) framework to quantify patient–professional alignment and translate identified patient–professional perceptual differences into candidate service-improvement strategies. **Methods**: A two-phase observational study incorporating parallel stakeholder surveys and a modified Real-Time Delphi feasibility-assessment process was conducted under the auspices of the Spanish Society of Physical Medicine and Rehabilitation (SERMEF). In Phase 1, parallel online questionnaires were completed by 225 patients with PFD and 164 rehabilitation professionals. Shared items were rated on a six-point Likert scale, and patient–professional divergence was quantified using an operational composite index integrating standardized differences in mean ratings and high-agreement responses. In Phase 2, the implementation feasibility of 10 improvement recommendations was evaluated by a Delphi panel comprising 35 PM&R physicians and two representatives of the Spanish Incontinence Association (ASIA). **Results**: Patient–professional agreement was domain-specific. The greatest divergences concerned digital competence, timing of patient education, bladder and bowel diary use, and adherence to prescribed rehabilitation programmes. Professionals attributed greater value to bladder and bowel diaries and selected group-based interventions, whereas patients reported higher digital competence, more timely information delivery, and better adherence. Comparatively similar response patterns were observed regarding hybrid rehabilitation models, educational resources, app-supported follow-up, and overall satisfaction with rehabilitation care. The Delphi process identified structured home exercise programmes, adherence-monitoring strategies, and improved visibility of rehabilitation pathways as the recommendations with the highest perceived implementation feasibility. **Conclusions**: The findings suggest that important challenges in pelvic floor rehabilitation may arise from implementation-related factors in addition to the effectiveness of available interventions. The DDPP framework provides a structured, stakeholder-informed methodology for translating patient–professional divergence into candidate organizational recommendations with comparatively higher perceived implementation feasibility.

## 1. Introduction

Pelvic floor dysfunctions (PFDs) comprise a heterogeneous spectrum of disorders—including urinary incontinence, pelvic organ prolapse, anorectal dysfunction, and chronic pelvic pain—that collectively represent a major and growing public health concern. Owing to their high prevalence, substantial impact on quality of life, and considerable functional, psychological, and socioeconomic burden, PFDs have emerged as a priority within contemporary rehabilitation and chronic disease management frameworks. Epidemiological evidence indicates that up to one-third of women will experience at least one pelvic floor disorder during their lifetime, with prevalence increasing with age, parity, obesity, and other comorbidities. Although considerably less common, PFDs also affect men and individuals with neurological disorders, in whom they contribute substantially to disability and reduced health-related quality of life [1,2,3].

Despite their high prevalence and clinical relevance, PFDs remain consistently underdiagnosed and undertreated. Delayed healthcare seeking is frequently driven by symptom normalization, embarrassment, social stigma, and limited awareness among both patients and healthcare professionals. Furthermore, marked variability in referral pathways and access to specialized multidisciplinary services contributes to significant inequities in care delivery [3]. Consequently, international clinical practice guidelines—including those issued by the International Continence Society (ICS), the European Association of Urology (EAU), and other scientific organizations—recommend pelvic floor muscle training (PFMT) and comprehensive multidisciplinary rehabilitation as first-line treatment for most pelvic floor disorders [4,5,6]. These interventions, integrating supervised exercise, behavioural modification, biofeedback, lifestyle counselling, and patient education, have consistently demonstrated high efficacy in controlled clinical trials.

However, the effectiveness of pelvic floor rehabilitation in routine clinical practice extends beyond the intrinsic efficacy of individual therapeutic interventions. Long-term outcomes depend largely on the quality of healthcare delivery, patient understanding of the rehabilitation process, sustained adherence to prescribed programmes, and continuity of multidisciplinary follow-up. In this regard, pelvic floor rehabilitation constitutes a paradigmatic model of chronic rehabilitation, where successful outcomes rely not only on evidence-based interventions but also on sustained patient engagement, behavioural change, and coordinated longitudinal care. Consequently, deficiencies in access, communication, follow-up organization, and adherence support may substantially reduce real-world treatment effectiveness despite the availability of effective therapeutic strategies.

Against this background, patient-centred rehabilitation has become a fundamental principle of modern rehabilitation medicine. Growing evidence demonstrates that shared decision-making, individualized goal setting, and active patient participation improve treatment adherence, patient satisfaction, self-management, and functional outcomes across a wide range of rehabilitation settings [7,8,9]. These principles are particularly relevant in pelvic floor rehabilitation, where symptom perception, quality of life, and patient-reported outcome measures constitute central indicators of therapeutic success. Aligning rehabilitation strategies with patients’ expectations, preferences, and individual capabilities is therefore essential for optimizing clinical outcomes.

Nevertheless, a substantial implementation gap persists between evidence-based recommendations and everyday clinical practice. Organizational barriers—including heterogeneous referral pathways, unequal availability of specialized pelvic floor rehabilitation units, fragmentation between healthcare levels, and variability in multidisciplinary coordination—continue to limit optimal service delivery [10,11,12]. At the same time, discrepancies between patients’ and professionals’ perceptions regarding information needs, timing of education, therapeutic expectations, adherence barriers, and preferred follow-up models may further compromise rehabilitation outcomes. Comparable misalignments have been identified in other rehabilitation fields as important determinants of reduced patient engagement, poor adherence, and discontinuity of care [13].

These discrepancies can be understood as a prescription–experience gap, whereby interventions supported by robust scientific evidence fail to achieve their full clinical potential because patients experience, interpret, and implement rehabilitation differently from how healthcare professionals prescribe it. This phenomenon is especially relevant in pelvic floor rehabilitation, where therapeutic success depends heavily on self-management, correct performance of pelvic floor exercises, sustained behavioural adaptation, and long-term patient commitment.

The rapid expansion of digital health technologies adds further complexity to this scenario. Telemedicine, mobile health applications, wearable technologies, and hybrid models integrating face-to-face and remote follow-up have the potential to improve accessibility, continuity of care, and healthcare efficiency [14,15]. Nevertheless, their effectiveness depends not only on technological availability but also on their structured integration into clinical pathways, digital health literacy, usability, patient acceptance, and perceived clinical value. Within pelvic floor rehabilitation—where supervision, individualized feedback, and adherence monitoring remain essential—these factors become critical determinants of both safety and treatment effectiveness.

Collectively, these considerations reveal an important unmet need: understanding how patients and rehabilitation professionals perceive the rehabilitation process, identifying where their perspectives converge or diverge, and determining which organizational and clinical aspects should be prioritized to improve care delivery. To date, most published studies have focused primarily on treatment efficacy or clinician-centred models of care, while comparatively little attention has been devoted to systematically integrating patients’ experiences into the design, evaluation, and optimization of rehabilitation services.

To address this gap, the Delphi-Dialogue Patients–Professionals (DDPP) methodology offers a structured participatory framework capable of integrating experiential and clinical knowledge. By combining parallel assessments of patient and professional perspectives with a structured feasibility-assessment process, the DDPP approach enables the systematic identification of areas of alignment and divergence while supporting the development and prioritization of candidate implementation-oriented recommendations.

Previous applications of this methodology in rehabilitation have demonstrated its ability to support patient-centred service innovation through the integration of stakeholder perspectives [13].

Accordingly, the present study applies the DDPP framework to pelvic floor rehabilitation within Physical Medicine and Rehabilitation (PM&R) services.

The objectives were to compare patient and professional perspectives regarding pelvic floor rehabilitation, identify areas of perceived divergence across key domains—including access to care, communication, adherence, and follow-up—and generate a set of candidate recommendations whose perceived implementation feasibility was subsequently assessed through a modified Real-Time Delphi process, with the aim of informing the future development of more coordinated, accessible, and patient-centred rehabilitation pathways.

## 2. Objectives

### 2.1. General Objective

This study aimed to identify and quantify key areas of convergence and divergence between patients with pelvic floor dysfunctions (PFDs) and Physical Medicine and Rehabilitation (PM&R) professionals regarding selected components of rehabilitation care in Spain, and to translate these findings into candidate implementation-oriented recommendations through a modified Real-Time Delphi feasibility-assessment process conducted under the auspices of the Spanish Society of Physical Medicine and Rehabilitation (SERMEF).

### 2.2. Specific Objectives

1. To compare patient and professional perceptions of PM&R care pathways for PFDs, including access to specialized rehabilitation services, multidisciplinary team involvement, adequacy and timing of clinical information, and organization of follow-up care.
2. To examine the degree of alignment and divergence between patients and professionals regarding communication strategies, support for home-based rehabilitation, group interventions, and digital care models including telemedicine, mobile applications, and hybrid follow-up pathways.
3. To quantify patient–professional disagreement using the Delphi-Dialogue Patients–Professionals (DDPP) concordance framework, integrating differences in central tendency and levels of high agreement to identify perceptual discrepancies of potential clinical and organizational relevance.
4. To develop an exploratory set of patient-centred, implementation-oriented recommendations for PM&R care in PFDs and to assess their perceived implementation feasibility through a modified Real-Time Delphi process.

## 3. Materials and Methods

### 3.1. Study Design and Context

This study was conducted using the **Delphi-Dialogue Patients–Professionals (DDPP)** framework a structured sequential methodology designed to evaluate the degree of alignment between patients and healthcare professionals and to translate identified perceptual differences into candidate service-improvement recommendations whose perceived implementation feasibility could subsequently be assessed. The study focused on the quality of Physical Medicine and Rehabilitation (PM&R) care for pelvic floor dysfunctions (PFDs) in Spain.

The project was coordinated by the Spanish Society of Physical Medicine and Rehabilitation (SERMEF) and supervised by a multidisciplinary steering committee comprising PM&R specialists with expertise in pelvic floor rehabilitation, methodology experts, and digital health researchers. External content review was conducted by a panel of PM&R clinicians with recognized expertise in PFD and by patient representatives from the Spanish Incontinence Association (ASIA). Their role was to assess the clinical relevance, clarity, comprehensibility, and acceptability of the study materials and to ensure that the patient perspective was incorporated during questionnaire development. This review provided evidence of face and content validity but did not constitute formal psychometric validation.

The study comprised a preparatory stage followed by two sequential analytical phases. During the preparatory stage (May 2022–January 2023), the research team developed two parallel questionnaires—one for patients and one for healthcare professionals—which underwent iterative expert review and content validation to ensure clarity, clinical relevance, and comparability across both respondent groups.

Phase 1 (February–April 2023) consisted of the nationwide administration of the questionnaires and the comparative analysis of patient and professional responses. This phase aimed to identify areas of convergence and divergence across multiple domains of rehabilitation care, including access to services, communication, patient education, continuity of care, adherence support, and digital rehabilitation.

Based on these findings, Phase 2 (May 2024) involved the development of a set of candidate recommendations addressing the principal areas of patient–professional perceptual divergence and selected areas of alignment identified in Phase 1. These recommendations were subsequently evaluated through a modified Real-Time Delphi process, in which a predominantly clinician-based panel assessed their perceived implementation feasibility through an iterative rating procedure involving anonymized feedback and within-session rescoring.

**Figure 1:**
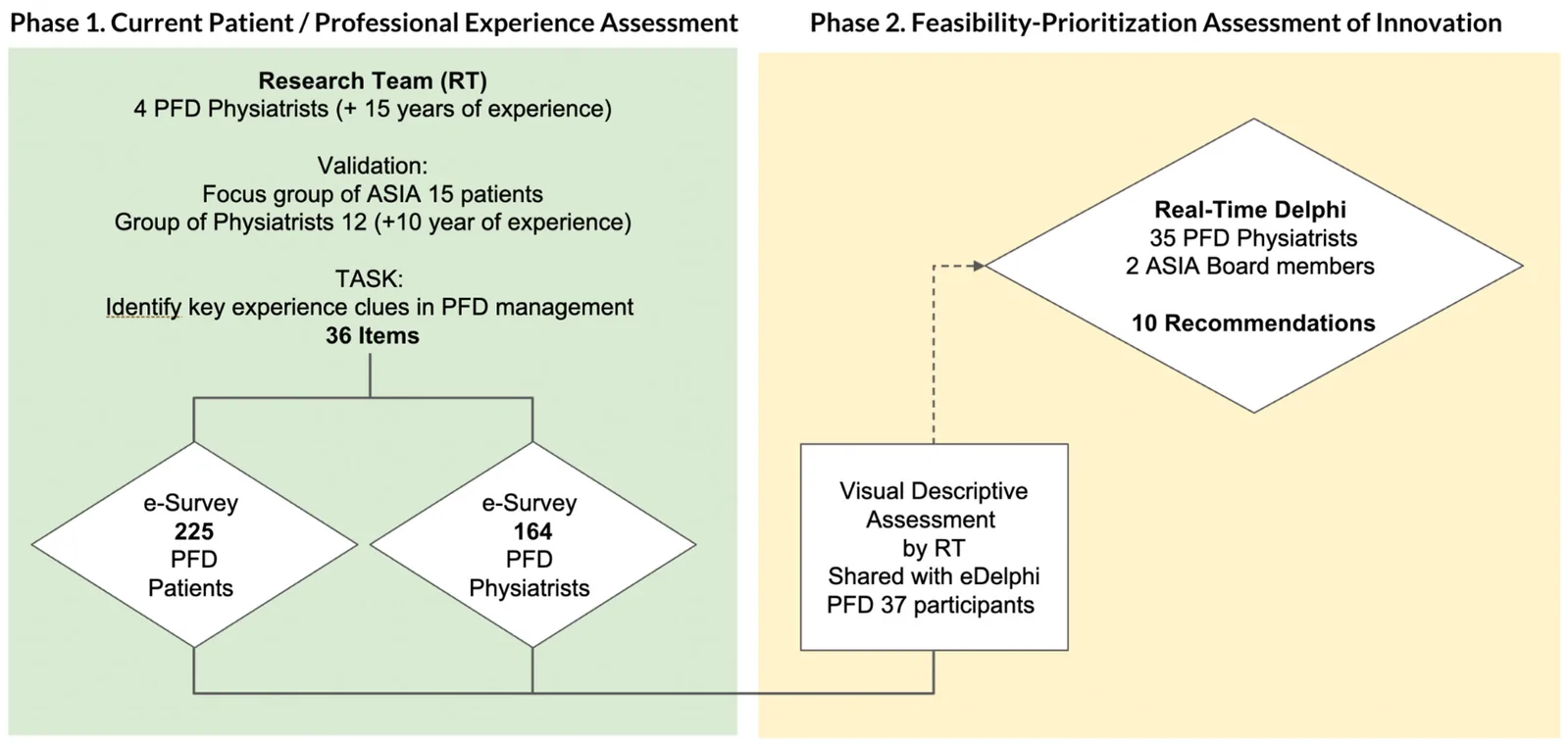
Study workflow: preparatory stage, Phase 1 survey-based patient–professional comparison, and Phase 2 Real-Time Delphi-based feasibility assessment.

This sequential design enabled the study to progress from the development of content-reviewed parallel questionnaires, through the descriptive comparison of stakeholder perspectives, to the generation of exploratory recommendations and the assessment of their perceived implementation feasibility, Figure 1.

### 3.2. Survey Development and Structure

Two parallel online questionnaires—one for patients and one for healthcare professionals—were specifically developed for this study by the DDPP-PFD research team. Questionnaire development was coordinated by four senior Physical Medicine and Rehabilitation (PM&R) physicians with expertise in pelvic floor dysfunction (PFD), with input from the multidisciplinary DDPP steering committee.

Initial items were generated from three sources: a review of the literature on pelvic floor rehabilitation and patient-centred care, the clinical experience of the participating PM&R specialists, and questionnaires previously developed within the DDPP framework. The purpose was to cover organizational and experiential aspects of rehabilitation care rather than to construct a unidimensional psychometric scale.

The draft questionnaires underwent a two-stage review process. First, a panel of 12 PM&R physicians, each with more than 10 years of experience in pelvic floor rehabilitation, reviewed the items for clinical relevance, completeness, appropriateness, and consistency with routine rehabilitation practice. Second, 16 patient representatives recruited through the Spanish Incontinence Association (ASIA) from different autonomous communities in Spain reviewed the patient questionnaire for clarity, comprehensibility, acceptability, and relevance to patients’ experiences.

Comments from both review groups were examined by the research team and used to clarify wording, remove ambiguity, adapt clinical terminology to patient-facing language, and ensure that the main dimensions of pelvic floor rehabilitation were adequately represented. The review process was iterative, and revised wording was discussed within the research team before the final questionnaires were approved for nationwide dissemination.

The questionnaires comprised three types of items: participant-characterization variables, general rehabilitation-care questions, and condition-specific questions. Together, they covered multidisciplinary care, access to specialized rehabilitation services, treatment adherence, timing and adequacy of information, self-management support, group-based interventions, remote communication, telemedicine, and digital health. Thirty-six conceptually matched items were available for direct patient–professional comparison and constituted the analytical dataset used for the concordance analyses. Participant-characterization variables, conditional follow-up questions, and stakeholder-specific items without an equivalent counterpart were excluded from the paired analyses.

All evaluative items were rated using a six-point Likert scale ranging from 1 (very poor/very low) to 6 (excellent/very high). An even-numbered scale was selected to avoid a neutral midpoint and encourage respondents to express a directional judgement.

The review process provided evidence of face and content validity. However, no independent pilot study or formal psychometric validation was conducted before nationwide administration. No data were therefore available on construct validity, factor structure, measurement invariance, internal consistency, test–retest reliability, or responsiveness. This decision reflected the implementation-oriented purpose and heterogeneous item content of the questionnaires, which were designed to compare stakeholder perceptions across distinct aspects of care rather than to measure a single latent construct. The complete patient and professional questionnaires are provided as Supplementary Files S1 and S2.

### 3.3. Participants and Recruitment

The study included two target populations:

(1) Patients. Eligible participants were adults aged ≥18 years who reported having a diagnosis of pelvic floor dysfunction (PFD). Previous assessment by a Physical Medicine and Rehabilitation (PM&R) service or receipt of rehabilitation or physiotherapy treatment was recorded as a participant characteristic but was not required for inclusion. Patients were recruited between February and April 2023 through two non-probability recruitment pathways: national patient organizations, principally the Spanish Incontinence Association (ASIA), and specialized pelvic floor rehabilitation units. This recruitment strategy was intended to include participants with different pelvic floor conditions and varying levels of exposure to rehabilitation services. However, the non-probability sampling procedure does not support statistical representativeness of the broader Spanish population with PFD.
(2) Healthcare professionals. Eligible professionals included PM&R physicians and physiotherapists actively involved in the assessment or management of patients with PFD. Participants were recruited between February and April 2023 through SERMEF-associated professional and clinical networks. The invitation provided access to an anonymous online questionnaire and was disseminated among professionals working in different healthcare settings. Because recruitment was voluntary and based on professional networks, the resulting sample was not intended to be statistically representative of all pelvic floor rehabilitation professionals in Spain.

Participation was voluntary, anonymous, and uncompensated. All respondents completed the questionnaires through a secure web-based platform after providing electronic informed consent. Eligibility was verified through initial screening questions, and completion of the respondent profile section was mandatory before access to the remaining survey items was granted.

Because recruitment was conducted through open organizational, professional, and clinical networks rather than through a closed sampling frame, the total number of individuals who received or viewed the survey invitation was not recorded. Consequently, recruitment denominators and conventional response rates could not be calculated for either respondent group. In addition, because participation was anonymous and individuals could potentially have been reached through more than one dissemination pathway, overlap between recruitment channels could not be quantified.

The final study sample comprised 225 patients with PFD and 164 rehabilitation professionals. The demographic, clinical, and professional characteristics of both study populations are presented in the Results section.

### 3.4. Procedure

The DDPP-PFD study followed a predefined sequential workflow encompassing questionnaire development and content validation, nationwide survey administration, descriptive and comparative analyses, formulation of preliminary recommendations by the research team, and subsequent evaluation of their implementation feasibility through a modified Real-Time Delphi feasibility-assessment process.

Survey responses were aggregated and analysed to compare patient and professional perspectives across parallel questionnaire items, thereby identifying areas of convergence and divergence relevant to the optimization of rehabilitation service delivery. Particular attention was given to domains with direct implications for clinical practice and healthcare organization, including access to specialized pelvic floor rehabilitation services, communication strategies, adherence support, and the integration of telemedicine and digital health technologies into long-term follow-up.

### 3.5. Outcomes and Concordance Analysis

The primary analytical objective was to characterize the degree of alignment and divergence between patients and rehabilitation professionals across the items shared by both questionnaires.

Descriptive statistics were calculated for each survey item and respondent group, including the mean (μ), standard deviation (σ), and proportion of high-agreement responses (A), defined as ratings within the two highest categories of the six-point Likert scale (scores 5–6). For analytical purposes, A was calculated as a proportion and is presented as a percentage in the corresponding tables.

To facilitate the identification and prioritization of patient–professional discrepancies, an operational divergence score (d) was calculated by combining two complementary measures: a standardized difference index (SDI), reflecting differences in mean ratings, and the relative difference in high-agreement proportions (ΔA), reflecting asymmetry in strong endorsement between stakeholder groups.

SDI = |μ_pat − μ_pro|/√[(σ^2^_pat + σ^2^_pro)/2]

A = |(A_pat − A_pro)|/min(A_pat, A_pro)

d = SDI × ΔA

The divergence score was developed as an exploratory, implementation-oriented prioritization metric rather than a validated effect-size or agreement measure. Its purpose was to summarize two complementary features of patient–professional divergence: SDI captures the magnitude of the difference in mean ratings relative to within-group variability, whereas ΔA captures the relative asymmetry between groups in the proportion of respondents expressing high agreement.

The two components were combined multiplicatively so that high divergence scores would arise primarily when both dimensions indicated disagreement. Accordingly, an item with a difference in mean ratings but little difference in high-agreement proportions, or vice versa, would receive a lower composite score. This formulation was intended to prioritize discrepancies that were simultaneously evident in central tendency and in the strength of endorsement, rather than to estimate a conventional statistical effect.

Because ΔA uses the smaller high-agreement proportion as its denominator, the score may increase substantially when high agreement is uncommon in one of the groups. Therefore, d is sensitive to low values of min (A_pat, A_pro) and must be interpreted together with the corresponding means, standard deviations, and high-agreement proportions reported in the Results section. Alternative approaches, including absolute mean differences, absolute differences in high-agreement proportions, ordinal comparisons, and conventional agreement or effect-size statistics, provide different information and may be preferable for confirmatory or inferential purposes. In the present study, d was used only as a transparent exploratory tool for ordering implementation-relevant discrepancies.

Importantly, the divergence score does not introduce additional information beyond the descriptive statistics reported for each item. Rather, it provides a structured way of integrating differences in mean ratings and high-agreement responses into a single implementation-oriented ordering of items. The principal descriptive findings should therefore be interpreted primarily in light of the underlying descriptive statistics rather than the composite score itself.

Bootstrap confidence intervals, ordinal regression models, and item-level non-parametric tests were not included because the present analysis was based on the predefined aggregated outputs of the DDPP comparison. These approaches require individual-level item-response data and additional assumptions regarding the ordinal response distributions. The sensitivity analysis was therefore restricted to alternative descriptive measures directly derived from the statistics reported in the Results section.

As a descriptive sensitivity analysis, two simpler measures were calculated for each item: the absolute mean difference (AMD), defined as |μ_pat − μ_pro|, and the absolute high-agreement gap (AHG), defined as |A_pat − A_pro| and expressed in percentage points. These measures were examined alongside d to assess whether the main descriptive pattern was dependent on the multiplicative formulation of the composite score.

### 3.6. Recommendation Development Process

Following completion of the Phase 1 patient–professional comparison, the multidisciplinary DDPP steering committee developed an initial set of implementation-oriented recommendations. The committee included PM&R specialists with expertise in pelvic floor rehabilitation, methodology researchers, and digital health researchers.

Recommendation development followed a structured interpretative process. First, the survey items were reviewed according to their clinical meaning, position within the rehabilitation pathway, direction and magnitude of patient–professional divergence, and potential relevance to service organization. Second, items addressing related aspects of care were grouped into broader implementation domains. Third, each group of items was translated into a candidate recommendation intended to represent an actionable service-improvement strategy rather than a direct restatement of an individual questionnaire item.

The divergence score was not used as the sole criterion for generating recommendations. The committee also considered the clinical relevance of each item, conceptual coherence between grouped items, organizational applicability, and the presence of areas of stakeholder alignment that could facilitate implementation. For this reason, some recommendations included both higher- and lower-divergence items.

Draft recommendations were reviewed within the steering committee. Differences in interpretation were discussed until agreement was reached on the wording, scope, and survey-item mapping of each recommendation. This process resulted in 10 candidate implementation-oriented recommendations that were submitted to the modified Real-Time Delphi feasibility assessment.

The recommendations were not independently co-designed or formally approved by a separate patient panel before the Delphi phase. Patient input during this stage was provided through the participation of two representatives from the Spanish Incontinence Association (ASIA) in the feasibility assessment. This limited level of patient involvement should be considered when interpreting the stakeholder scope of the resulting recommendations.

The Real-Time Delphi phase assessed the perceived implementation feasibility of the 10 pre-formulated recommendations. It was not designed to generate additional recommendations or to modify their substantive content. Anonymized feedback and within-session rescoring could influence their relative implementation feasibility, but the wording and survey-item mapping of the 10 recommendations remained unchanged.

### 3.7. Real-Time Delphi Feasibility Assessment

In May 2024, a modified Real-Time Delphi process was conducted to evaluate the implementation feasibility of the recommendations derived from Phase 1. The methodology combined synchronous participation, real-time anonymized feedback, and within-session rescoring, allowing participants to iteratively refine their assessments while preserving the independence of individual responses.

The Delphi panel comprised 37 participants: 35 Physical Medicine and Rehabilitation (PM&R) physicians with experience in pelvic floor rehabilitation and two representatives from the Board of the Spanish Incontinence Association (ASIA). Participants were selected purposely according to their clinical expertise, involvement in pelvic floor rehabilitation services, or formal role in representing the patient perspective. The two ASIA representatives contributed an organized patient perspective to the feasibility assessment, but their inclusion was not intended to provide numerical balance between patients and professionals. Consequently, the panel was predominantly clinician-based, and its assessments primarily reflect the perspective of PM&R physicians.

No other healthcare professionals participated in the Delphi feasibility phase, although physiotherapists represented a substantial proportion of the professional respondents in Phase 1. The Phase 2 panel was convened through the PM&R specialist network coordinating the study and was designed to assess the perceived organizational feasibility of the pre-formulated recommendations within PM&R services. The absence of physiotherapists limits the multidisciplinary scope of the feasibility assessment and should be considered when interpreting the results.

The Delphi panel was conceived as a predominantly clinician-based, implementation-focused feasibility panel rather than as a balanced stakeholder panel or a continuation of the Phase 1 survey cohort. Although some experts had previously participated in earlier stages of the DDPP project, the Delphi exercise constituted an independent evaluation centred exclusively on the feasibility of implementing the proposed recommendations rather than on reassessing the original survey responses. Before the scoring exercise, participants received a structured summary of the Phase 1 findings and the 10 candidate recommendations developed through the process described in Section 3.6. The summary included the descriptive patient–professional comparisons, the principal areas of convergence and divergence, and the survey items linked to each recommendation.

The Delphi session was conducted using the SmartDelphi platform. Participants independently rated the implementation feasibility of each recommendation on a six-point Likert scale ranging from 1 (very difficult to implement) to 6 (very easy to implement). Following the initial scoring round, anonymized group results were displayed in real time, enabling participants to compare their individual ratings with the overall distribution of responses. Participants were subsequently given the opportunity to revise their scores during the same session. This iterative procedure represents a modified Real-Time Delphi approach combining synchronous participation, anonymous group feedback, and immediate rescoring to support reflection on the perceived feasibility of the recommendations. The session concluded once all recommendations had been evaluated, group feedback had been reviewed, and no further score modifications were submitted within the allocated time. The analysis was based on the final feasibility ratings; intermediate rating trajectories, rescoring frequency, and changes between initial and final ratings were not analysed. Final feasibility ratings were summarized descriptively using the mean, standard deviation, median, and interquartile range for each recommendation.

The objective of this phase was to assess and prioritize the perceived implementation feasibility of the 10 pre-formulated recommendations, rather than to generate new recommendations or revise their substantive content. The wording and linked survey items remained unchanged throughout the session; only participants’ feasibility ratings could be reconsidered after anonymized group feedback.

These indicators were subsequently interpreted alongside the corresponding patient–professional divergence scores, enabling each recommendation to be positioned according to two complementary dimensions: the magnitude of the identified patient–professional perceptual divergence and the perceived implementation feasibility of the corresponding recommendation.

Because the objective of the Delphi exercise was to prioritize implementation rather than to establish formal consensus, no predefined consensus threshold was applied. Accordingly, the results should be interpreted as measures of perceived implementation feasibility rather than formal consensus statements, Table 1.

### 3.8. Ethics and Data Protection

This study was conducted in accordance with the principles of the Declaration of Helsinki and applicable Spanish and European data-protection requirements, including the General Data Protection Regulation (GDPR, EU 2016/679) [16].

The questionnaires collected self-reported health-related information, including pelvic floor symptoms, diagnoses, comorbidities, mobility limitations, and previous treatment. However, no names, contact details, medical-record identifiers, or other direct personal identifiers were collected. Participation was voluntary, and electronic informed consent was obtained after participants had received information about the study procedures, use of the data, and confidentiality safeguards.

The study consisted of anonymous, non-interventional online surveys and a modified Real-Time Delphi feasibility assessment. No external CEI/CEIm review was obtained. The research team considered that the activities performed did not fall within the categories requiring mandatory CEI/CEIm evaluation under the applicable regulatory framework; this assessment did not constitute a formal exemption issued by an independent ethics committee.

Data were collected through a secure web-based platform, analysed without direct identifiers, and reported only in aggregated form. SERMEF provided institutional coordination and an internal scientific review of the study protocol. This review focused on the methodological coherence and scientific relevance of the project and did not constitute independent ethics committee approval or a formal ethics-review exemption. During the Delphi phase, only anonymized group-level feedback was shared.

## 4. Results

The comparative analysis identified both areas of convergence and divergence between patients’ and rehabilitation professionals’ perceptions of pelvic floor rehabilitation care. The magnitude and direction of these differences varied across the different domains evaluated, indicating that stakeholder perspectives were closely aligned in some aspects of care while diverging substantially in others.

### 4.1. Participant Characteristics

A total of 225 patients with pelvic floor dysfunction (PFD) and 164 rehabilitation professionals completed the study questionnaires. The demographic, clinical, and professional characteristics of both study populations are summarized in Table 2 and Table 3.

The patient sample was predominantly female (88.0%) and had a comparatively high educational profile, with 49.8% reporting university studies and 35.1% reporting secondary education or vocational training. Previous exposure to rehabilitation services was heterogeneous: 80.4% had previously been assessed by PM&R for PFD, whereas 71.6% reported having received rehabilitation or physiotherapy treatment. The professional sample was also predominantly female (93.9%) and comprised 101 PM&R physicians and 63 physiotherapists working across different hospital settings.

Because not all patient respondents had previously been assessed by PM&R or received rehabilitation or physiotherapy treatment, patient responses reflect different levels of direct exposure to pelvic floor rehabilitation services. This heterogeneity should be considered when interpreting items specifically referring to rehabilitation delivery and follow-up.

### 4.2. Comparison of Experiences Between Patients and Professionals

Patient–professional comparisons for all parallel survey items are presented in Table 4, with items ranked according to the magnitude of patient–professional divergence.

To facilitate interpretation, divergence was quantified using the composite divergence score (d) described in the Methods section. This operational indicator combines the standardized difference between mean ratings (SDI) with the relative difference in high-agreement responses (ΔA), thereby integrating differences in central tendency and in the strength of endorsement between stakeholder groups. As previously described, d is intended as an implementation-oriented prioritization metric rather than a conventional statistical effect size.

Values of d close to zero indicate limited divergence according to the composite metric, generally because differences in mean ratings, high-agreement proportions, or both are small. Conversely, higher values indicate progressively greater divergence arising from differences in stakeholder ratings, asymmetry in high-agreement responses, or both. Because d is calculated as the product of SDI and ΔA, it should be interpreted together with its constituent descriptive statistics rather than as a standalone measure of agreement.

As shown in Table 4, the largest patient–professional perceptual divergences were observed for items related to perceived digital competence, the timing of information delivery, the perceived usefulness of and difficulty completing bladder and bowel diaries, and reported adherence to prescribed rehabilitation programmes. Intermediate levels of perceptual divergence were identified for selected aspects of psychosocial support and group-based rehabilitation interventions. In contrast, hybrid models of care, written and video-assisted exercise instructions, and several aspects of rehabilitation follow-up exhibited low divergence scores, indicating comparatively similar response patterns between patients and rehabilitation professionals.

The descriptive sensitivity analysis showed that the overall prioritization pattern was not restricted to the multiplicative divergence score. Rankings based on the absolute mean difference and the absolute high-agreement gap showed a broadly similar descriptive pattern to the ranking based on d, although the exact ordering of individual items differed between measures.

Accordingly, the principal interpretation of the study did not depend exclusively on the proposed divergence score. The composite metric was used only to facilitate a transparent prioritization of descriptive differences that were also apparent when simpler descriptive measures were examined.

As a descriptive sensitivity analysis, the item-level results obtained with the composite divergence score were examined alongside the absolute mean difference (AMD) and the absolute high-agreement gap (AHG). The alternative measures showed a broadly similar descriptive pattern, with digital competence, timing of information delivery, bladder and bowel diary use, adherence, and selected psychosocial and group-based interventions remaining among the most prominent patient–professional discrepancies. However, the exact ordering of individual items varied according to the measure used.

### 4.3. Second Phase: Structuring and Prioritization of Recommendations

Based on the patient–professional discrepancies identified in Phase 1, the steering committee developed 10 recommendations aimed at improving the organization and delivery of pelvic floor rehabilitation services using the structured development process described in Section 3.6. Rather than mirroring individual survey items, each recommendation synthesized conceptually related findings according to their clinical relevance, position within the rehabilitation pathway, patient–professional divergence, organizational applicability, and potential relevance to service improvement, thereby generating clinically meaningful and actionable proposals. These recommendations were pre-formulated before the modified Real-Time Delphi and subsequently evaluated for their perceived implementation feasibility by a predominantly clinician-based panel comprising 37 participants: 35 PM&R physicians and two representatives from the Spanish Incontinence Association (ASIA). For exploratory descriptive purposes, each recommendation was positioned according to two complementary dimensions: (i) the mean patient–professional divergence associated with its linked survey items and (ii) its perceived implementation-feasibility rating obtained during the modified Real-Time Delphi assessment.

As illustrated in Table 5, recommendations combining comparatively higher divergence with higher perceived implementation-feasibility ratings may represent candidate areas for future implementation initiatives. These included recommendations related to adherence monitoring, the visibility and accessibility of pelvic floor rehabilitation pathways, and digital applications supporting patient follow-up.

Recommendations characterized by comparatively higher divergence but lower perceived feasibility, such as digital health-literacy initiatives, may require broader organizational or institutional support before implementation can be evaluated. Conversely, recommendations exhibiting lower divergence and higher perceived feasibility—including home-based exercise programmes and standardized patient-education materials—may identify areas in which existing practices could be examined for more consistent application.

**Figure 2:**
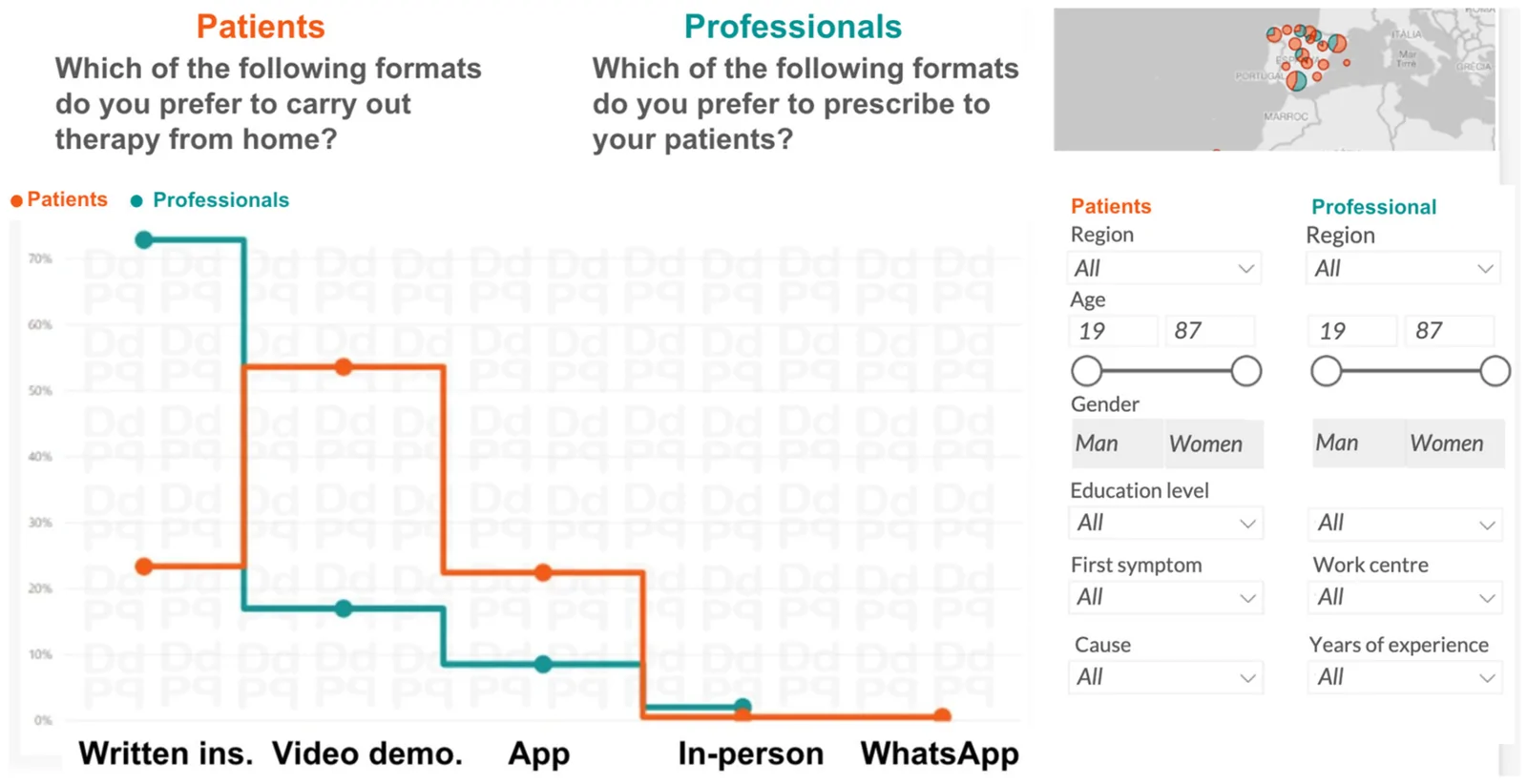
Representative example of the analytical information presented to participants during the modified Real-Time Delphi feasibility assessment. The figure is included to document the methodological workflow used during the DDPP process.

**Figure 3:**
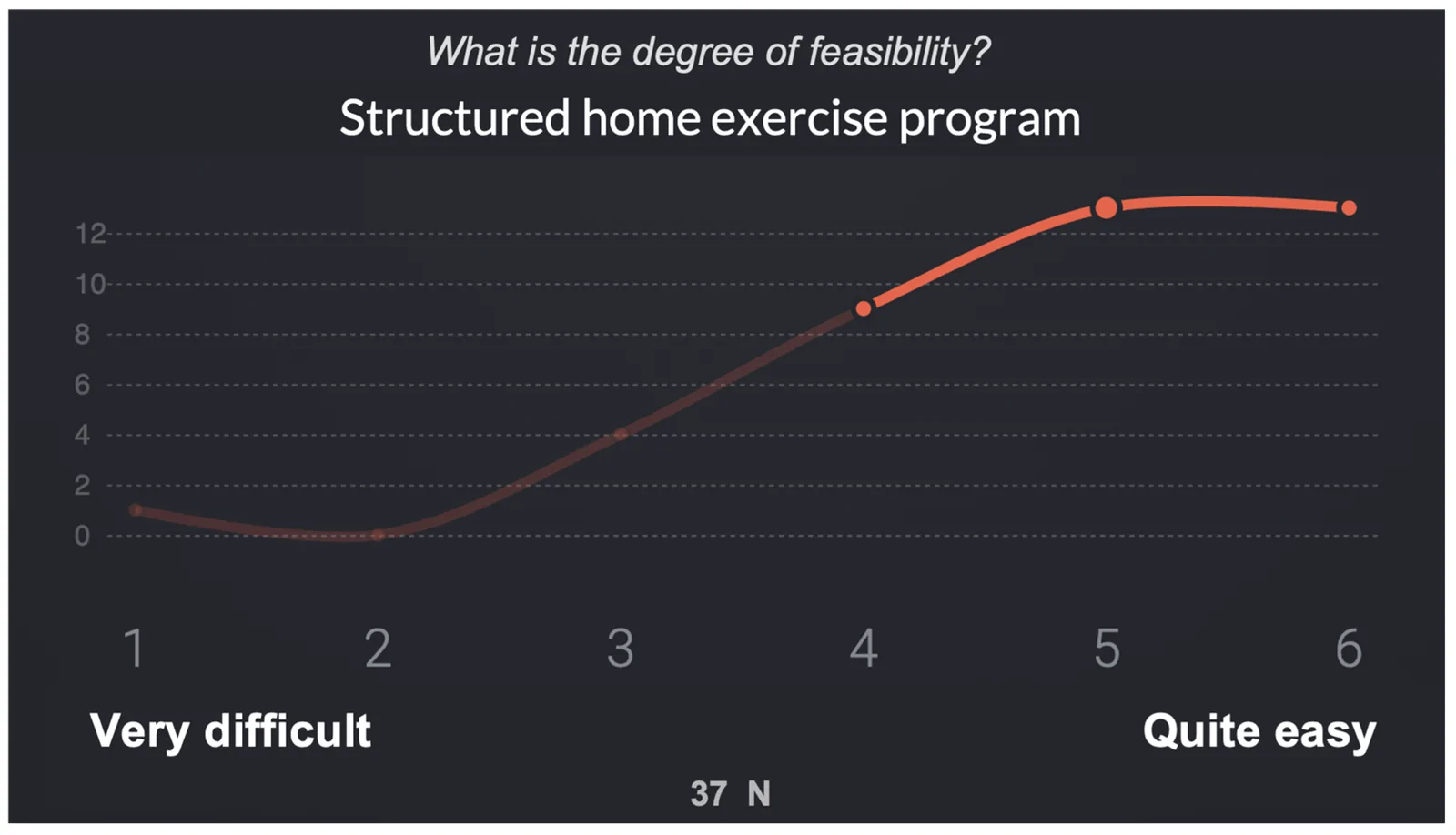
Representative example of the SmartDelphi interface used during the synchronous modified Real-Time Delphi session. The figure is provided to document the methodological implementation of the feasibility-assessment procedure rather than to present study results.

Figure 2 and Figure 3 illustrate, respectively, the analytical feedback shared with participants and the SmartDelphi interface 2024 version (www.smartdelphi.com) used to support real-time anonymized feedback and within-session rescoring.

**Figure 4:**
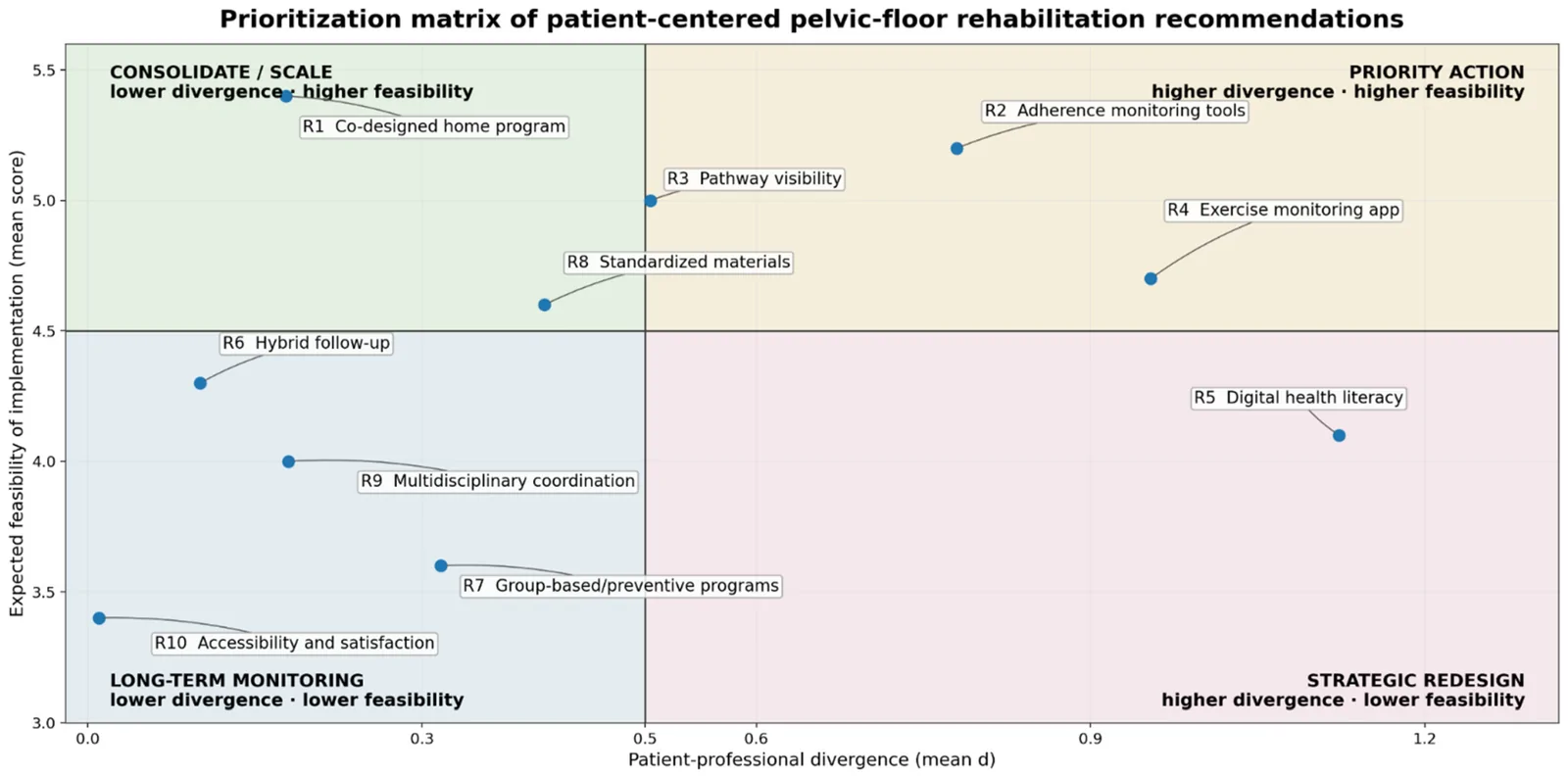
Exploratory descriptive matrix of candidate pelvic floor rehabilitation recommendations according to mean divergence and perceived implementation feasibility. Cut-offs: divergence = 0.50|feasibility = 4.5.

Table 5 provides a complete traceability pathway linking the Phase 1 survey items and their principal divergence patterns to the 10 candidate recommendations, their mean divergence values, and the feasibility ratings obtained during the modified Real-Time Delphi assessment, Figure 4.

Table feasibility threshold of 4.5 was selected to identify recommendations with comparatively higher perceived feasibility within the six-point feasibility scale.

The divergence threshold of 0.50 was selected pragmatically to distinguish recommendations with higher operational divergence within the observed distribution while preserving interpretability of the prioritization matrix.

These thresholds were selected pragmatically for visual stratification purposes only and were not intended as validated decision rules.

### 4.4. Exploratory Strategic Innovation Domains in Pelvic Floor Rehabilitation

Building on the comparative analysis and the perceived implementation-feasibility assessment of the candidate recommendations, the research team organized the findings into four exploratory Strategic Innovation Domains (IDs). These domains provide a conceptual synthesis of patient–professional perceptual divergence, comparatively similar response patterns, and perceived implementation feasibility. They should be interpreted as an exploratory framework for informing future service-development and implementation research within Physical Medicine and Rehabilitation (PM&R) services.

**ID1. Improving Access, Visibility, and Organizational Integration.** Improving the visibility and organizational integration of pelvic floor rehabilitation within PM&R services emerged as a candidate area for future implementation research. This domain encompasses recommendations aimed at streamlining referral pathways, expanding access to specialized rehabilitation services, strengthening multidisciplinary collaboration, and improving continuity of care throughout the rehabilitation pathway. More structured, coordinated, and traceable communication processes may reduce fragmentation, facilitate navigation across healthcare levels, and enhance patients’ confidence in rehabilitation services.

**ID2. Strengthening Education, Self-Management, and Long-Term Adherence.** The effectiveness of pelvic floor rehabilitation depends largely on patients’ ability to maintain long-term engagement with therapeutic programmes. This domain emphasizes the implementation of structured home-based exercise programmes, standardized educational resources, and structured adherence-monitoring strategies tailored to patients’ functional needs and rehabilitation goals. The findings suggest that optimizing adherence requires more than prescribing exercises alone; it also demands continuous educational support, progressive self-management strategies, and individualized follow-up capable of identifying and addressing barriers to sustained participation.

**ID3. Advancing Digital Health and Hybrid Rehabilitation Models.** The survey findings showed comparatively similar patient and professional ratings regarding hybrid rehabilitation pathways combining face-to-face care with remote follow-up.

This domain prioritizes the development of structured tele-rehabilitation programmes, digital monitoring platforms, and interventions designed to strengthen digital health literacy among both patients and healthcare professionals. Importantly, the findings support a gradual, patient-centred integration of digital technologies in which remote tools complement, rather than replace, conventional rehabilitation. Successful implementation will depend on ensuring usability, accessibility, and alignment with patients’ capabilities, preferences, and clinical needs.

**ID4. Promoting Personalized, Preventive, and Participatory Rehabilitation.** Pelvic floor rehabilitation extends beyond symptom management and requires personalized strategies capable of adapting to functional status, psychosocial burden, motivation, and previous rehabilitation experience. This domain promotes preventive interventions, personalized rehabilitation pathways, group-based programmes, and participatory models that actively incorporate patients’ experiences into continuous service improvement. Embedding prevention, personalization, and patient partnership within rehabilitation pathways may facilitate more proactive, adaptive, and value-based models of pelvic floor care.

## 5. Discussion

### 5.1. Principal Findings and Contribution Beyond Existing Evidence

This study provides an exploratory, stakeholder-informed analysis of perceptions of pelvic floor rehabilitation care by comparing the perspectives of patients and rehabilitation professionals and subsequently assessing the perceived implementation feasibility of candidate recommendations through a modified Real-Time Delphi process. Its principal contribution lies in describing areas of patient–professional perceptual convergence and divergence and organizing these findings into exploratory Strategic Innovation Domains. These domains may inform future implementation studies and service-development initiatives but should not be interpreted as validated priorities or as evidence of improved care quality.

Although the clinical effectiveness of pelvic floor rehabilitation has been consistently demonstrated in randomized controlled trials and is strongly supported by international clinical practice guidelines [4,5,6], substantially less attention has been devoted to understanding how rehabilitation is perceived, experienced, and implemented in routine clinical practice. The present findings identify differences in how patients and professionals reported or evaluated selected components of the rehabilitation process. The study did not measure clinical outcomes and therefore cannot determine whether patient–professional perceptual divergence affects treatment effectiveness.

The greatest patient–professional discrepancies were observed in domains closely associated with long-term treatment success, including digital competence, the timing and adequacy of information delivery, the use of self-monitoring tools, and adherence to prescribed rehabilitation programmes. More moderate differences emerged in relation to psychosocial support and group-based interventions, whereas several aspects of hybrid rehabilitation models and educational support showed comparatively high levels of agreement between stakeholder groups. These observations are consistent with evidence from other rehabilitation settings in which patient–professional differences have been associated with adherence, patient engagement, and continuity of care [13]. However, the present observational study cannot establish whether the identified perceptual divergences caused reduced adherence, poorer clinical outcomes, or deficiencies in care quality. The findings instead identify areas of care delivery, communication, and self-management that may warrant further investigation.

### 5.2. Patient–Professional Divergences: Implications for Adherence and Self-Management

One of the most clinically relevant findings of this study was the identification of substantial patient–professional divergence in domains closely related to self-management, digital competence, and the perceived usefulness of specific rehabilitation strategies. The greatest discrepancies were observed for items concerning bladder and bowel diaries, digital competence, and adherence to prescribed rehabilitation programmes (Table 4), highlighting areas in which patients and professionals appear to assign different values to key components of the rehabilitation process.

These findings suggest that interventions supported by clinical evidence are not necessarily perceived by patients as equally useful, acceptable, or sustainable in everyday life. This distinction is particularly important in pelvic floor rehabilitation, where therapeutic success depends largely on patients’ long-term engagement with home-based exercise programmes and behavioural interventions. Consequently, the effectiveness of rehabilitation cannot be understood solely in terms of intervention efficacy but must also consider how rehabilitation strategies are experienced, understood, and incorporated into daily routines.

Consistent with previous studies, adherence remains one of the strongest determinants of successful pelvic floor muscle training and long-term functional outcomes [6]. However, the present findings reinforce the view that adherence should not be regarded simply as a patient-related behavioural characteristic. Rather, it emerges from the dynamic interaction between intervention design, clinician–patient communication, educational support, and the patient’s lived experience throughout the rehabilitation pathway.

### 5.3. Timing of Information Delivery and Communication: An Implementation Challenge

A second important area of patient–professional divergence concerned the timing and perceived adequacy of information delivery. Unlike most other domains, this discrepancy showed an inverse pattern: patients generally considered that information had been provided at an appropriate stage of their rehabilitation pathway, whereas rehabilitation professionals expressed a more critical appraisal of this aspect of care.

Rather than indicating deficiencies in access to information itself, this finding appears to reflect different standards by which patients and professionals evaluate effective communication. Patients may judge information primarily according to whether it addressed their immediate concerns during the rehabilitation process, whereas professionals are more likely to assess communication against evidence-based recommendations and ideal models of patient education. Consequently, the observed divergence may reflect differences in expectations rather than genuine disagreement regarding the information actually delivered.

This observation is particularly relevant because contemporary clinical practice guidelines consistently identify patient education as a cornerstone of pelvic floor rehabilitation [4,5]. Nevertheless, translating these recommendations into routine clinical practice remains challenging. Educational interventions are effective only when information is delivered at appropriate moments throughout the rehabilitation pathway, reinforced over time, and adapted to patients’ evolving needs, health literacy, and readiness to engage in self-management.

### 5.4. Digital Health and Hybrid Rehabilitation: Broad Acceptance with Conditional Adoption

In contrast to the domains showing marked patient–professional divergence, the study identified substantial alignment regarding hybrid rehabilitation models, the use of written and video-based educational resources, and overall satisfaction with rehabilitation follow-up. These findings indicate that both patients and rehabilitation professionals broadly support integrating face-to-face care with digitally enabled follow-up as part of contemporary pelvic floor rehabilitation pathways.

This observation is consistent with a growing body of evidence demonstrating that tele-rehabilitation can effectively complement conventional rehabilitation when implemented within well-structured clinical pathways rather than as a stand-alone intervention [14,15]. However, the present findings also introduce an important nuance. Acceptance of digital health was not unconditional but appeared to depend on the complexity of the proposed intervention and the level of patient engagement required.

Although patients expressed positive attitudes towards digital resources that supported conventional rehabilitation—such as educational materials, remote follow-up, and structured communication—greater patient–professional divergence emerged in domains requiring more active digital participation, including self-monitoring and higher levels of digital competence. Previous reviews have similarly shown that mobile and digital health interventions may support self-management and home-based rehabilitation, although their effects depend on intervention design, professional involvement, user engagement, and integration into routine care [17,18,19]. Successful digital implementation therefore depends not only on technological availability but also on usability, perceived usefulness, digital confidence, and the extent to which digital tools are integrated into patients’ daily rehabilitation routines. The potential benefits of digital engagement should also be balanced against the emotional and self-regulatory demands that these interventions may place on people living with chronic conditions [20].

These findings informed exploratory Strategic Innovation Domain 3. Future implementation studies should examine the usability, accessibility, safety, adoption, and sustainability of digital and hybrid rehabilitation models adapted to patients’ capabilities and preferences.

### 5.5. Organizational Determinants: Access, Visibility, and Care Coordination

One of the most informative findings was the relatively high level of agreement between patients and rehabilitation professionals regarding access to specialized pelvic floor rehabilitation services, as reflected by the low divergence scores. However, this apparent concordance should not be interpreted as evidence of satisfactory service provision.

Instead, both stakeholder groups assigned only moderate ratings to access-related items, suggesting that they shared a similar perception of existing organizational limitations. This pattern may be interpreted as aligned dissatisfaction, whereby patients and professionals converge in recognizing systemic shortcomings rather than in expressing satisfaction with current service delivery. From an implementation perspective, this distinction is particularly relevant because agreement alone should not be equated with quality.

The findings suggest that barriers perceived by both patients and professionals may involve referral pathways, multidisciplinary coordination, and the visibility of PM&R services within broader pelvic floor care networks. These observations informed exploratory Strategic Innovation Domain 1 and identify organizational integration as an area for future service-development research.

### 5.6. Implications for Implementation and Service Redesign

The findings identify specific components of pelvic floor rehabilitation that may warrant further implementation research, particularly digital competence, patient education, self-monitoring, adherence support, and selected psychosocial and group-based interventions. Established implementation-science frameworks may help structure the future evaluation of these areas by examining perceived feasibility alongside contextual determinants, adoption, implementation fidelity, sustainability, and organizational integration [21].

The DDPP framework provides an exploratory method for combining patient–professional perceptual differences with assessments of implementation feasibility. This approach may support the formulation of candidate service-development strategies, but it does not determine whether these strategies will be successfully adopted or improve clinical outcomes.

Future studies should prospectively evaluate the acceptability, feasibility, effectiveness, sustainability, and resource implications of the proposed recommendations in routine clinical settings. Incorporating patient-relevant outcomes, clinical outcomes, and resource use would also allow future evaluations to examine whether these strategies contribute to value-based models of rehabilitation care [22]. Their transferability to other rehabilitation populations and healthcare systems requires independent assessment.

## 6. Limitations

Several limitations should be considered when interpreting the findings of this study. These limitations relate to the study instruments and self-reported data, the cross-sectional design, the exploratory analytical approach, participant selection, the composition of the Delphi panel, the clinical heterogeneity of the patient sample, and the generalizability of the findings.

### 6.1. Instruments and Self-Reported Data

All Phase 1 data were based on self-reported responses. Patients evaluated their personal experiences and perceptions of rehabilitation, whereas professionals reported their usual clinical practice or their perceptions of care delivery. No objective measures of adherence, treatment completion, symptom severity, healthcare utilization, functional improvement, quality of care, or clinical outcomes were collected. The identified patient–professional divergences should therefore be interpreted as differences in reported perceptions or experiences rather than as evidence of differences in clinical effectiveness or objective service quality.

Although the patient and professional questionnaires were intentionally developed as parallel instruments, complete conceptual and measurement equivalence between all matched items cannot be assumed. The comparisons involved patients’ reported experiences and professionals’ reported practices or perceptions, and some observed differences may consequently reflect the distinct perspectives from which the same component of care was evaluated.

The questionnaires underwent iterative review by experienced PM&R physicians and patient representatives, providing evidence of face and content validity. However, they were not subjected to an independent pilot study or formal psychometric validation. Construct validity, factor structure, measurement invariance, internal consistency, test–retest reliability, and responsiveness were not assessed. Because the questionnaires covered heterogeneous components of rehabilitation care rather than a single latent construct, a global internal-consistency coefficient would not necessarily have been an appropriate indicator of instrument quality.

The findings should therefore be regarded as exploratory item-level comparisons obtained using implementation-oriented instruments whose psychometric properties remain to be established. Future studies should formally evaluate their content-validity indices, construct validity, stability, and cross-group measurement performance before broader use.

### 6.2. Cross-Sectional Design

Phase 1 used a cross-sectional design and captured patient and professional responses during a single study period. The study cannot therefore establish temporal relationships, causal pathways, or changes in perceptions over the course of rehabilitation.

In particular, it cannot determine whether the identified perceptual divergences preceded, resulted from, or influenced adherence, healthcare use, patient experience, continuity of care, or clinical outcomes. The patient and professional samples were also independent respondent groups rather than matched patient–clinician pairs. Consequently, the analysis reflects group-level differences and does not measure disagreement within individual therapeutic relationships.

### 6.3. Exploratory Divergence Index

The composite divergence score (d) was developed as an exploratory operational indicator to facilitate the descriptive ordering of patient–professional differences. It should not be interpreted as a validated effect size, concordance measure, or inferential statistic.

Its multiplicative structure gives greater prominence to items showing both standardized differences in mean ratings and relative differences in high-agreement responses. However, because ΔA uses the smaller high-agreement proportion as its denominator, the score may be particularly sensitive when high agreement is uncommon in one of the respondent groups. The score should therefore be interpreted alongside its individual components and the descriptive statistics reported in Table 4 rather than as a standalone measure.

A descriptive sensitivity analysis using absolute mean differences and absolute high-agreement gaps showed a broadly similar overall pattern, although the exact ordering of individual items varied according to the measure used. The divergence score has not undergone external validation, and no bootstrap uncertainty estimates, ordinal regression models, item-level non-parametric comparisons, or systematic comparisons with established agreement statistics were performed.

Future methodological studies should assess the robustness, comparative performance, and appropriate applications of this exploratory metric.

### 6.4. Sampling and Selection Bias

Participants were recruited through a voluntary, non-probability sampling strategy involving patient associations, specialized pelvic floor rehabilitation units, SERMEF-associated professional networks, and participating clinical services.

Because the survey invitations were disseminated through open organizational, professional, and clinical channels, the number of individuals who received or viewed the invitation was not available. Consequently, recruitment denominators and conventional response rates could not be calculated. Potential overlap between recruitment pathways could not be quantified because survey participation was anonymous.

Selection bias therefore cannot be excluded. Respondents may have been more engaged with pelvic floor rehabilitation, patient organizations, digital communication, or participatory models of care than non-respondents.

Both respondent groups were predominantly female, half of the patient respondents had university-level education, and a high proportion of patients had previously been assessed by PM&R or had received rehabilitation or physiotherapy treatment. These characteristics may have influenced reported perceptions of access, communication, digital competence, adherence, and follow-up.

Accordingly, the samples should not be regarded as statistically representative of all individuals with pelvic floor dysfunction or all pelvic floor rehabilitation professionals in Spain.

### 6.5. Delphi-Panel Composition and Feasibility Assessment

The modified Real-Time Delphi assessed perceived implementation feasibility rather than actual implementation outcomes.

The panel was predominantly composed of PM&R physicians, with only two patient-association representatives and no physiotherapists or other healthcare professionals. Its ratings therefore primarily reflect the perspective of PM&R physicians and should not be interpreted as balanced multidisciplinary or multistakeholder consensus. The limited patient representation and the absence of other professional groups restrict the range of perspectives incorporated into the feasibility assessment.

Perceived feasibility cannot be assumed to predict successful adoption, sustainability, organizational impact, safety, clinical benefit, or cost-effectiveness under routine clinical conditions.

The Delphi exercise was also conducted approximately one year after the Phase 1 surveys. Changes in clinical practice, digital-health adoption, healthcare organization, or participants’ experience during this interval may have influenced the feasibility ratings.

Furthermore, the synchronous, within-session rescoring procedure differed from conventional Delphi designs based on multiple temporally separated rounds. It did not therefore evaluate the stability of participant ratings across separate assessment periods.

The modified Real-Time Delphi panel was intentionally composed of PM&R physicians because the objective of this phase was to evaluate the perceived feasibility of recommendations from the perspective of specialists responsible for coordinating rehabilitation pathways and service organization. Physiotherapists were not included in this panel, although they play a central role in the delivery of pelvic floor rehabilitation. Consequently, the feasibility assessments reported here reflect the perspective of one professional group and may not fully capture implementation priorities or practical considerations identified by physiotherapists. Accordingly, the panel was selected to capture the perspective of the professional group primarily responsible for organizational decision-making within the PM&R care pathway, rather than to achieve proportional representation of all rehabilitation stakeholders. The findings of this phase should therefore be interpreted as implementation-oriented feasibility judgments from this specific perspective. Future Delphi studies including multidisciplinary rehabilitation teams would strengthen the external validity of these findings.

### 6.6. Clinical Heterogeneity

The patient sample was clinically heterogeneous and included individuals reporting different pelvic floor conditions, principal symptoms, symptom durations, comorbidities, mobility limitations, and levels of previous exposure to PM&R, rehabilitation, or physiotherapy.

Participants could report more than one pelvic floor condition, and the results cannot therefore be attributed to a single diagnostic population. In addition, the study was not designed or powered to perform condition-specific or clinically stratified subgroup analyses.

The aggregated patient results may consequently conceal relevant differences between diagnostic or symptom groups, between previously treated and untreated participants, or according to symptom duration and clinical complexity.

Future studies should examine the identified perceptual patterns in larger and more clinically homogeneous patient subgroups.

No sensitivity analysis restricted to patients who had previously been assessed by PM&R or received rehabilitation or physiotherapy treatment was performed. Consequently, experience-based items may include responses from participants with different levels of direct exposure to the services or interventions evaluated.

The professional sample was also heterogeneous with respect to professional category, workplace, clinical experience, patient volume, and participation in multidisciplinary pelvic floor units. Exploratory subgroup analyses by professional category, type of institution, or multidisciplinary-unit status were not pre-specified and were not performed because the study was not designed or powered for these comparisons. The aggregated professional results may therefore conceal differences between PM&R physicians and physiotherapists or between different organizational settings.

### 6.7. Generalizability

The study was conducted within the Spanish Physical Medicine and Rehabilitation healthcare context and was coordinated through the Spanish Society of Physical Medicine and Rehabilitation in collaboration with the Spanish Incontinence Association.

Healthcare organization, professional roles, referral pathways, access to specialized rehabilitation, and the integration of digital services may differ substantially across regions and healthcare systems. Caution is therefore required when extrapolating the findings to other countries, professional contexts, geographical settings, or models of pelvic floor care.

The transferability and broader applicability of both the DDPP framework and the resulting candidate recommendations require prospective evaluation in other populations and healthcare environments.

### 6.8. Study Strengths

Despite these limitations, the study has several strengths. To our knowledge, it represents the first application of the Delphi-Dialogue Patients–Professionals framework to pelvic floor rehabilitation and integrates patient experiences, professional perspectives, and a subsequent clinician-led feasibility assessment within a sequential study design.

The use of parallel questionnaires, transparent item-level descriptive comparisons, explicit traceability between survey findings and candidate recommendations, and explicit acknowledgment of the exploratory nature of the divergence score provide a structured basis for generating hypotheses and informing future implementation research and patient-centred service development.

## 7. Conclusions

This study identified patient–professional perceptual divergences in selected aspects of pelvic floor rehabilitation, including communication, digital competence, self-monitoring, and reported adherence. Because the study did not assess clinical outcomes, objective care quality, or actual implementation, these differences should be interpreted as exploratory indicators of areas that may warrant further investigation rather than as demonstrated implementation gaps or deficiencies in clinical effectiveness.

By integrating patient and professional perspectives within a structured exploratory framework, the Delphi-Dialogue Patients–Professionals (DDPP) methodology provides an approach for identifying candidate organizational recommendations and assessing their perceived implementation feasibility. The findings identify coordinated rehabilitation pathways, patient education, self-management support, adherence, and digital or hybrid models as potential areas for future implementation research rather than as interventions already demonstrated to improve care.

Beyond pelvic floor rehabilitation, the DDPP framework may offer an exploratory methodology for examining stakeholder perspectives and generating candidate service-development recommendations in rehabilitation and other chronic-care settings. Its scalability, transferability, implementation effects, and capacity to improve organizational or clinical outcomes require prospective evaluation.

## Figures and Tables

**Table 1:** Characteristics of the Real-Time Delphi-based feasibility assessment.

Characteristic	Category
Total participants	37
PM&R physicians	35 Pelvic floor rehabilitation expertise
ASIA Board representatives	2 Patient association representatives
Selection strategy	Purposive selection
Role of the panel	Feasibility assessment of pre-formulated recommendations
Rating format	Six-point Likert scale
Feedback procedure	Real-time anonymized group feedback
Possibility to modify scores	Yes, during the synchronous session

**Table 2:** Characteristics of patient survey respondents.

Characteristic	Category	N	%
Total patient responses		225	100.0
Gender	Female	198	88.0
	Male	27	12.0
	Non-binary	0	0.0
Educational level	University studies	112	49.8
	Secondary education/vocational training	79	35.1
	Primary education	31	13.8
	No formal education	3	1.3
Living situation	Independent private home	211	93.8
	Private home with family caregiver	11	4.9
	Residential facility	3	1.3
	Private home with professional non-family caregiver	0	0.0
	Supported housing	0	0.0
Pelvic floor condition reported (patient can report more than one)	Urinary incontinence	146	64.9
	Fecal incontinence	74	32.9
	Gas incontinence	65	28.9
	Pelvic organ prolapse	41	18.2
	Pelvic pain	39	17.3
Main symptom	Urinary incontinence	113	50.2
	Fecal incontinence	52	23.1
	Pelvic pain	22	9.8
	Pelvic organ prolapse	22	9.8
	Gas incontinence	16	7.1
Time since onset of main symptom	<1 year	42	18.7
	1–5 years	102	45.3
	6–10 years	35	15.6
	11–15 years	19	8.4
	15–20 years	8	3.6
	>20 years	19	8.4
Previously assessed by PM&R for PFD	Yes	181	80.4
	No	44	19.6
Received rehabilitation/physiotherapy treatment for PFD	Yes	161	71.6
	No	64	28.4
Chronic disease in addition to PFD	Yes	92	40.9
	No	133	59.1
Mobility difficulties	Yes	31	13.8
	No	194	86.2

**Table 3:** Characteristics of professional survey respondents.

Characteristic	Category	N	%
Total professional responses		164	100.0
Gender	Female	154	93.9
	Male	10	6.1
	Non-binary	0	0.0
Professional category	PM&R physician	101	61.6
	Physiotherapist	63	38.4
	Nursing	0	0.0
	Other	0	0.0
Workplace	Complex/reference hospital	56	34.1
	Area hospital, approx. 400 beds	51	31.1
	Basic general hospital, approx. 200 beds	26	15.9
	Local hospital, approx. 100 beds	16	9.8
	Other	15	9.1
Experience in pelvic floor dysfunctions	<5 years	65	39.6
	6–10 years	45	27.4
	11–15 years	35	21.3
	16–20 years	11	6.7
	>20 years	8	4.9
Average successive PFD patients/week	<5	41	25.0
	5–10	53	32.3
	11–15	25	15.2
	16–20	22	13.4
	>20	23	14.0
Average new PFD patients/week	<5	96	58.5
	5–10	49	29.9
	11–15	15	9.1
	16–20	4	2.4
	>20	0	0.0
Works in a multidisciplinary pelvic floor unit	Yes	83	50.6
	No	81	49.4
University teaching activity	Yes	33	20.1
	No	131	79.9

**Table 4:** Differences in perception between patients and professionals ordered by divergence.

		Patients			Professionals							
ID	Item	μ	σ	A	μ	σ	A	SDI	ΔA	d	AMD	AHG, pp
Q5	Ability to use Internet in relation to pathology	4.68	1.52	65.0	3.54	0.84	12.2	0.93	4.33	4.02	1.14	52.8
Q2	Information received/provided at the right time	4.19	1.76	51.8	2.95	1.37	14.6	0.79	2.54	1.99	1.24	37.2
Q29	Usefulness of bladder/bowel diary	2.93	1.67	20.3	4.39	1.16	54.3	1.01	1.67	1.69	1.46	34.0
Q7	Fulfilment/adherence to prescribed treatment programs	4.36	1.45	53.1	3.78	1.00	20.7	0.47	1.57	0.74	0.58	32.4
Q28	Difficulty completing bladder/bowel diary	2.64	1.75	18.0	3.93	1.16	31.7	0.87	0.76	0.66	1.29	13.7
Q26	Need for complementary Psychology/Psychiatry treatment	3.07	1.86	27.0	4.30	1.09	46.3	0.81	0.72	0.58	1.23	19.3
Q33	Interest/usefulness of talks and group therapy program	3.73	1.76	36.3	4.68	0.97	67.1	0.67	0.85	0.57	0.95	30.8
Q8	Usefulness of remote communication with rehabilitation team	4.34	1.61	56.6	3.80	1.22	26.8	0.38	1.11	0.42	0.54	29.8
Q10	Sufficiency of phone consultations for home therapy	3.49	1.50	25.7	3.06	1.21	11.6	0.31	1.22	0.38	0.43	14.1
Q34	Usefulness of previous group therapy	3.30	2.08	40.0	4.57	1.10	58.3	0.77	0.46	0.35	1.27	18.3
Q20	Impact of pathology on social life	4.63	1.48	62.8	5.47	0.71	92.7	0.72	0.48	0.34	0.84	29.9
Q21	Impact of pathology on quality of life	4.65	1.44	61.9	5.43	0.74	91.5	0.68	0.48	0.33	0.78	29.6
Q11	Sufficiency of video consultations for home therapy	3.69	1.53	33.2	3.27	1.18	16.5	0.31	1.01	0.31	0.42	16.7
Q19	Agreement on therapeutic goals	4.24	1.61	51.2	4.76	1.02	65.9	0.38	0.29	0.11	0.52	14.7
Q18	Resolution of doubts/needs via remote consultation	3.44	1.53	27.9	3.30	1.38	18.9	0.10	0.48	0.05	0.14	9.0
Q9	Need for occasional face-to-face visits to follow home therapy	3.78	1.70	37.6	4.30	1.26	43.3	0.35	0.15	0.05	0.52	5.7
Q14	Motivation for virtual therapy with other patients	3.35	1.77	31.4	3.55	1.31	23.8	0.12	0.32	0.04	0.20	7.6
Q31	Written instructions help perform home exercises	4.24	1.60	52.2	4.68	0.98	59.1	0.33	0.13	0.04	0.44	6.9
Q30	Adherence to pharmacological treatment	3.86	2.08	47.0	3.95	1.04	31.1	0.05	0.51	0.03	0.09	15.9
Q1	Sufficient information about pelvic floor pathology	4.75	1.53	69.0	4.60	0.98	59.8	0.12	0.15	0.02	0.15	9.2
Q22	Satisfaction with consultation time	4.62	1.56	64.0	4.45	1.26	55.5	0.12	0.15	0.02	0.17	8.5
Q25	Social/psychological impact considered in assessment	4.79	1.61	70.1	5.05	1.00	76.8	0.20	0.10	0.02	0.26	6.7
Q4	Face-to-face reviews meet patient expectations	4.64	1.66	67.8	4.53	0.94	58.5	0.08	0.16	0.01	0.11	9.3
Q23	Follow-up visits help continue home exercises	4.57	1.56	61.5	4.45	1.15	55.6	0.08	0.11	0.01	0.12	5.9
Q36	Mobile apps recommended by professionals help perform home exercises	4.34	1.58	54.0	4.52	1.02	59.1	0.14	0.09	0.01	0.18	5.1
Q15	Usefulness of requesting telehealth-based consultations	3.74	1.67	36.3	3.90	1.38	39.0	0.11	0.07	0.01	0.16	2.7
Q3	Rehabilitation team involvement in care	4.85	1.63	74.4	5.05	0.98	78.0	0.15	0.05	0.01	0.20	3.6
Q13	Usefulness of sharing experiences virtually with other patients	3.52	1.75	32.7	3.92	1.25	33.5	0.26	0.02	0.01	0.40	0.8
Q27	Ease of access to pelvic floor rehabilitation unit	3.64	1.85	38.1	3.63	1.41	33.5	0.00	0.14	0.00	0.01	4.6
Q6	Usefulness of prescribed home exercise programs	4.64	1.47	61.9	4.63	1.19	55.5	0.01	0.12	0.00	0.01	6.4
Q16	Telehealth-based visit can substitute some face-to-face visits	3.01	1.62	20.4	3.07	1.45	18.3	0.04	0.11	0.00	0.06	2.1
Q17	Usefulness/need of preparatory instructions before remote consultation	4.04	1.54	42.5	4.13	1.31	44.5	0.06	0.05	0.00	0.09	2.0
Q24	App for home training upload and professional follow-up	4.69	1.59	67.3	4.76	1.17	64.0	0.04	0.05	0.00	0.07	3.3
Q32	Video exercises help perform home exercises	4.64	1.46	62.4	4.73	0.95	64.6	0.07	0.04	0.00	0.09	2.2
Q35	Usefulness of complementary devices for home exercises	4.64	1.39	69.2	4.79	0.97	67.3	0.12	0.03	0.00	0.15	1.9
Q12	Hybrid in-person/remote program would cover needs	4.43	1.53	56.2	4.50	1.15	56.7	0.05	0.01	0.00	0.07	0.5

AMD = absolute difference between patient and professional mean ratings, |μ_pat − μ_pro|. AHG = absolute difference between patient and professional high-agreement proportions, |A_pat − A_pro|, expressed in percentage points. AMD and AHG were included as descriptive sensitivity measures and did not replace the original divergence score.

**Table 5:** Recommendations to innovate pelvic floor rehabilitation care delivery.

No.	Candidate Recommendation	L.S.I. *	Main Phase 1 Finding	Mean d **	Feasibility μ	σ	Med	IQR
1	Implement a structured home exercise program	Q6, Q7, Q19, Q31, Q32	Divergence in reported adherence, with broad alignment regarding the usefulness of home exercises, shared therapeutic goals, and written or video-based support.	0.178	5.4	0.8	6	1
2	Integrate adherence-monitoring tools into pelvic floor rehabilitation	Q7, Q28, Q29, Q30	Divergence in reported adherence and in the perceived difficulty and usefulness of bladder and bowel diaries, with limited divergence in pharmacological adherence.	0.780	5.2	0.9	5	1
3	Increase the visibility of pelvic floor rehabilitation pathways in PM&R consultations	Q1, Q2, Q3, Q27	Marked divergence in the timing of information delivery, with lower divergence in information sufficiency, rehabilitation-team involvement, and access to specialized services.	0.505	5.0	1.0	5	1
4	Develop a digital application to monitor pelvic floor exercise programs	Q5, Q7, Q24, Q35, Q36	Marked divergence in digital competence and reported adherence, with broad alignment regarding app-supported follow-up, complementary devices, and mobile support for home exercises.	0.954	4.7	1.1	5	2
5	Promote digital health literacy tailored to pelvic floor patients	Q5, Q8, Q17, Q18	Marked divergence in digital competence and moderate divergence in the usefulness of remote communication, with lower divergence in preparatory support and resolution of needs remotely.	1.123	4.1	1.4	4	3
6	Implement hybrid follow-up models—in-person and remote—for pelvic floor care	Q9, Q10, Q11, Q12, Q15, Q16, Q18, Q23	Generally low divergence regarding hybrid care, telehealth, remote follow-up, and the role of occasional face-to-face visits, although telephone and video consultation sufficiency showed greater differences.	0.101	4.3	1.3	4	2
7	Develop group-based and preventive pelvic floor health programs with graduated difficulty levels	Q13, Q14, Q20, Q21, Q26, Q33, Q34	Moderate divergence regarding psychosocial support, pathology impact, and the usefulness of group-based interventions, with lower divergence in virtual peer interaction.	0.317	3.6	1.5	4	3
8	Standardize educational materials for pelvic floor rehabilitation	Q1, Q2, Q17, Q31, Q32	Marked divergence in information timing, with broad alignment regarding information sufficiency, preparatory instructions, and written and video-based exercise materials.	0.410	4.6	1.2	5	2
9	Reinforce multidisciplinary coordination in pelvic floor rehabilitation	Q3, Q19, Q25, Q26	Moderate divergence in the perceived need for psychological or psychiatric support, with lower divergence in professional involvement, shared goals, and consideration of psychosocial impact.	0.180	4.0	1.2	4	2
10	Improve global accessibility and patient satisfaction with pelvic floor services	Q4, Q22, Q23, Q27	Low patient–professional divergence regarding access, consultation time, face-to-face reviews, and follow-up, although the corresponding ratings indicate areas that may still warrant organizational attention.	0.010	3.4	1.6	3	3

* L.S.I. = linked survey items. ** Mean d = arithmetic mean of the divergence scores corresponding to the survey items linked to each recommendation.

## Data Availability

The complete patient and professional questionnaires are provided as Supplementary Files S1 and S2. Anonymized aggregate item-level data supporting the findings of this study are available from the corresponding author upon reasonable request. Individual-level response data are not publicly available because of privacy and data-protection considerations.

## Supplementary Materials

The following supporting information can be downloaded at: https://www.mdpi.com/article/10.3390/healthcare14162520/s1, Supplementary File S1, Patient questionnaire; Supplementary File S2, Healthcare professional questionnaire.

## Abbreviations

SERMEF	Spanish Society of Physical Medicine and Rehabilitation
ASIA	Spanish Incontinence Association
PFD	Pelvic floor dysfunctions
PM&R	Physical Medicine and Rehabilitation
DDPP	Delphi-Dialogue Patients–Professionals
SDI	Standardized Difference Index
ΔA	Difference in high-agreement proportions
IQR	Interquartile range
GDPR	General Data Protection Regulation
CEIm	Research Ethics Committee

## References

[1] Abrams P., Cardozo L., Wagg A., Wein A. (2017). Incontinence.

[2] Milsom I., Gyhagen M. (2019). The prevalence of urinary incontinence. Climacteric.

[3] Kenton K., Mueller E.R. (2006). The gl.obal burden of female pelvic floor disorders. BJU Int..

[4] European Association of Urology (EAU) (2020). EAU Guidelines on Urinary Incontinence 2020.

[5] American Urological Association (AUA), Society of Urodynamics, Female Pelvic Medicine & Urogenital Reconstruction (SUFU) (2024). Diagnosis and Treatment of Idiopathic Overactive Bladder in Adults: AUA/SUFU Guideline.

[6] Dumoulin C., Cacciari L.P., Hay-Smith E.J.C. (2018). Pelvic floor muscle training versus no treatment, or inactive control treatments, for urinary incontinence in women. Cochrane Database Syst. Rev..

[7] Rose A., Rosewilliam S., Soundy A. (2017). Shared decision making within goal setting in rehabilitation settings: A systematic review. Patient Educ. Couns..

[8] Vahdat S., Hamzehgardeshi L., Hessam S., Hamzehgardeshi Z. (2014). Patient involvement in health care decision making: A review. Iran. Red Crescent Med. J..

[9] Sagen J.S., Kjeken I., Habberstad A., Linge A.D., Simonsen A.E., Lyken A.D., Irgens E.L., Framstad H., Lyby P.S., Klokkerud M. (2024). Patient involvement in the rehabilitation process is associated with improvement in function and goal attainment: Results from an explorative longitudinal study. J. Clin. Med..

[10] National Institute for Health and Care Excellence (2021). Pelvic Floor Dysfunction: Prevention and Non-Surgical Management (NG210).

[11] Hodyl N., Mason G., Ribbons K., Bailey L., O’Malley A., Ward T., Ward S., Pollack M., Nilsson M., Walker F.R. (2024). Barriers and enablers for accessing rehabilitation services: Findings from the Rehabilitation Choices Study, Part 1—Healthcare professionals’ perspectives. Health Expect..

[12] Patel A., Gellhorn A., Neerukonda K., Kwasnica C. (2024). Multidisciplinary collaborative consensus statement on the barriers and solutions to care access, patient and caregiver support, and clinical capacity and capability for patients with spasticity. PM&R.

[13] Bascuñana-Ambrós H., Formigo-Couceiro J., Climent-Barberá J.M., Guirao-Cano L., Catta-Preta M., Trejo-Omeñaca A., Monguet-Fierro J.M. (2026). Aligning minds in spasticity care—A two-phase Delphi-dialogue study of patients and professionals in Spain. Toxins.

[14] Lee A.C., Deutsch J.E., Holdsworth L., Kaplan S.L., Kosakowski H., Latz R., McNeary L.L., O’Neil J., Ronzio O., Sanders K. (2024). Telerehabilitation in physical therapist practice: A clinical practice guideline from the American Physical Therapy Association. Phys. Ther..

[15] Hao J., Yao Z., Remis A., Huang B., Li Y., Yu X. (2024). Pelvic floor muscle training in telerehabilitation: A systematic review and meta-analysis. Arch. Gynecol. Obstet..

[16] European Parliament, Council of the European Union (2016). Regulation (EU) 2016/679 (General Data Protection Regulation). Off. J. Eur. Union.

[17] Alkhuzaimi F., Rainey D., Brown Wilson C., Bloomfield J. (2025). The impact of mobile health interventions on service users’ health outcomes and the role of health professions: A systematic review of systematic reviews. BMC Digit. Health.

[18] Marcolino M.S., Oliveira J.A.Q., D’Agostino M., Ribeiro A.L., Alkmim M.B.M., Novillo-Ortiz D. (2018). The impact of mHealth interventions: Systematic review of systematic reviews. JMIR mHealth uHealth.

[19] Lang S., McLelland C., MacDonald D., Hamilton D.F. (2022). Do digital interventions increase adherence to home exercise rehabilitation? A systematic review of randomized controlled trials. Arch. Physiother..

[20] Lemmo D., Bianco R., Mezza F., De Luca V., Illario M., Iaccarino G., Freda M.F. (2025). Between regulatory functions and emotional burden: Balancing engagement in digital health interventions for self-care in chronic illness. Front. Psychol..

[21] Nilsen P., Albers B., Shlonsky A., Mildon R. (2020). Making sense of implementation theories, models, and frameworks. Implementation Science 3.0.

[22] Teisberg E., Wallace S., O’Hara S. (2020). Defining and implementing value-based health care: A strategic framework. Acad. Med..

