# Supplementary material for: Translating Patient–Professional Divergence into Care-Improvement Priorities in Pelvic Floor Rehabilitation: A Delphi-Dialogue Study
Source: Healthcare (Basel). 2026 Aug 12;14(16):2520. doi: 10.3390/healthcare14162520 (PMC13512020; doi:10.3390/healthcare14162520)

DDPP - Suelo Pelvico - Profesionales

164 respuestas

¿Con qué género te identificas?

164 de 164 personas han respondido

Mujer 154 res 93.9%

Hombre 10 res 6.1%

No Binario 0 res 0%

¿A qué categoria profesional perteneces?

164 de 164 personas han respondido

Medico Rehabilitador 101 res 61.6%

Fisioterapeuta 63 res 38.4%

Enfermería 0 res 0%

Other 0 res 0%

¿Dónde trabajas?

164 de 164 personas han respondido

Hospital complejo y de referencia 56 res 34.1%

Hospital de área (aprox. 400 camas) 51 res 31.1%

Hospital general básico (aprox. 200 camas) 26 res 15.9%

Hospital comarcal (aprox. 100 camas) 16 res 9.8%

Other 15 res 9.1%

¿En qué CCAA trabajas?

164 de 164 personas han respondido

Comunidad de Madrid 22 res 13.4%

Canarias 19 res 11.6%

Galicia 15 res 9.1%

Comunitat Valenciana	10 res	6.1%
Castilla-La Mancha	8 res	4.9%
Illes Balears	8 res	4.9%
Principado de Asturias	7 res	4.3%
La Rioja	1 res	0.6%
Andalucía	0 res	0%
Aragón	0 res	0%
Cantabria	0 res	0%
Castilla y León	0 res	0%
Cataluña	0 res	0%
Ciudad Autónoma de Ceuta	0 res	0%
Ciudad Autónoma de Melilla	0 res	0%
Comunidad Foral de Navarra	0 res	0%
Extremadura	0 res	0%
País Vasco	0 res	0%

Región de Murcia 0 res 0%

¿Cuántos años de experiencia tiene en el ámbito de las disfunciones del suelo pélvico?

164 de 164 personas han respondido

menos de 5 años 65 res 39.6%

entre 6 y 10 años 45 res 27.4%

entre 11 y 15 años 35 res 21.3%

entre 16 y 20 años 11 res 6.7%

más de 20 años 8 res 4.9%

¿Cuántos pacientes sucesivos con patología de suelo pélvico ves en promedio a la semana?

164 de 164 personas han respondido

entre 5 y 10 53 res 32.3%

menos de 5 41 res 25%

entre 11 y 15 25 res 15.2%

más de 20 — 23 res — 14%

entre 16 y 20 — 22 res — 13.4%

¿Cuántos pacientes nuevos con patología de suelo pélvico ves en promedio a la semana?

164 de 164 personas han respondido

menos de 5 — 96 res — 58.5%

entre 5 y 10 — 49 res — 29.9%

entre 11 y 15 — 15 res — 9.1%

entre 16 y 20 — 4 res — 2.4%

más de 20 — 0 res — 0%

¿Cuál es tu actividad investigadora global, medida en publicaciones científicas?

164 de 164 personas han respondido

ninguna — 106 res — 64.6%

menos de 5 — 50 res — 30.5%

entre 5 y 30 — 8 res — 4.9%

más de 30 — 0 res — 0%

¿Realizas actividad docente universitaria?

164 de 164 personas han respondido

Yes — 33 res — 20.1%

No — 131 res — 79.9%

¿Crees que proporcionas a tus pacientes suficiente información sobre su patología de de suelo pélvico?

164 de 164 personas han respondido

4.6 Puntuación media

1	2	3	4	5	6
1.2%	1.8%	7.9%	29.3%	44.5%	15.2%
2 res	3 res	13 res	48 res	73 res	25 res

Poco Mucho

¿Crees que tus pacientes han recibido información sobre su patología de suelo pélvico en el momento adecuado?

164 de 164 personas han respondido

3.0 Puntuación media

17.1%	22.6%	26.8%	18.9%	11%	3.7%
28 res	37 res	44 res	31 res	18 res	6 res

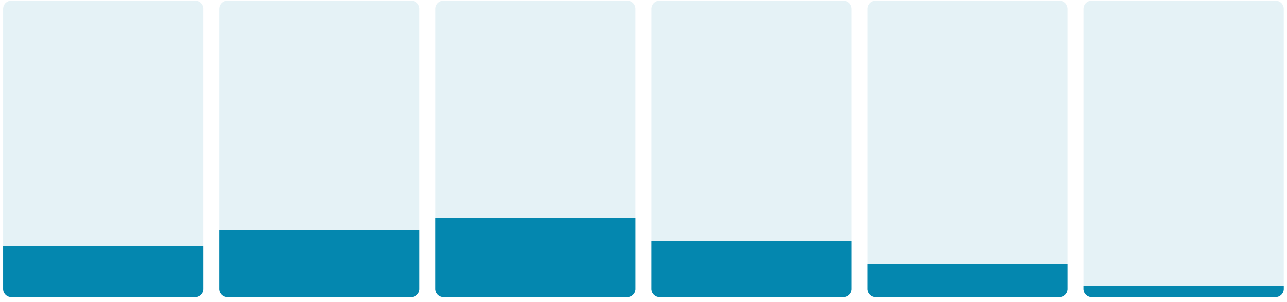

1	2	3	4	5	6

Poco Mucho

¿Crees que tu formación ha sido adecuada en el ámbito de suelo pélvico?

164 de 164 personas han respondido

4.1 Puntuación media

2.4%	5.5%	20.1%	33.5%	30.5%	7.9%
4 res	9 res	33 res	55 res	50 res	13 res

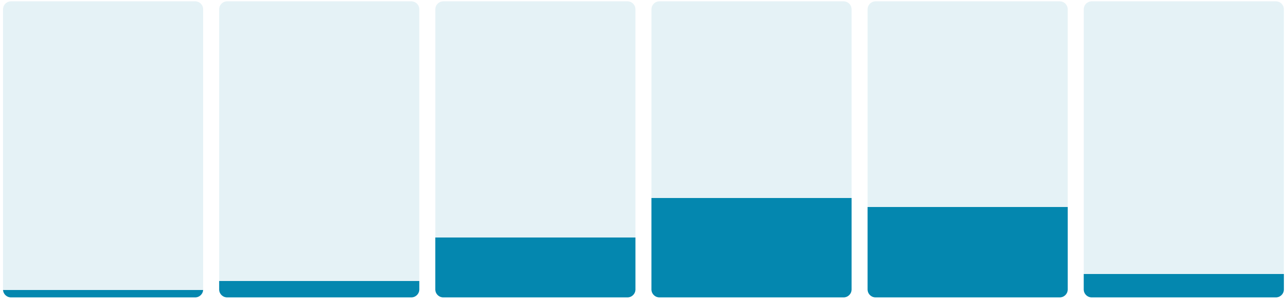

1	2	3	4	5	6
Poco					Mucho

¿Se deriva a tu unidad de suelo pélvico desde otros servicios distintos a los de Urología y/o Ginecología?

164 de 164 personas han respondido

Yes	130 res	79.3%
No	34 res	20.7%

¿Desde qué servicio se deriva a tu unidad de rehabilitación de suelo pélvico?

130 de 164 personas han respondido

Ginecología	120 res	92.3%
Urología	120 res	92.3%
Coloproctología	108 res	83.1%
Digestivo	81 res	62.3%
Atención Primaria	66 res	50.8%
MFRHB	66 res	50.8%
Oncología Médica	29 res	22.3%

Oncología Radioterápica 29 res 22.3%

Pediatría 28 res 21.5%

Neurocirugía/Neurología 23 res 17.7%

Prevención de RRLL 7 res 5.4%

Other 16 res 12.3%

¿Trabajas dentro de una unidad multidisciplinar de suelo pélvico?

164 de 164 personas han respondido

Yes 83 res 50.6%

No 81 res 49.4%

¿Quiénes constituyen la unidad multidisciplinar de suelo pélvico?

83 de 164 personas han respondido

Ginecología	71 res	85.5%
MFRHB	70 res	84.3%
Urología	67 res	80.7%
Coloproctología	60 res	72.3%
Digestivo	35 res	42.2%
Neurocirugía/Neurología	7 res	8.4%
Pediatría	7 res	8.4%
Oncología Médica	2 res	2.4%
Oncología Radioterápica	1 res	1.2%
Other	34 res	41%

¿Tu unidad realiza sesiones periódicas multidisciplinares?

83 de 164 personas han respondido

Mensuales	43 res	51.8%
No	18 res	21.7%

A demanda 15 res 18.1%

Quincenales 6 res 7.2%

Semanales 1 res 1.2%

¿Qué pruebas diagnósticas realizas en la consulta de rehabilitación de suelo pélvico?

164 de 164 personas han respondido

EMG-BFB 91 res 55.5%

Eco transperineal 43 res 26.2%

Perineometría 29 res 17.7%

Manometría 22 res 13.4%

Urodinamia 10 res 6.1%

Flujometría 3 res 1.8%

Other 35 res 21.3%

¿Realizas tratamientos intervencionistas en la unidad de rehabilitación de suelo pélvico?

164 de 164 personas han respondido

No 86 res 52.4%

Neuromodulación percutánea 49 res 29.9%

Infiltración de cicatrices 35 res 21.3%

Infiltración MSP 35 res 21.3%

Punción seca 28 res 17.1%

Bloqueos nerviosos 19 res 11.6%

¿Por qué no realizas tratamientos intervencionistas?

86 de 164 personas han respondido

Las dos anteriores 42 res 48.8%

No tengo formación adecuada 23 res 26.7%

No tengo los medios adecuados 18 res 20.9%

Ninguna de las anteriores 3 res 3.5%

¿En qué medida consideras que tu equipo de MFRHB se implica con la atención de los pacientes?

164 de 164 personas han respondido

5.0 Puntuación media

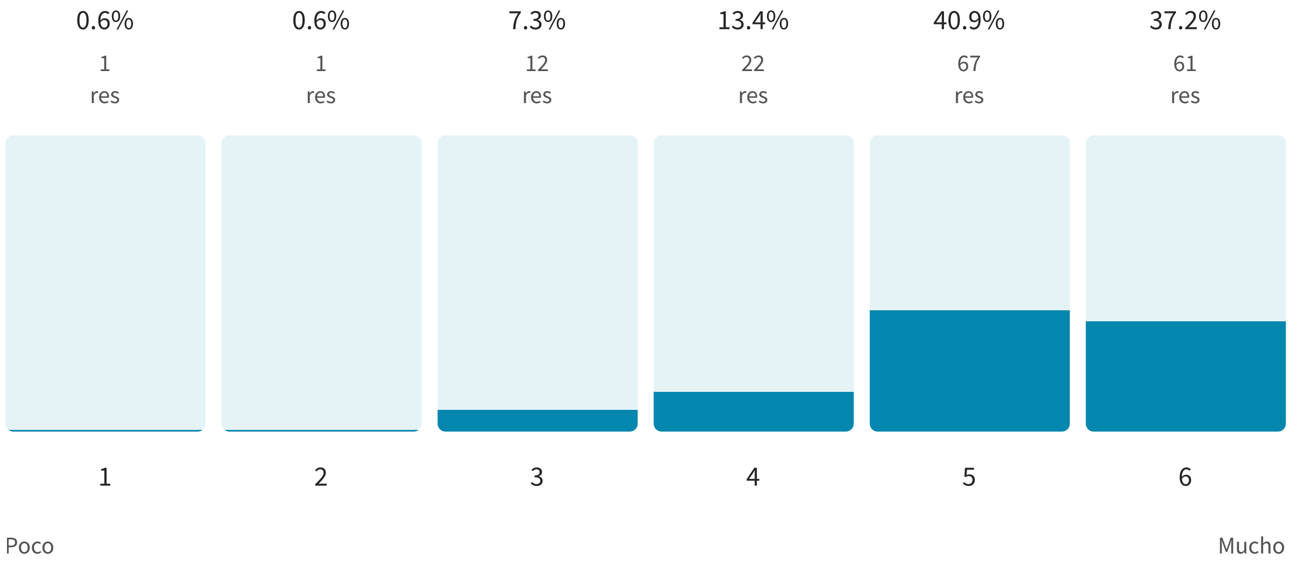

¿En qué medida las revisiones presenciales en consulta de rehabilitación crees que cumplen con las expectativas del paciente?

164 de 164 personas han respondido

4.5 Puntuación media

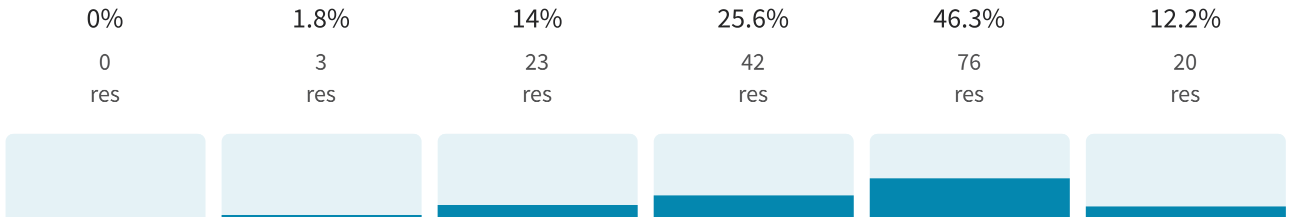

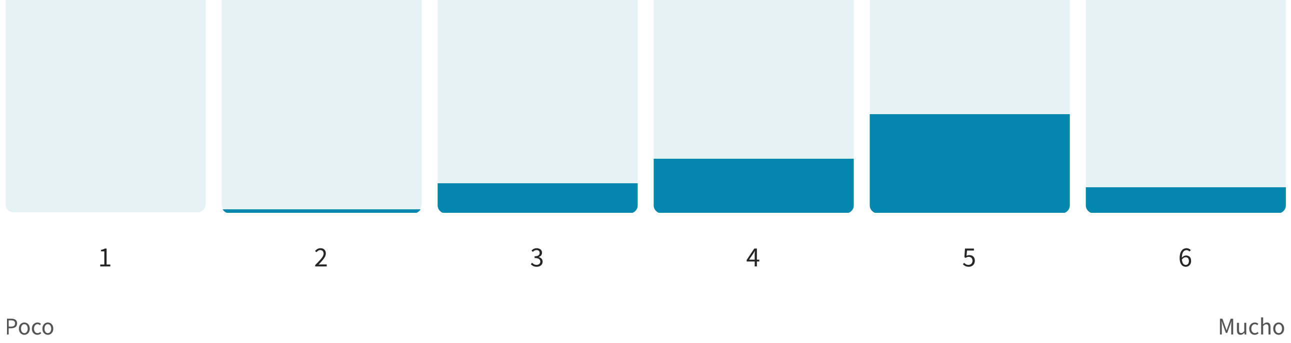

¿En qué medida consideras que tus pacientes estan capacitados para usar Internet en relación a su patología?

164 de 164 personas han respondido

3.5 Puntuación media

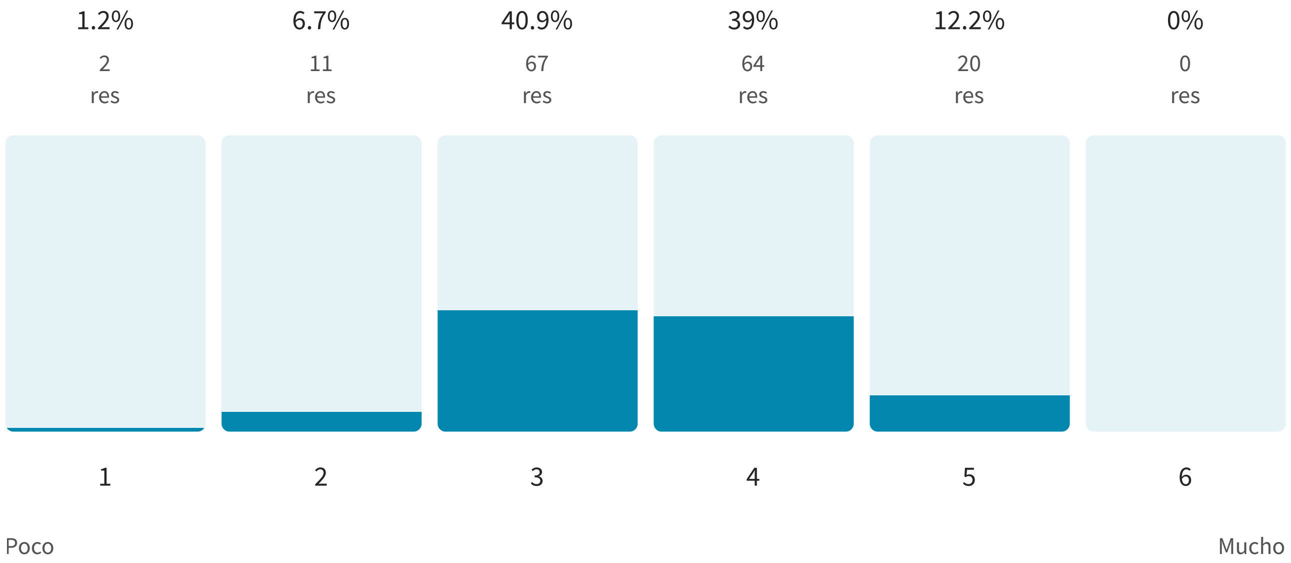

¿En qué medida consideras útil la prescripción de programas de tratamiento para que el paciente haga ejercicios en su casa?

164 de 164 personas han respondido

4.6 Puntuación media

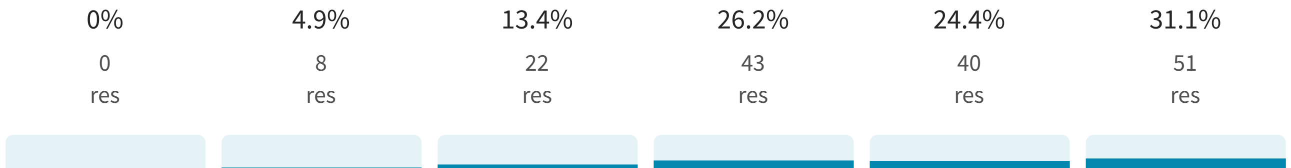

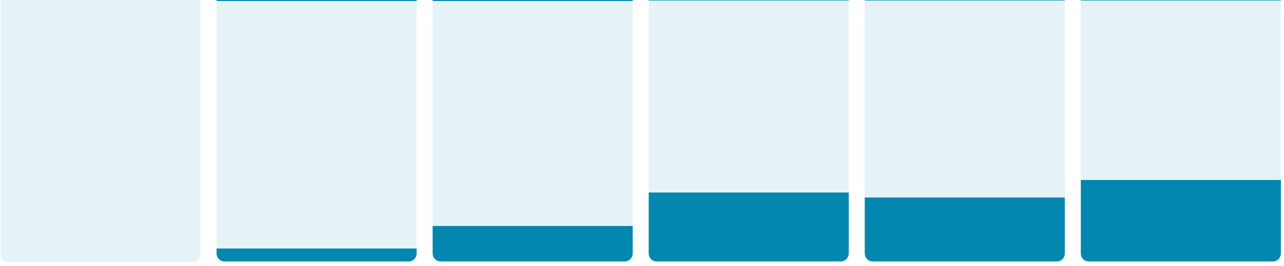

1 2 3 4 5 6

Poco Mucho

¿En general, en qué grado se cumplen por parte de tus pacientes los programas de tratamiento pautados?

164 de 164 personas han respondido

3.8 Puntuación media

1	2	3	4	5	6
1.2%	7.9%	27.4%	42.7%	16.5%	4.3%
2 res	13 res	45 res	70 res	27 res	7 res

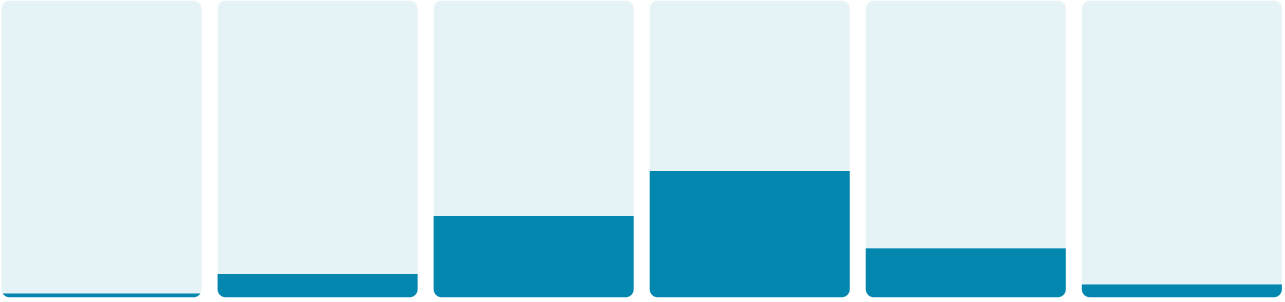

1 2 3 4 5 6

Poco Mucho

¿En general, cuál crees que es la principal dificultad que encuentran para cumplirlos?

164 de 164 personas han respondido

No tienen tiempo 96 res 58.5%

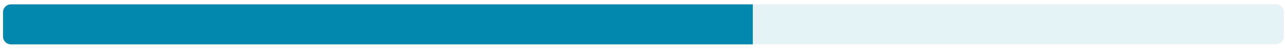

No se acuerdan 49 res 29.9%

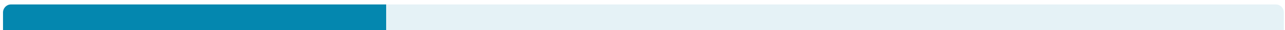

No tienen dificultad 17 res 10.4%

Son difíciles 2 res 1.2%

No los han aprendido 0 res 0%

¿En qué medida consideras útil comunicarte a distancia con tus pacientes?

164 de 164 personas han respondido

3.8 Puntuación media

1	2	3	4	5	6
3.7%	11%	22%	36.6%	18.3%	8.5%
6 res	18 res	36 res	60 res	30 res	14 res

Poco — Mucho

¿Cuál de los siguientes formatos prefieres para prescribir la terapia a tus pacientes?

164 de 164 personas han respondido

Instrucciones escritas 112 res 68.3%

Video modelo 26 res 15.9%

App 13 res 7.9%

Other 13 res 7.9%

¿Cuál de los siguientes canales utilizas para comunicarte con tus pacientes?

164 de 164 personas han respondido

Teléfono 118 res 72%

Correo electrónico 16 res 9.8%

Whatsapp 6 res 3.7%

Plataforma propia 3 res 1.8%

App 2 res 1.2%

Facebook/ Redes Sociales 0 res 0%

Videollamada 0 res 0%

Other 19 res 11.6%

¿De cuáles de las siguientes opciones dispones para comunicarte con tu paciente?

164 de 164 personas han respondido

Teléfono 147 res 89.6%

Correo electrónico 60 res 36.6%

Videollamada 12 res 7.3%

Whatsapp 11 res 6.7%

Facebook/ Redes Sociales 5 res 3%

Plataforma propia 4 res 2.4%

App 3 res 1.8%

Other 10 res 6.1%

¿En qué medida consideras que el paciente necesita citas presenciales ocasionales con el equipo de MFRHB para seguir la terapia en casa?

164 de 164 personas han respondido

4.3 Puntuación media

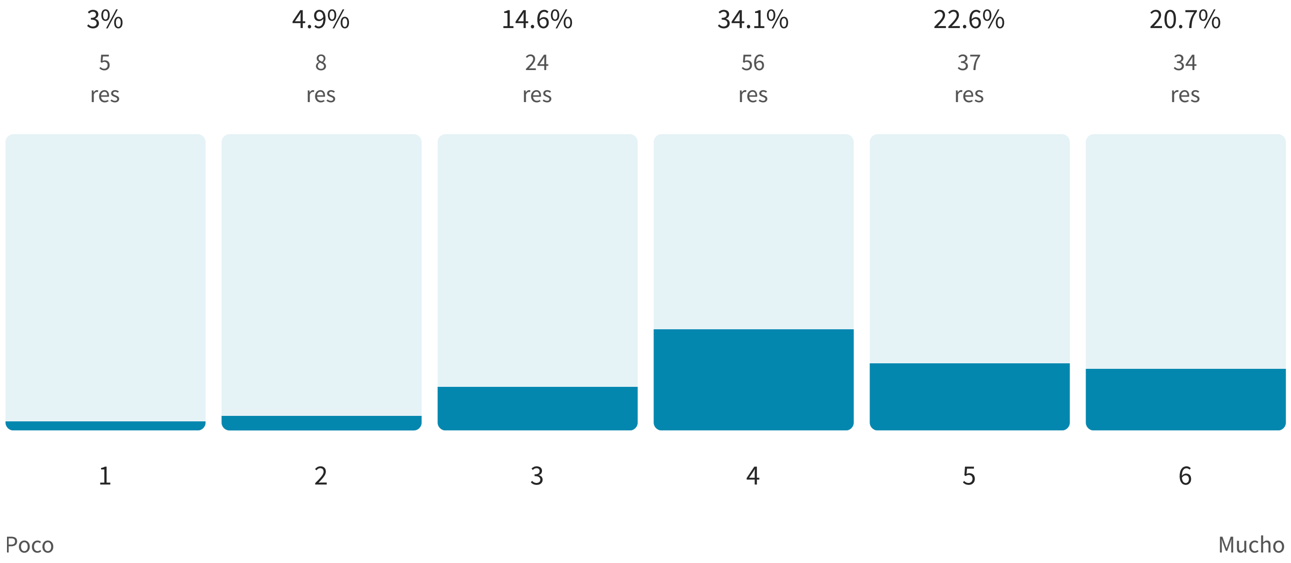

¿En qué medida crees que al paciente le sería suficiente las citas telefónicas con equipo MFRHB para seguir la terapia en casa?

164 de 164 personas han respondido

3.1 Puntuación media

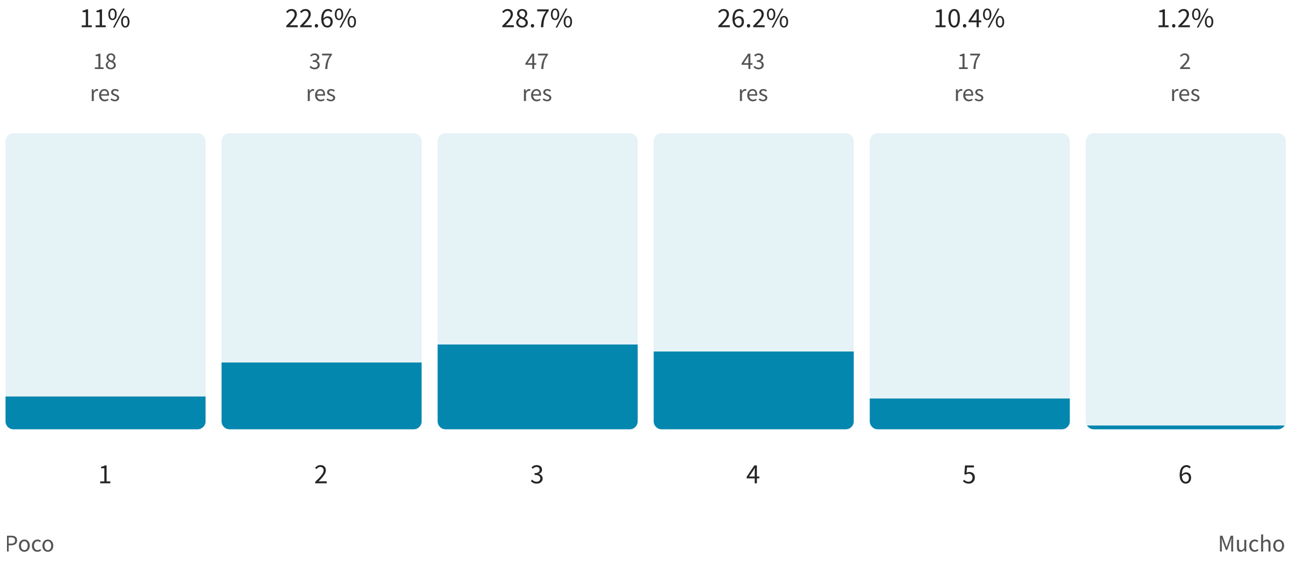

¿En qué medida crees que al paciente le sería suficiente las videollamadas con equipo MFRHB para seguir la terapia en casa?

164 de 164 personas han respondido

3.3 Puntuación media

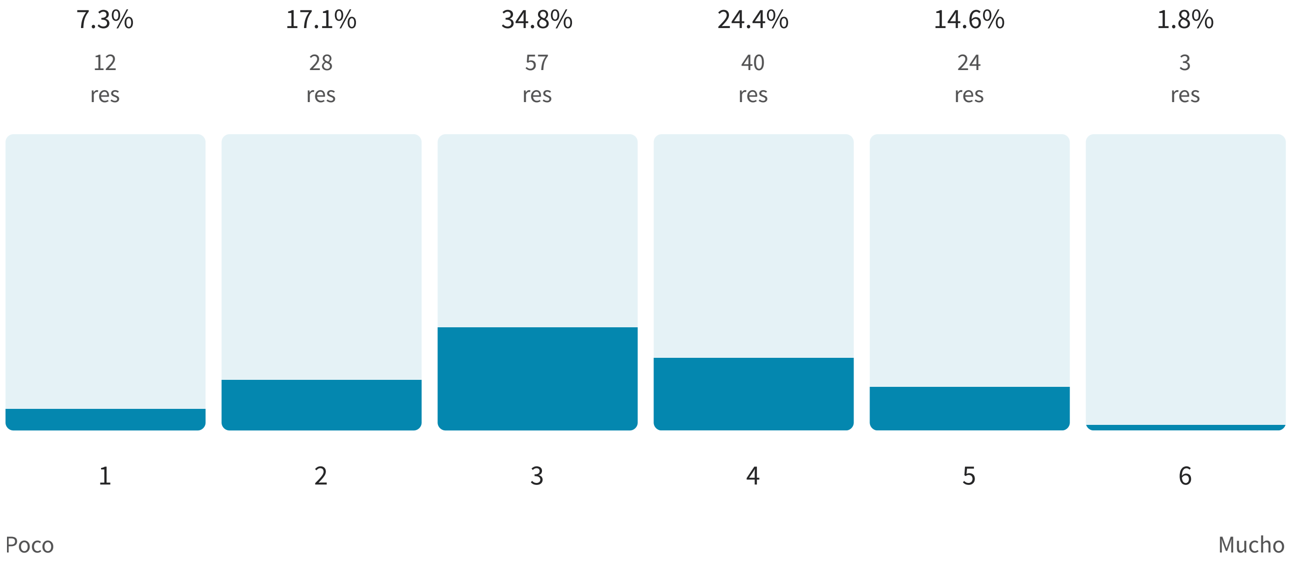

¿En qué medida un programa mixto de tratamiento presencial y no-presencial cubriría las necesidades de tus pacientes?

164 de 164 personas han respondido

4.5 Puntuación media

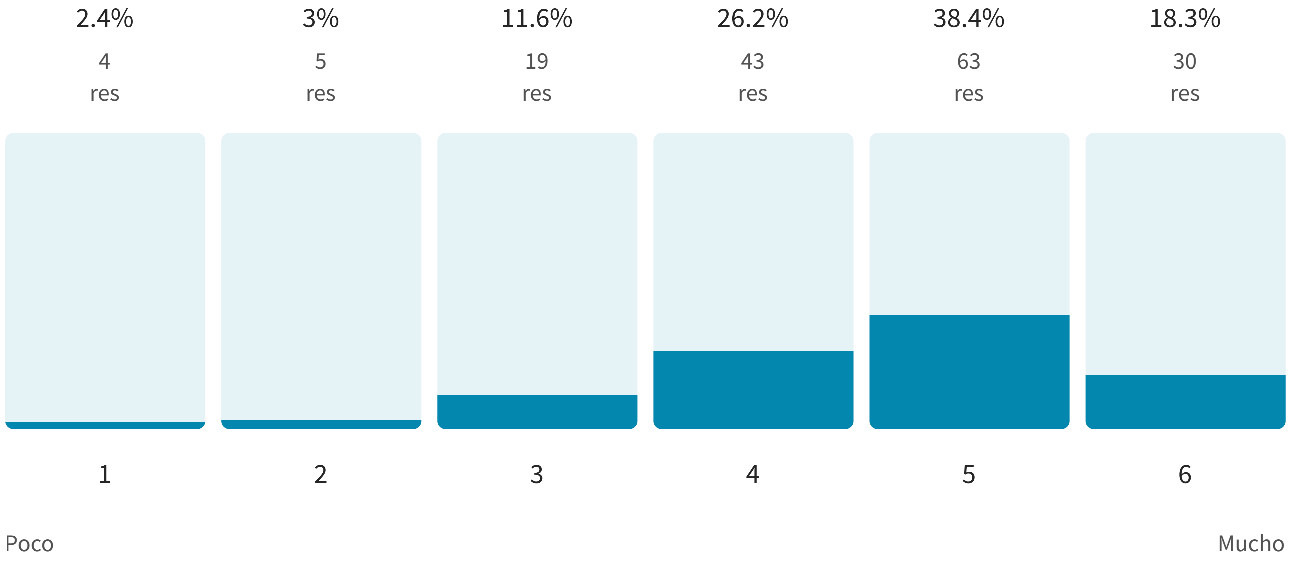

¿En qué medida consideras de utilidad que tus pacientes compartan sus experiencias con otros pacientes de forma virtual?

164 de 164 personas han respondido

3.9 Puntuación media

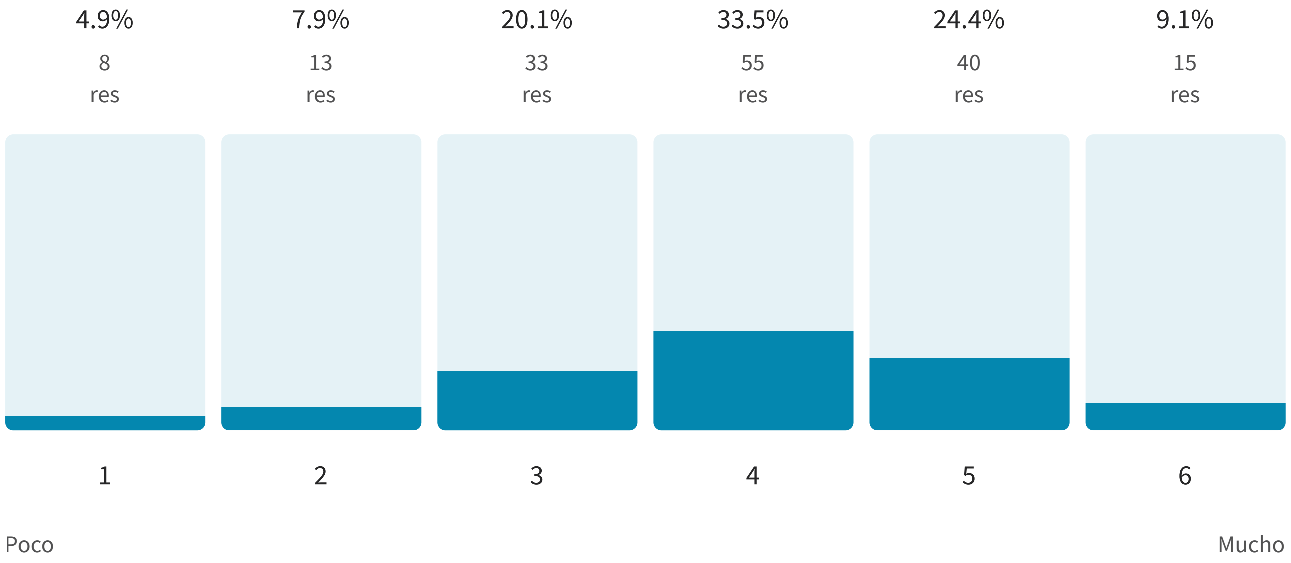

¿En qué medida consideras que a tus pacientes les motivaría hacer terapia de forma virtual con otros pacientes?

164 de 164 personas han respondido

3.5 Puntuación media

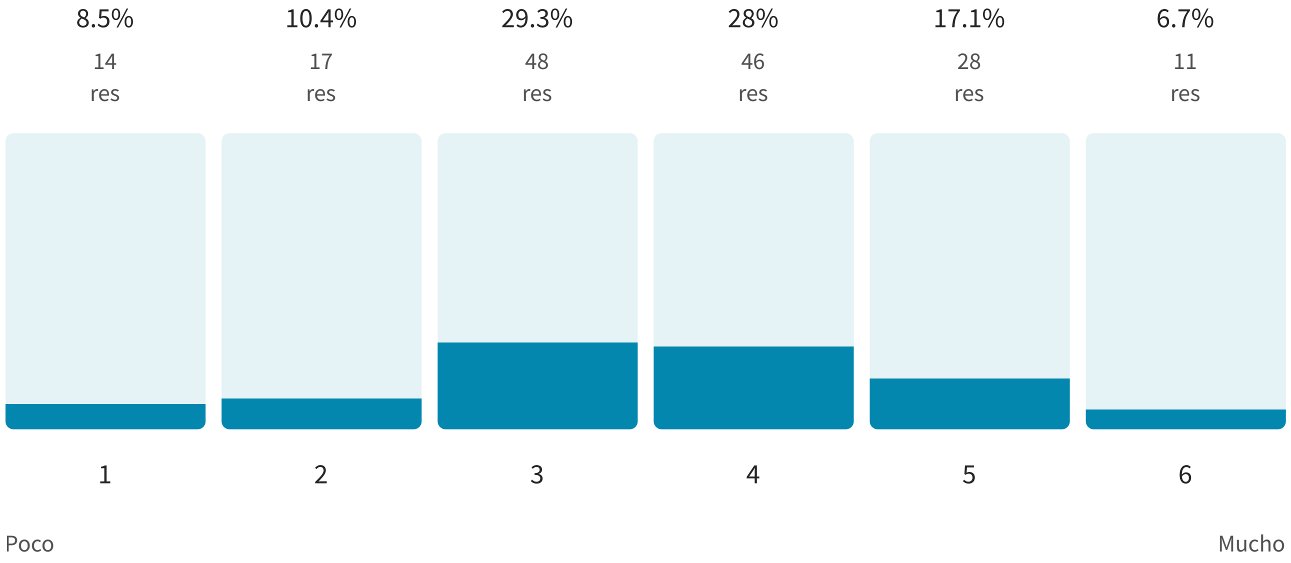

¿En qué medida consideras útil que tus pacientes puedan solicitar consultas no presenciales?

164 de 164 personas han respondido

3.9 Puntuación media

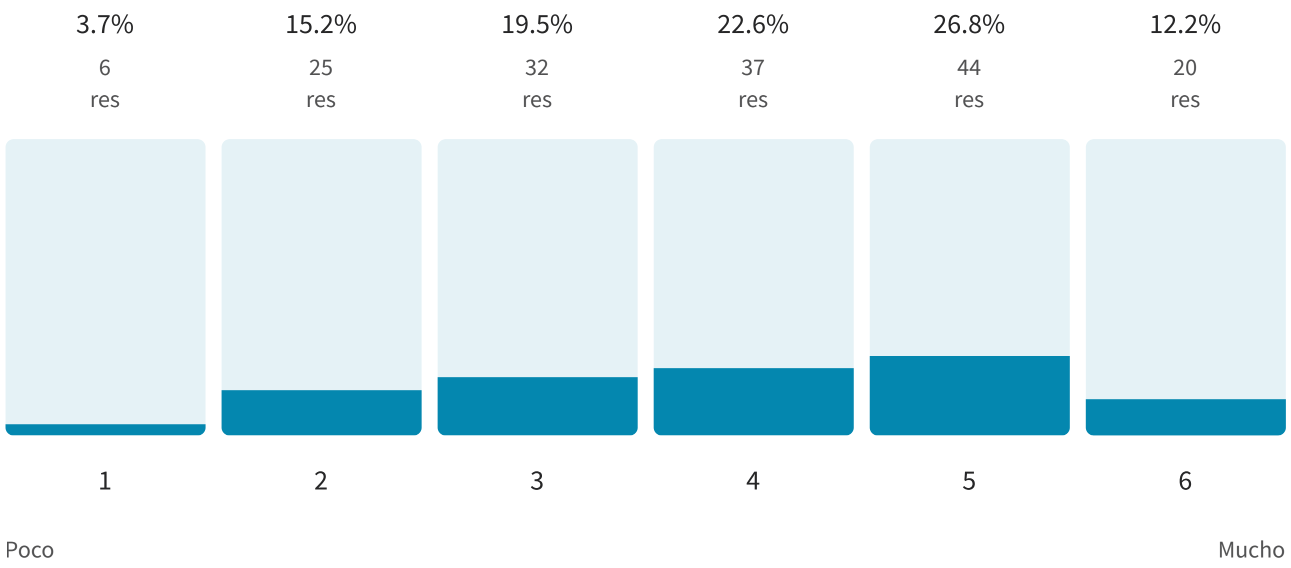

¿En qué medida consideras que la visita no presencial sustituye a alguna de las visitas convencionales presenciales?

164 de 164 personas han respondido

3.1 Puntuación media

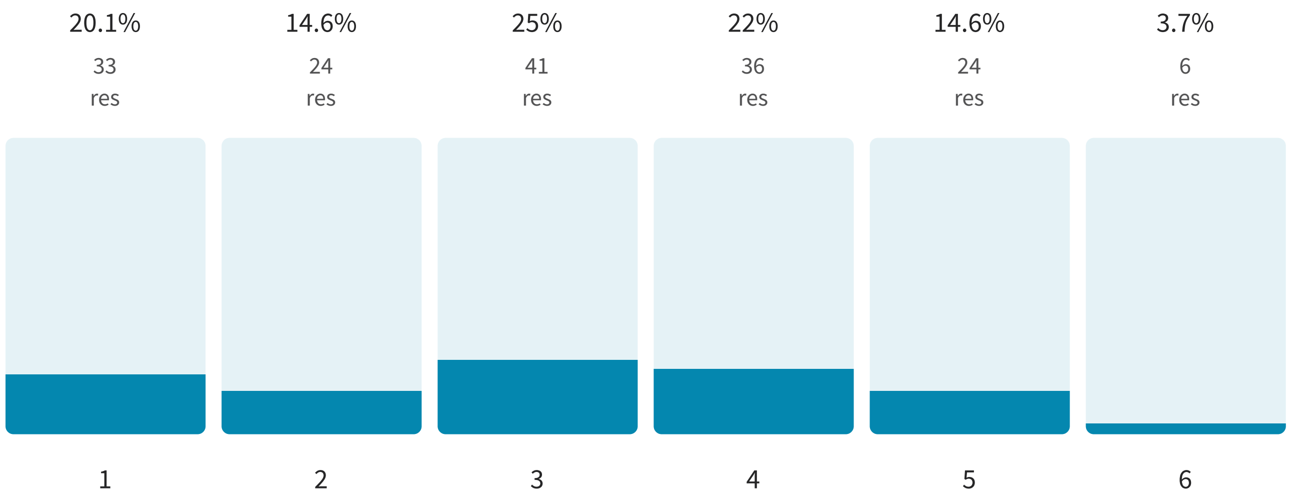

Poco Mucho

¿En qué medida consideras necesario disponer de tutoriales previos para preparar la consulta/tratamiento no presencial de tus pacientes?

164 de 164 personas han respondido

4.1 Puntuación media

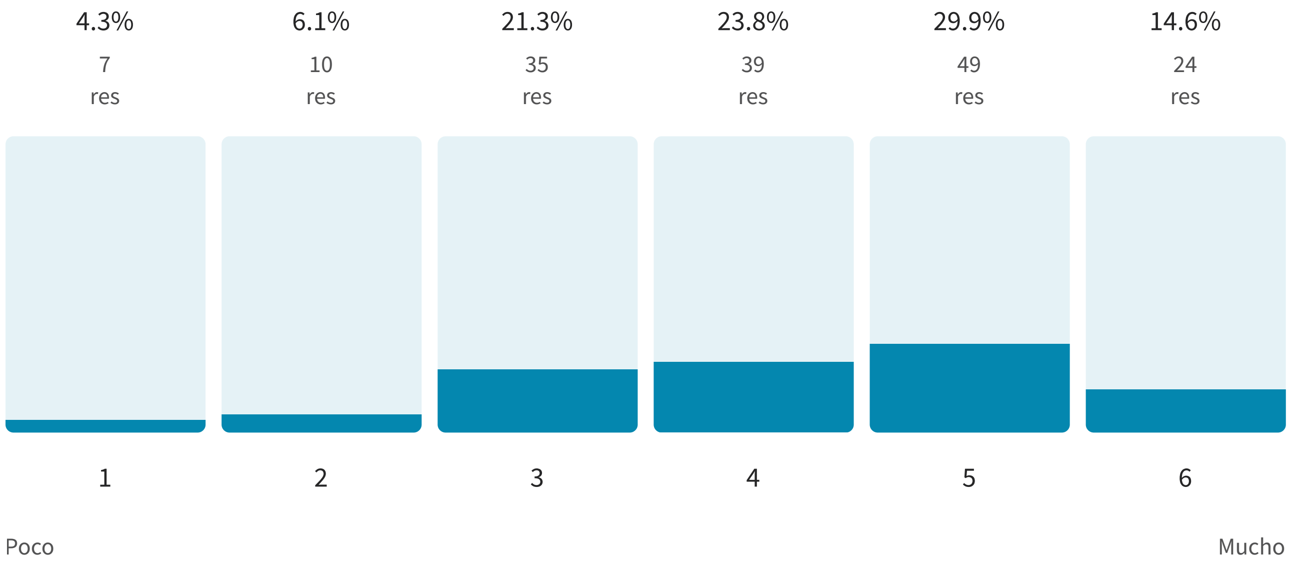

¿En qué medida resuelves las dudas/necesidades de tus pacientes con la consulta no presencial?

164 de 164 personas han respondido

3.3 Puntuación media

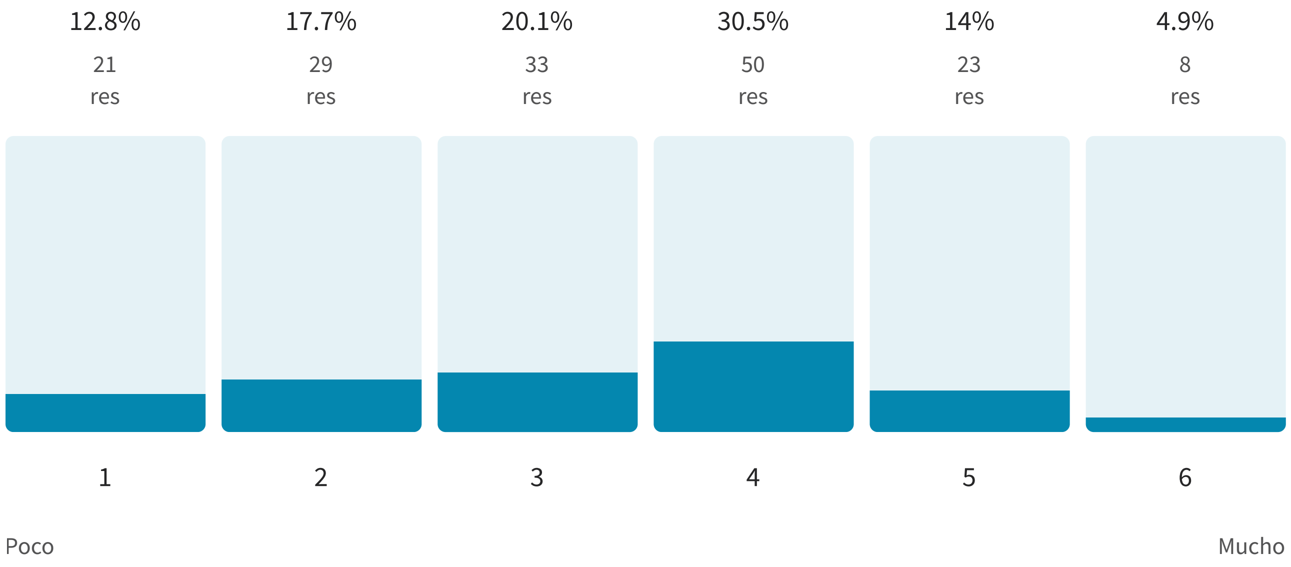

¿Consensúas con tus pacientes objetivos a alcanzar?

164 de 164 personas han respondido

4.8 Puntuación media

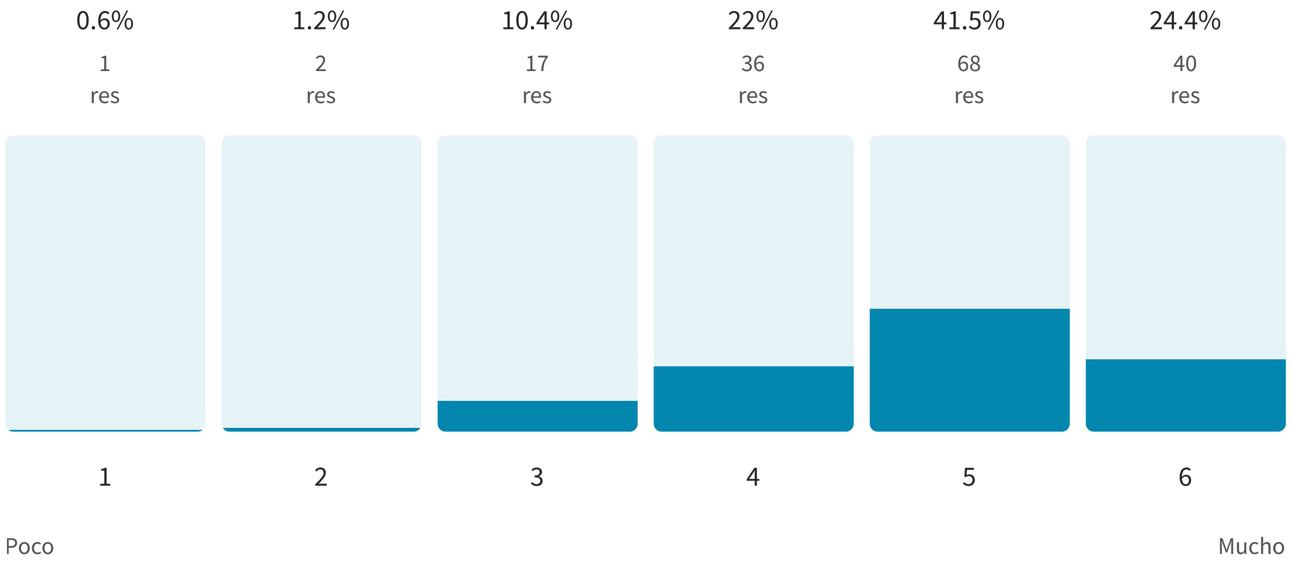

¿En qué medida consideras que la patología afecta a la vida social de su paciente?

164 de 164 personas han respondido

5.5 Puntuación media

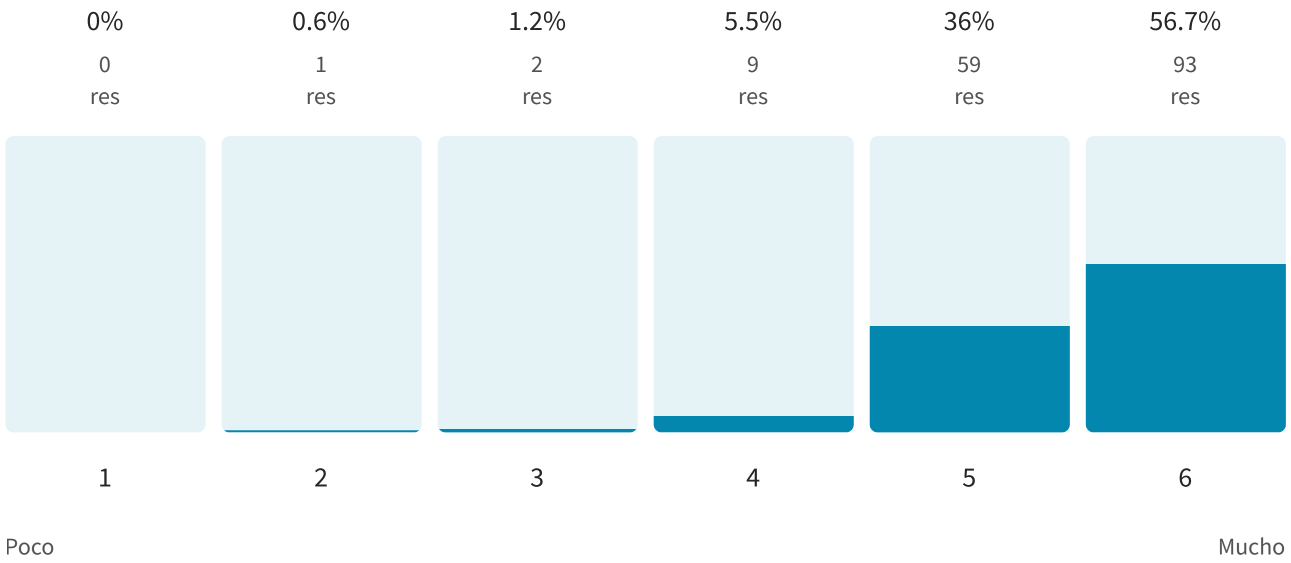

¿En qué medida consideras que su patología afecta a su calidad de vida?

164 de 164 personas han respondido

5.4 Puntuación media

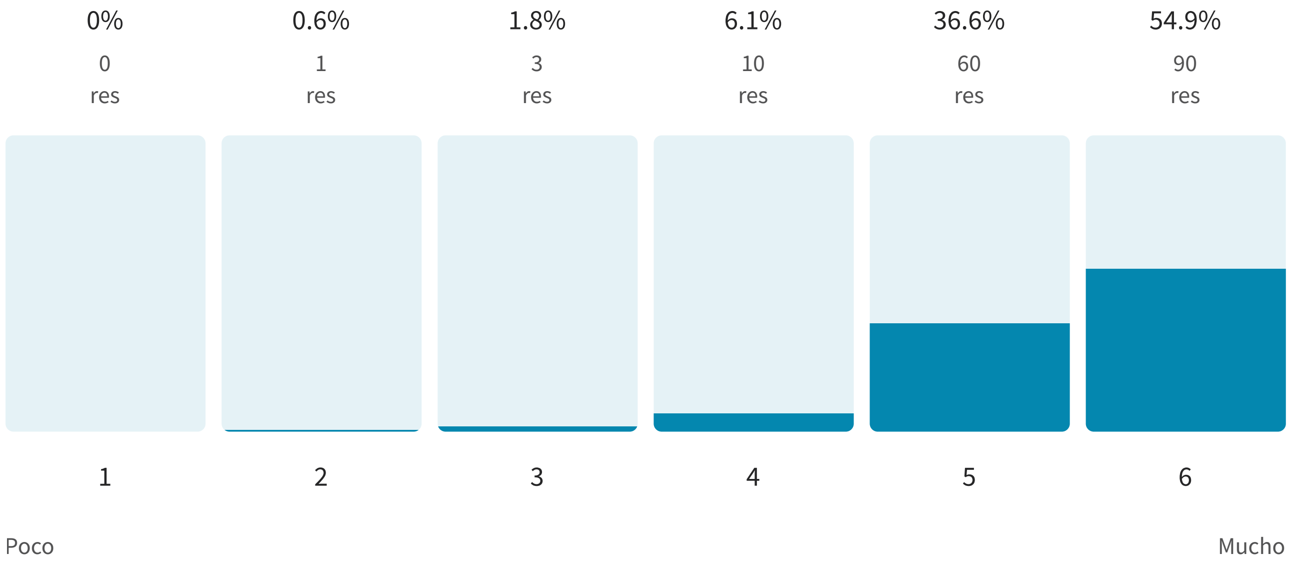

¿En qué medida estás conforme con el tiempo de atención a tus pacientes en consulta de rehabilitación?

164 de 164 personas han respondido

4.5 Puntuación media

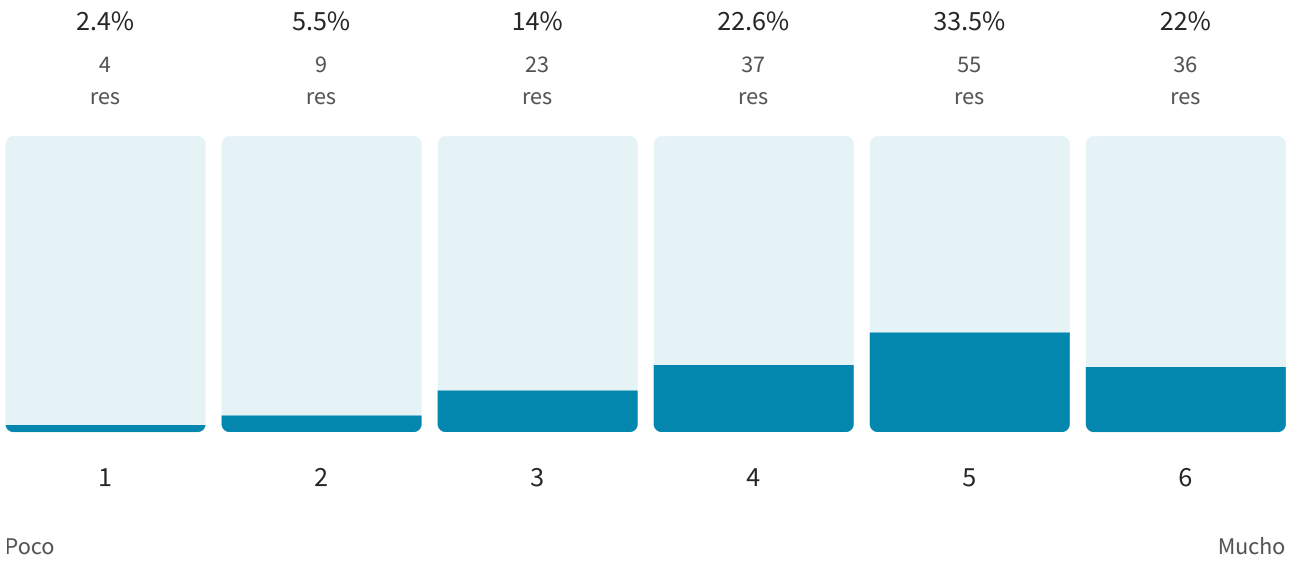

¿En qué medida crees que las revisiones en consulta con el médico rehabilitador ayudan a tus pacientes a continuar realizando los ejercicios en domicilio?

162 de 164 personas han respondido

4.5 Puntuación media

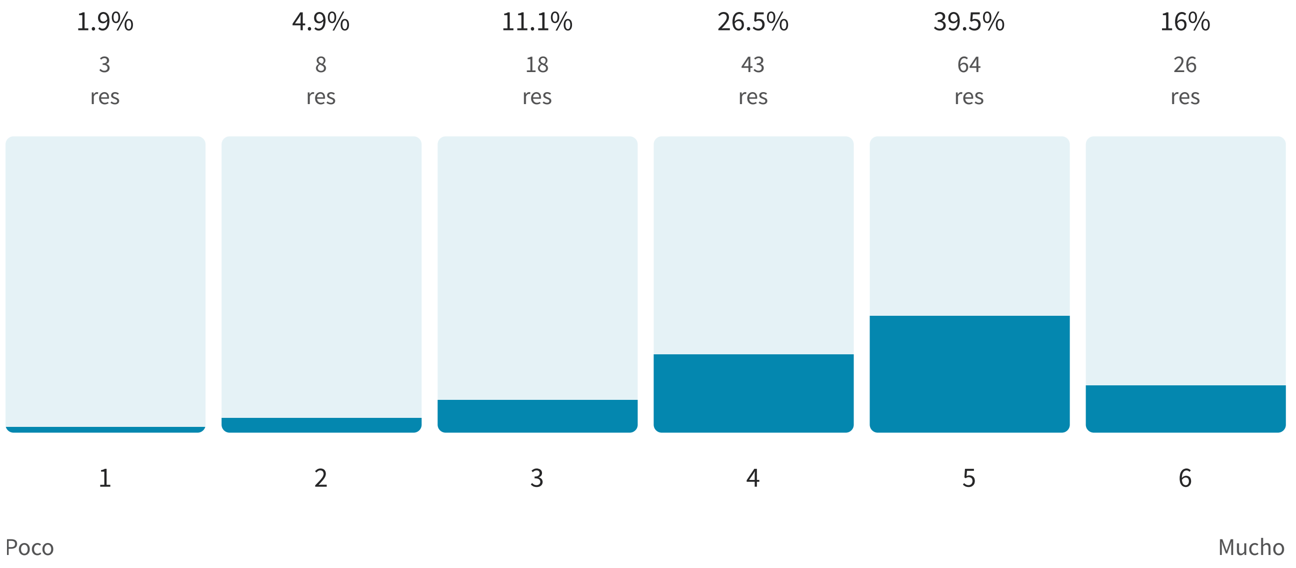

¿En qué medida te gustaría que hubiera algún tipo de aplicación en la que se volcara el entrenamiento domiciliario del paciente y tu equipo le pudiera realizar un seguimiento?

164 de 164 personas han respondido

4.8 Puntuación media

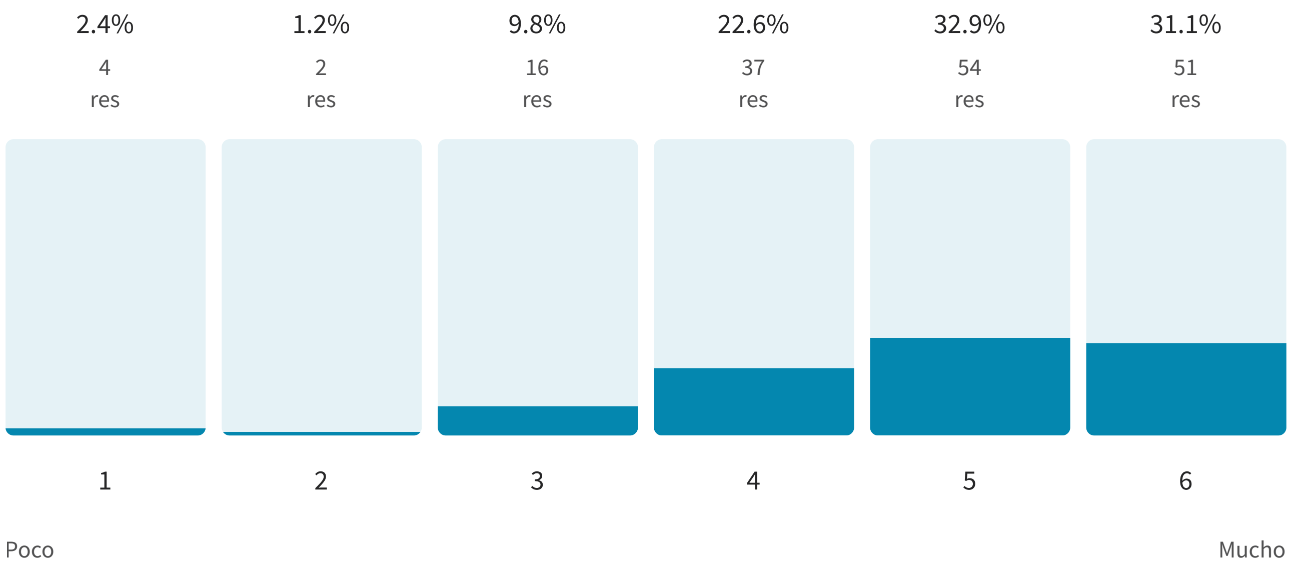

¿Consideras que en tu valoración del paciente con incontinencia tienes en cuenta la limitación social y la repercusión psicológica de esta patología?

164 de 164 personas han respondido

5.1 Puntuación media

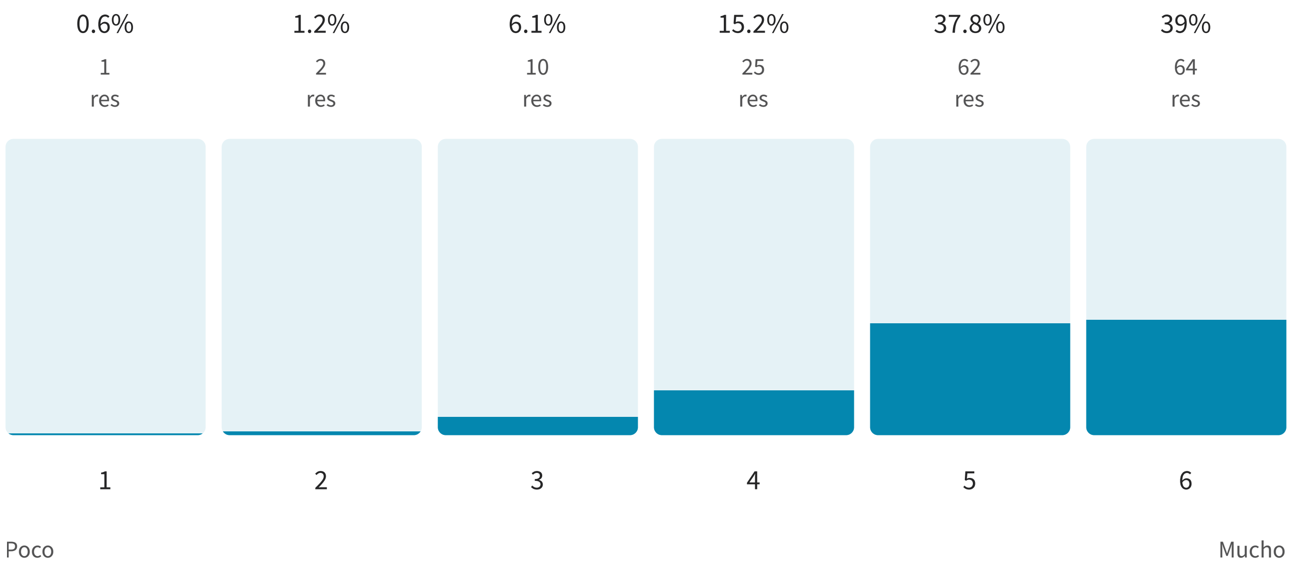

¿En general, en qué grado consideras que tus pacientes pueden necesitar tratamiento de Psicología/Psiquiatría para complementar su tratamiento de la patología de suelo pélvico?

164 de 164 personas han respondido

4.3 Puntuación media

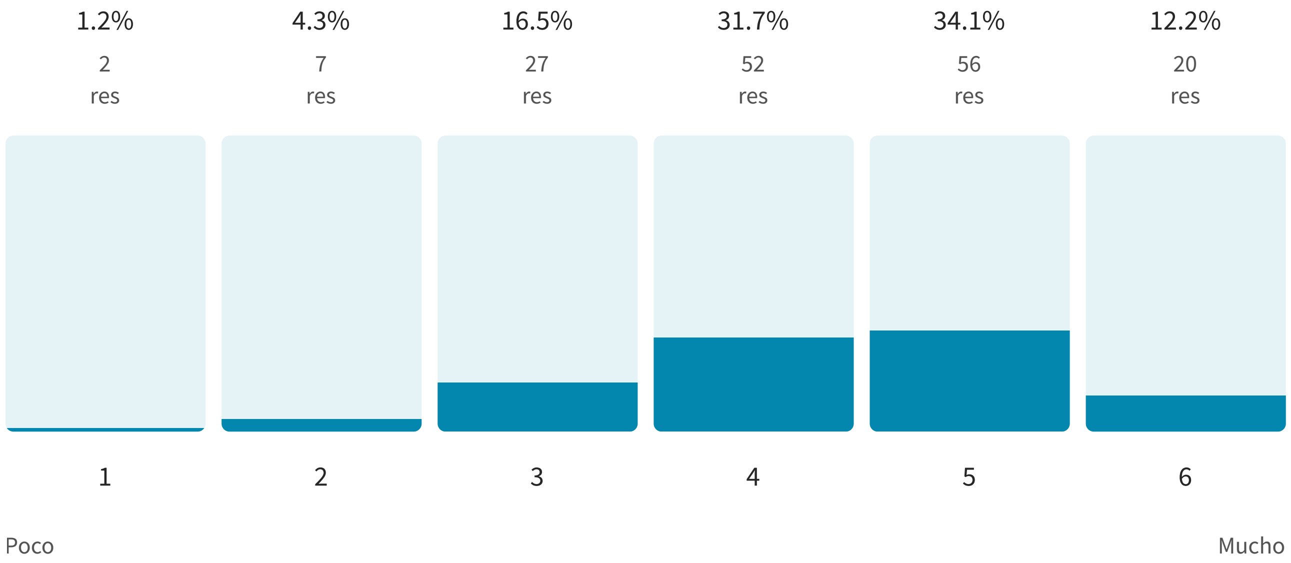

¿Consideras que en tu Área Sanitaria es fácil acceder a una unidad de rehabilitación de suelo pélvico?

164 de 164 personas han respondido

3.6 Puntuación media

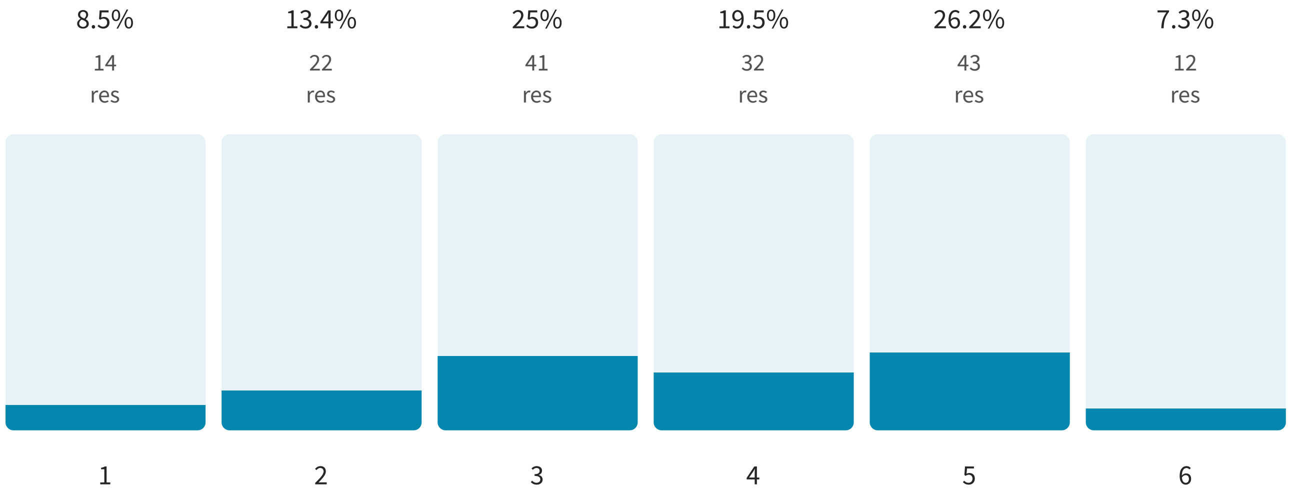

Poco Mucho

¿Consideras que los pacientes deberían de ser derivados antes a la consulta de rehabilitación de Suelo Pélvico?

164 de 164 personas han respondido

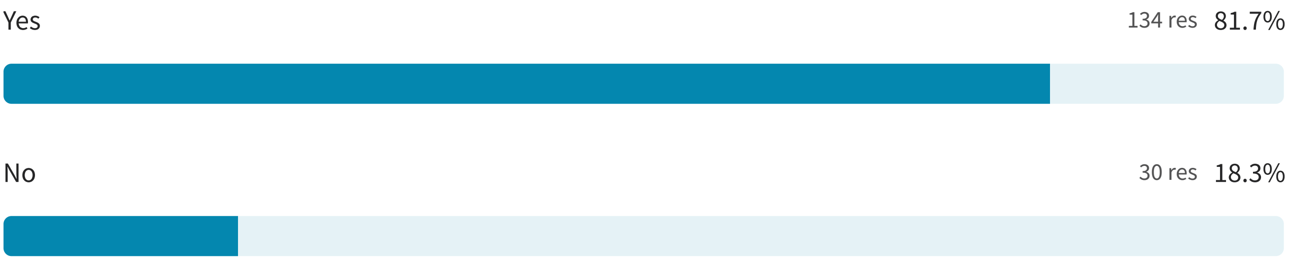

Para aquellos pacientes que deben realizar un diario miccional/defecatorio ¿En general, crees que tus pacientes tienen dificultad para cumplimentarlo?

164 de 164 personas han respondido

3.9 Puntuación media

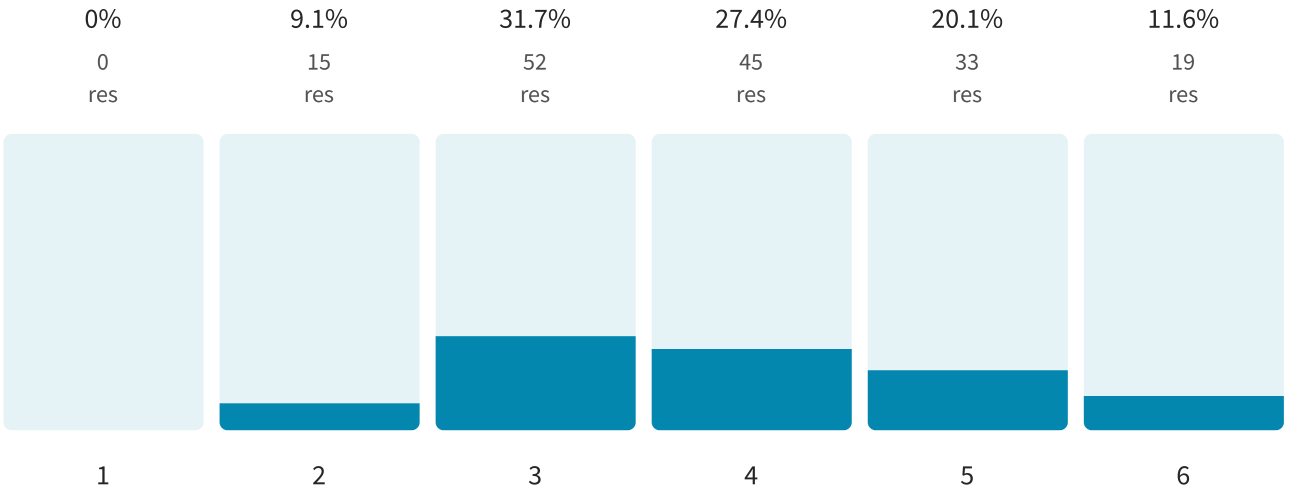

Nunca Ocasionalmen... Siempre

Para aquellos pacientes que deben realizar un diario miccional/defecatorio ¿En general, crees qué les es de utilidad en la reducción/mejoría de la incontinencia?

164 de 164 personas han respondido

4.4 Puntuación media

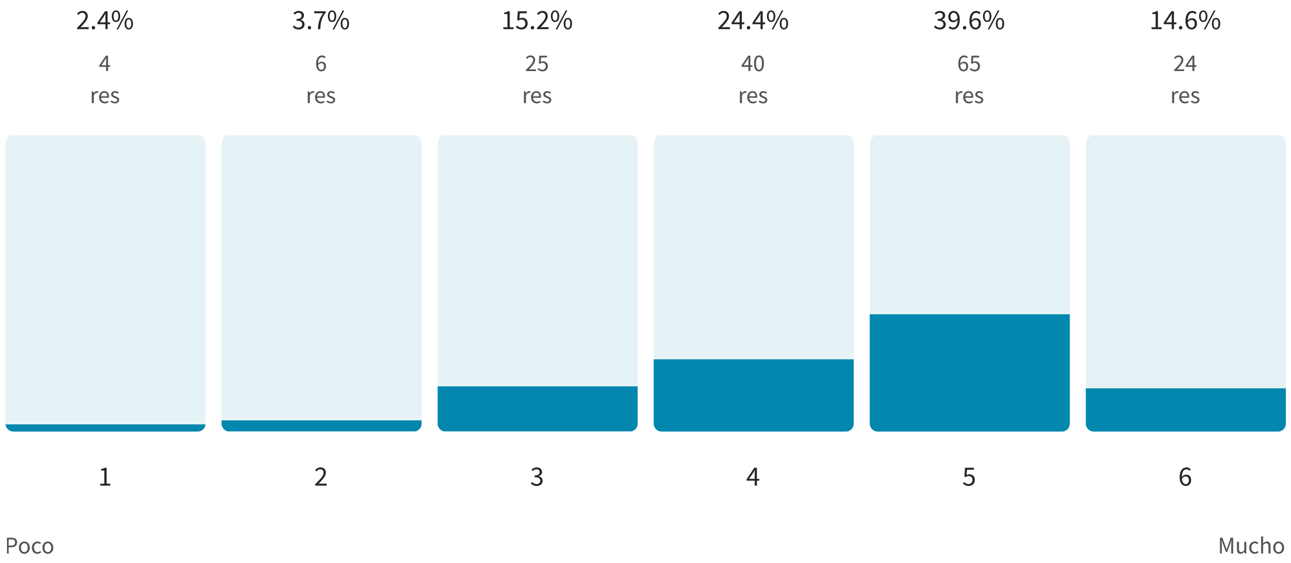

En caso de vejiga hiperactiva/ incontinencia por urgencia/ nocturia o incremento de la frecuencia miccional cuando pautas tratamiento farmacológico ¿En general, en qué grado crees que tus pacientes cumplen con el tratamiento?

164 de 164 personas han respondido

4.0 Puntuación media

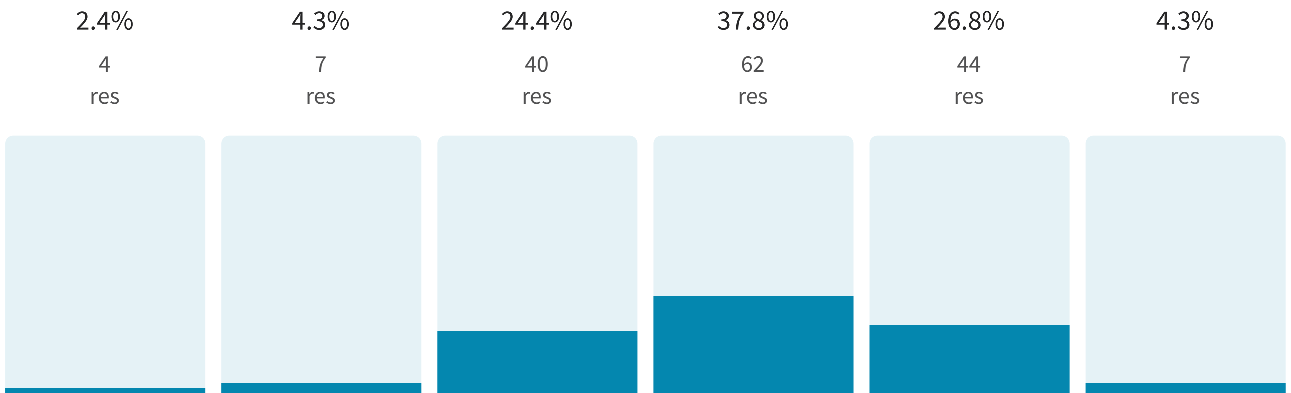

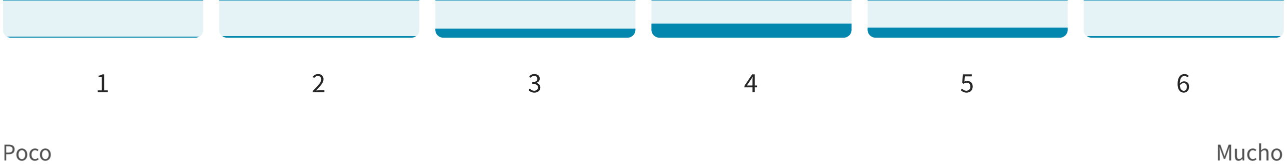

¿En qué medida crees que las instrucciones escritas pueden ayudar a que tus pacientes realicen los ejercicios en su domicilio?

164 de 164 personas han respondido

4.7 Puntuación media

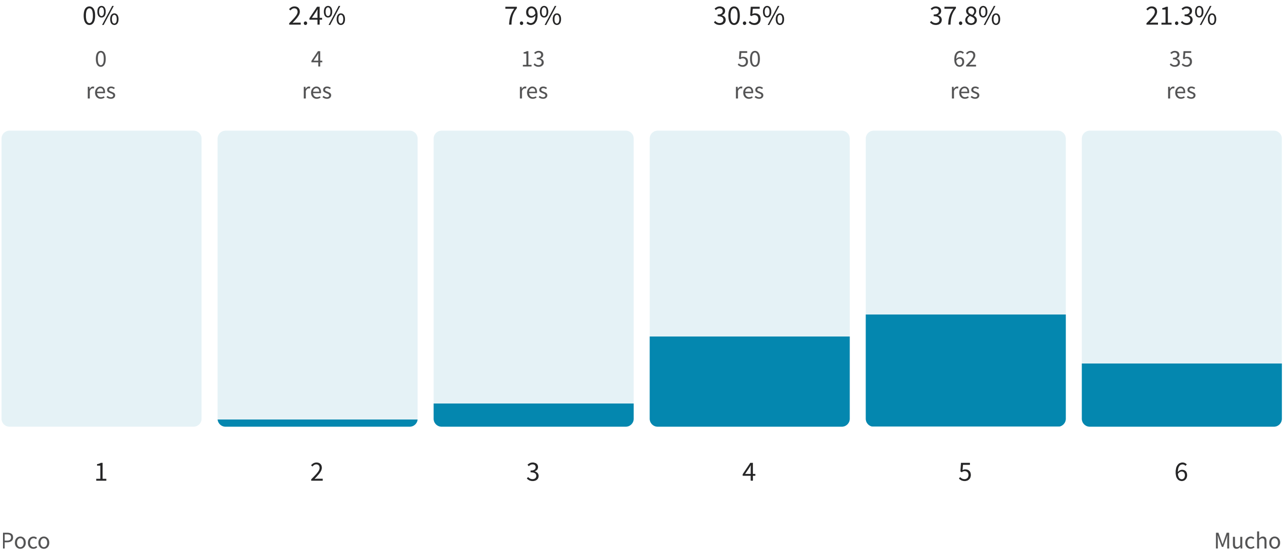

¿En qué medida crees que los ejercicios en video pueden ayudar a que tus pacientes los realicen en su domicilio?

164 de 164 personas han respondido

4.7 Puntuación media

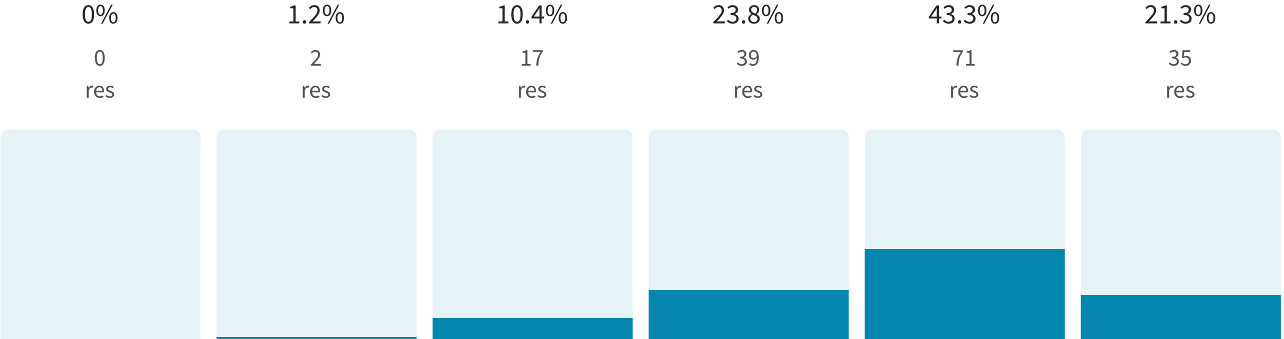

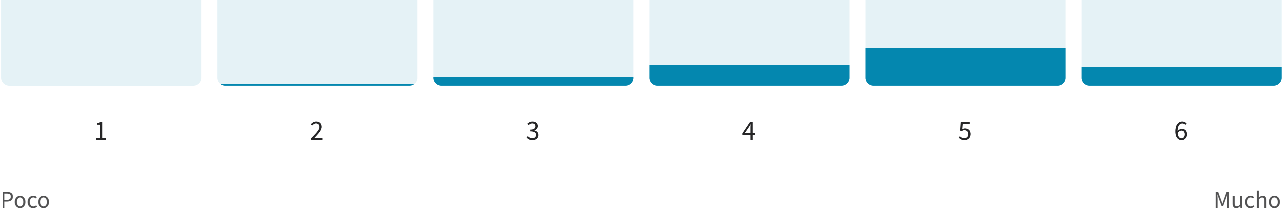

¿En qué medida te parece que a tus pacientes les gustaría asistir a un programa de charlas y tratamiento grupal?

164 de 164 personas han respondido

4.7 Puntuación media

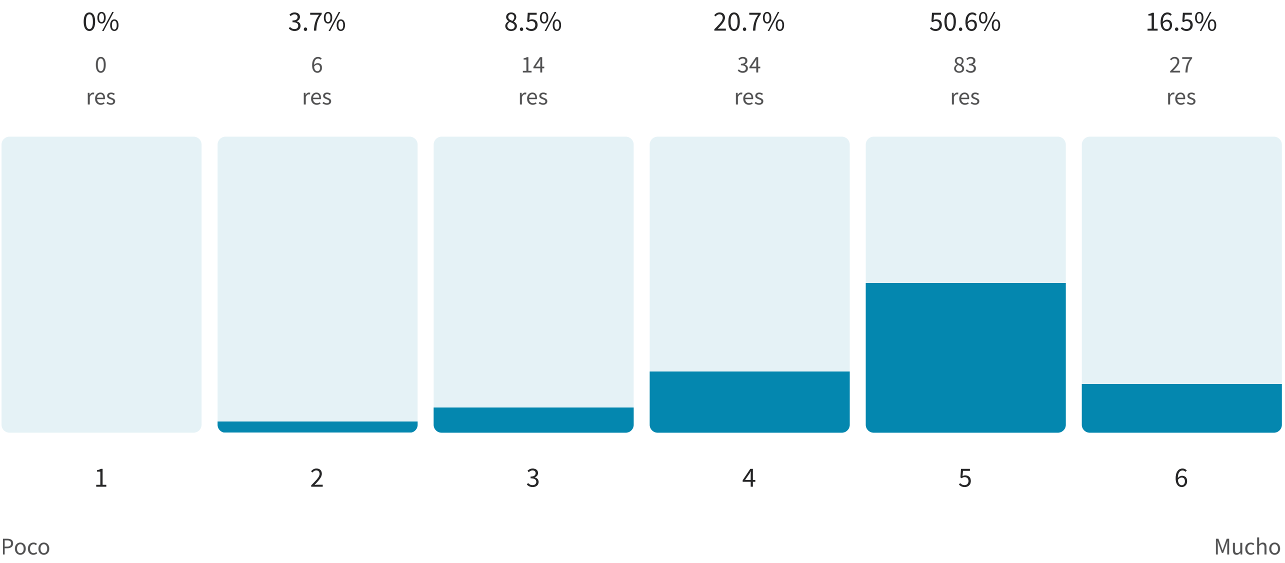

Si tus pacientes ya han realizado tratamiento grupal ¿Cree que les ha sido útil?

103 de 164 personas han respondido

4.6 Puntuación media

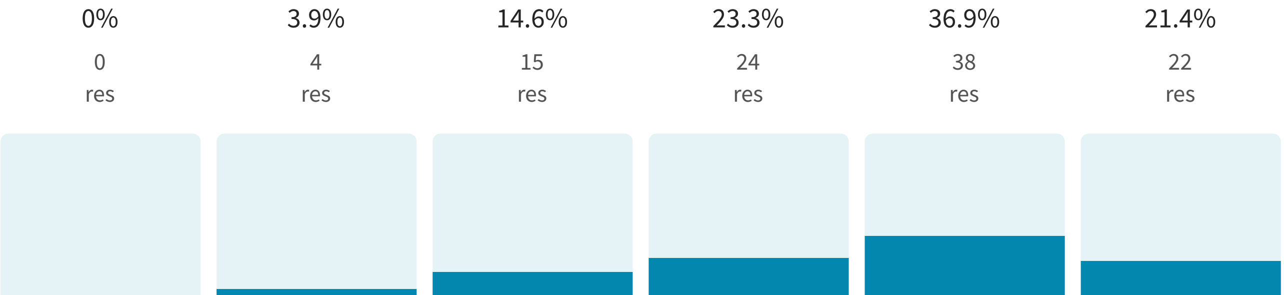

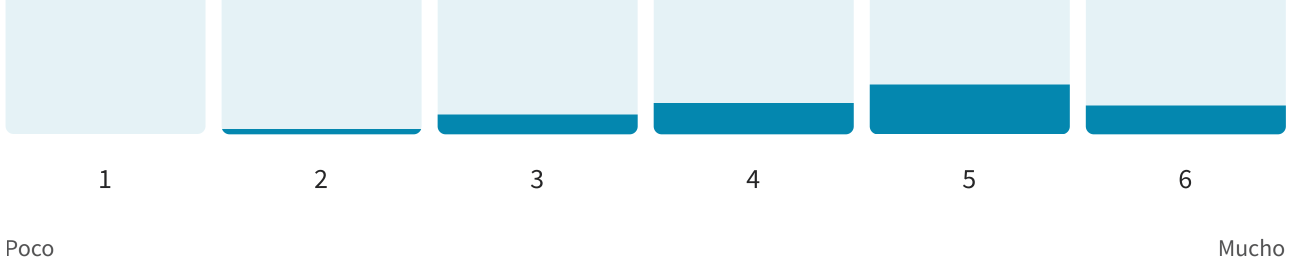

¿Informas a tus pacientes sobre la posibilidad de complementar los ejercicios de Suelo Pélvico en su domicilio con otros dispositivos?

164 de 164 personas han respondido

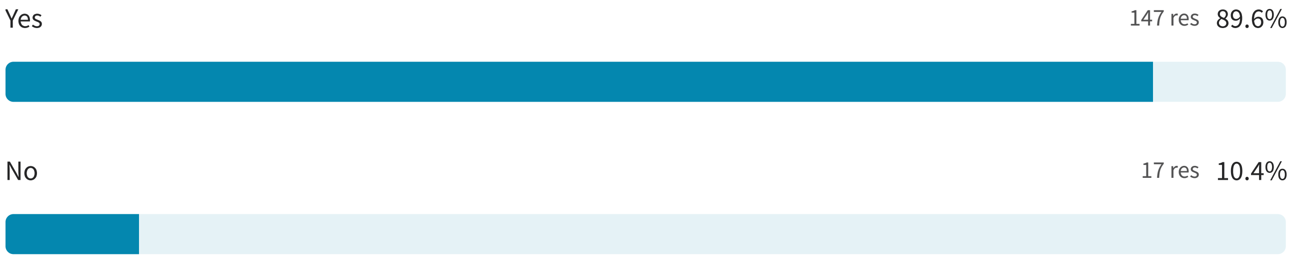

¿Crees que pueden ayudar a que tus pacientes realicen los ejercicios en su domicilio?

147 de 164 personas han respondido

4.8 Puntuación media

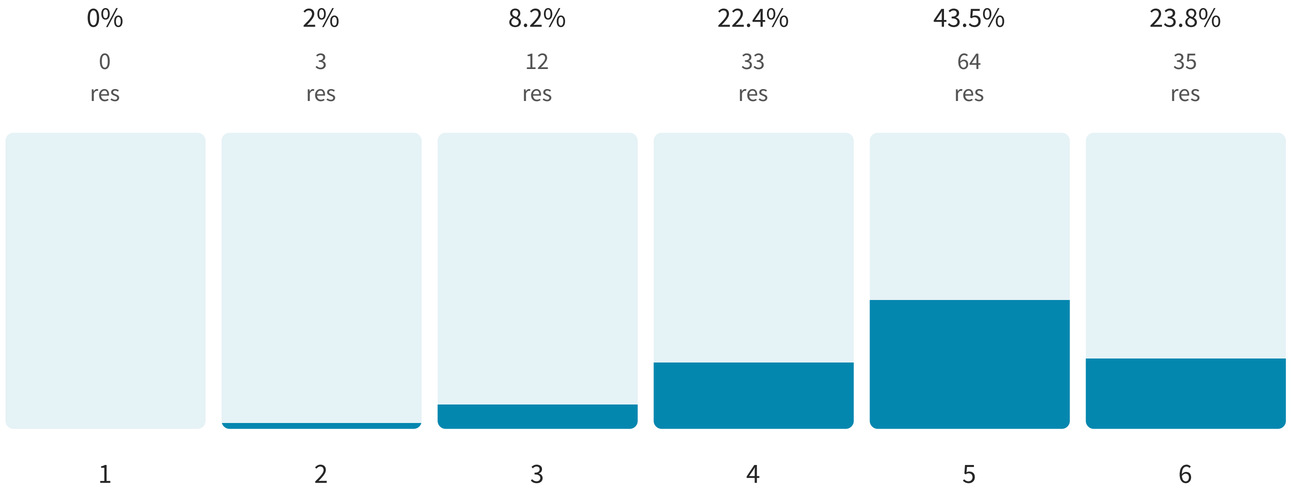

Poco Mucho

¿En qué medida crees que el uso de aplicaciones móviles, recomendadas por un profesional, pueden ayudar que tus pacientes realicen los ejercicios en su domicilio?

164 de 164 personas han respondido

4.5 Puntuación media

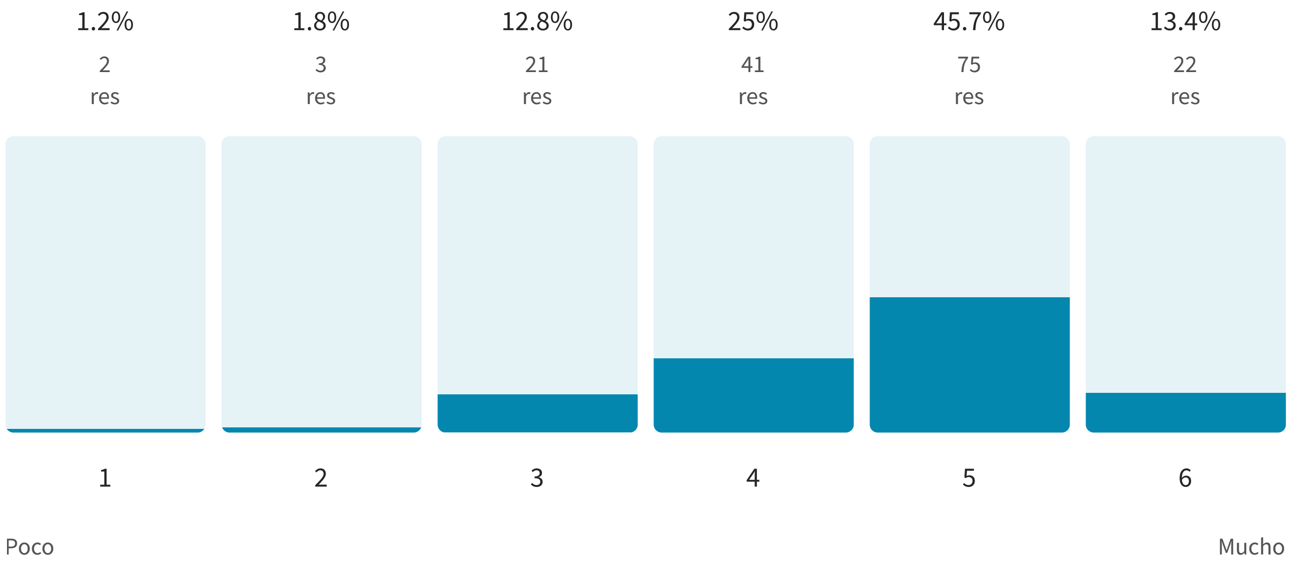

Supplement: Supplementary file 1 [file healthcare-14-02520-s001.zip › Supplementary File S2, Healthcare professional questionnaire.pdf]
