# Supplementary material for: Translating Patient–Professional Divergence into Care-Improvement Priorities in Pelvic Floor Rehabilitation: A Delphi-Dialogue Study
Source: Healthcare (Basel). 2026 Aug 12;14(16):2520. doi: 10.3390/healthcare14162520 (PMC13512020; doi:10.3390/healthcare14162520)
Supplement: Supplementary file 1 [file healthcare-14-02520-s001.zip › Supplementary File S1, Patient questionnaire.pdf]

DDPP - Suelo Pelvico - Pacientes

226 respuestas

¿Con qué género se identifica?

226 de 226 personas han respondido

Mujer	199 res	88.1%
Hombre	27 res	11.9%
No Binario	0 res	0%

¿Cuál es su nivel educativo?

226 de 226 personas han respondido

Estudios universitarios	113 res	50%
Estudios secundarios/ formación profesional	79 res	35%
Estudios primarios	31 res	13.7%

Sin educación formal 3 res 1.3%

¿Dónde vive usted?

226 de 226 personas han respondido

Domicilio particular de forma autónoma. 212 res 93.8%

Domicilio particular con cuidador familiar. 11 res 4.9%

Residencia. 3 res 1.3%

Domicilio particular con cuidador profesional no familiar. 0 res 0%

Piso tutelado. 0 res 0%

¿En qué Comunidad Autónoma reside?

226 de 226 personas han respondido

Comunidad de Madrid 50 res 22.1%

Castilla-La Mancha 34 res 15%

Canarias 28 res 12.4%

Principado de Asturias	16 res	7.1%
Galicia	10 res	4.4%
Illes Balears	9 res	4%
Comunitat Valenciana	2 res	0.9%
Cantabria	1 res	0.4%
Comunidad Foral de Navarra	1 res	0.4%
Andalucía	0 res	0%
Aragón	0 res	0%
Castilla y León	0 res	0%
Cataluña	0 res	0%
Ciudad Autónoma de Ceuta	0 res	0%
Ciudad Autónoma de Melilla	0 res	0%
Extremadura	0 res	0%
La Rioja	0 res	0%
País Vasco	0 res	0%

Región de Murcia 0 res 0%

¿Cuál de las siguientes patologías de suelo pélvico padece?

226 de 226 personas han respondido

Incontinencia Urinaria 146 res 64.6%

Incontinencia Fecal 74 res 32.7%

Incontinencia Gases 65 res 28.8%

Prolapso 41 res 18.1%

Dolor pélvico 39 res 17.3%

¿Cuál es su sintoma principal?

226 de 226 personas han respondido

Incontinencia Urinaria 114 res 50.4%

Incontinencia Fecal 52 res 23%

Dolor pélvico 22 res 9.7%

Prolapso 22 res 9.7%

Incontinencia Gases 16 res 7.1%

¿Cuánto tiempo ha transcurrido desde el inicio del síntoma principal?

226 de 226 personas han respondido

Entre 1 y 5 años 102 res 45.1%

Menos de 1 año 43 res 19%

Entre 6 y 10 años 35 res 15.5%

Entre 11 y 15 años 19 res 8.4%

Mas de 20 años 19 res 8.4%

Entre15 y 20 años 8 res 3.5%

¿Quién le diagnosticó su patología de suelo pélvico?

226 de 226 personas han respondido

Ginecología 67 res 29.6%

Urología 41 res 18.1%

Coloproctología 29 res 12.8%

Digestivo 25 res 11.1%

Medicina Física y Rehabilitación 18 res 8%

Atención Primaria 14 res 6.2%

Fisioterapia 9 res 4%

Matrona 8 res 3.5%

Other 15 res 6.6%

¿Ha sido valorado usted en un servicio de Medicina Física y Rehabilitación por su patología de suelo pélvico?

226 de 226 personas han respondido

Yes 182 res 80.5%

No	44 res	19.5%

¿Quién le lo derivó al servicio de Medicina Física y Rehabilitación?

182 de 226 personas han respondido

Ginecología	59 res	32.4%
Urología	32 res	17.6%
Digestivo	27 res	14.8%
Atención Primaria	19 res	10.4%
Coloproctología	18 res	9.9%
Medicina Física y Rehabilitación	11 res	6%
Fisioterapia	4 res	2.2%
Matrona	1 res	0.5%

Other 11 res 6%

¿Cuánto tiempo pasó desde su diagnóstico hasta que le valoró un médico rehabilitador?

182 de 226 personas han respondido

Menos de 6 meses 84 res 46.2%

Entre 6 meses y 1 año 62 res 34.1%

Entre 1 y 3 años 27 res 14.8%

Más de 6 años 5 res 2.7%

Entre 4 y 6 años 4 res 2.2%

¿Cuánto tiempo lleva o estuvo en seguimiento en un servicio de Medicina Física y Rehabilitación?

182 de 226 personas han respondido

Menos de 1 año 119 res 65.4%

Entre 1 y 5 años 45 res 24.7%

No me siguieron en un Servicio de Medicina Física y Rehabilitación 11 res 6%

Entre 5 y 10 años 4 res 2.2%

Más de 10 años 3 res 1.6%

¿Está satisfecho con la atención que recibe o ha recibido por parte de su médico rehabilitador para tratar su síntoma principal?

182 de 226 personas han respondido

5.2 Puntuación media

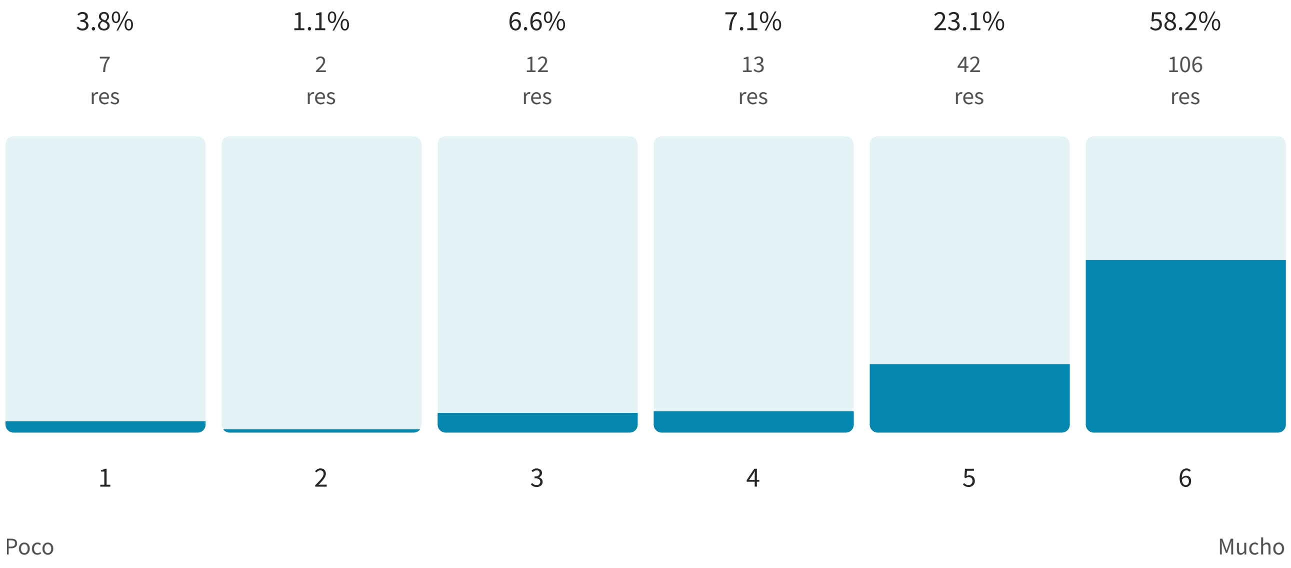

¿Si no le han derivado a un Servicio de Medicina Física y Rehabilitación, a qué cree que se ha debido?

42 de 226 personas han respondido

Desconozco si hay unidades de Rehabilitación de Suelo Pélvico en mi Área Sanitaria 20 res 47.6%

Hay, pero no me han derivado 12 res 28.6%

No hay Unidades de Rehabilitación de Suelo Pélvico en mi Área Sanitaria 2 res 4.8%

Other 8 res 19%

¿Ha recibido suficiente información sobre su patología de suelo pélvico?

226 de 226 personas han respondido

4.8 Puntuación media

1	2	3	4	5	6
7.1%	4.9%	6.2%	12.8%	25.7%	43.4%
16 res	11 res	14 res	29 res	58 res	98 res

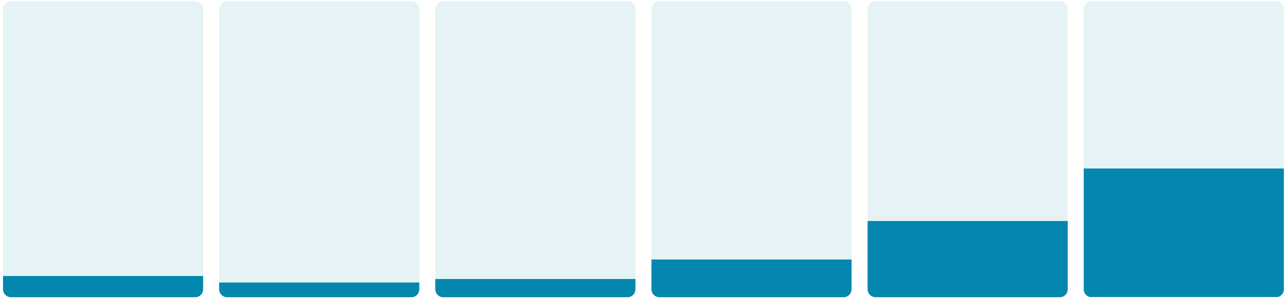

1 2 3 4 5 6

Poco Mucho

¿Ha recibido dicha información en el momento adecuado para usted?

226 de 226 personas han respondido

4.2 Puntuación media

13.3%	6.2%	14.6%	14.2%	17.7%	34.1%
30 res	14 res	33 res	32 res	40 res	77 res

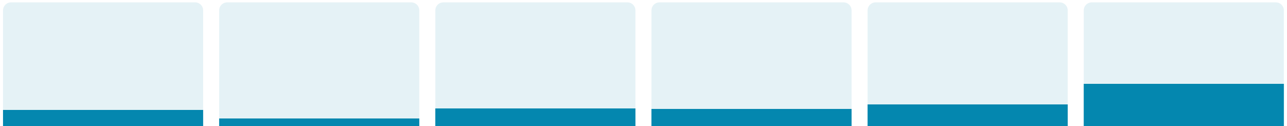

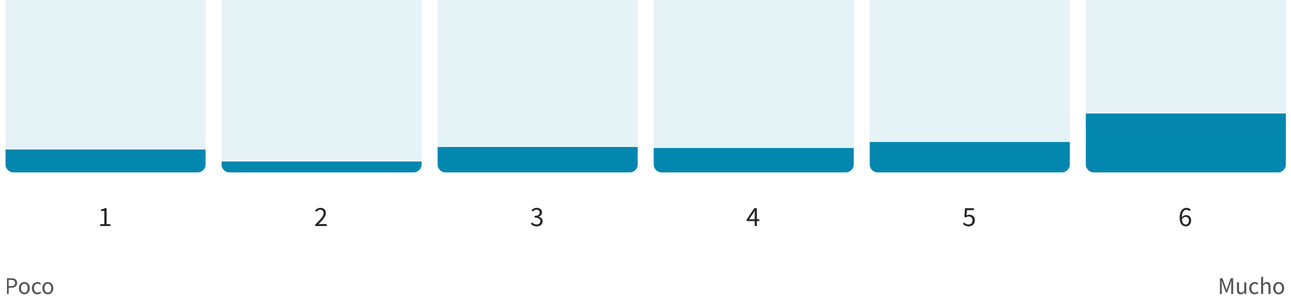

¿Quién le ha informado sobre su patología de suelo pélvico?

226 de 226 personas han respondido

Medicina Física y Rehabilitación	67 res	29.6%
Ginecología	35 res	15.5%
Fisioterapia	29 res	12.8%
Urología	26 res	11.5%
Coloproctología	25 res	11.1%
Digestivo	19 res	8.4%
Atención Primaria	7 res	3.1%
Matrona	5 res	2.2%
Asociación de Pacientes	3 res	1.3%
Enfermería	1 res	0.4%
Other	9 res	4%

¿Ha seguido o está siguiendo usted tratamiento de rehabilitación/ fisioterapia por su patología de suelo pélvico?

226 de 226 personas han respondido

Yes	162 res	71.7%

No 64 res 28.3%

¿Está satisfecho con la atención que recibe o ha recibido por parte de su fisioterapeuta para tratar su síntoma principal?

162 de 226 personas han respondido

5.4 Puntuación media

1	2	3	4	5	6
3.1%	1.2%	3.7%	4.9%	17.3%	69.8%
5 res	2 res	6 res	8 res	28 res	113 res

Poco — Mucho

¿Cuánto hace que terminó su programa presencial?

162 de 226 personas han respondido

Estoy haciéndolo 97 res 59.9%

Más de 1 año 24 res 14.8%

Entre 3 y 6 meses 12 res 7.4%

Menos de 1 mes 10 res 6.2%

Entre 1 y 3 meses 7 res 4.3%

Entre 6 meses y 1 año 6 res 3.7%

No he realizado programa de terapia presencial 6 res 3.7%

¿Cuántas sesiones de fisioterapia ha necesitado para aprender los ejercicios?

162 de 226 personas han respondido

Entre 5 y 10 57 res 35.2%

Menos de 5 44 res 27.2%

Entre 11 y 15 26 res 16%

Mas de 15 22 res 13.6%

No he realizado programa de terapia presencial 13 res 8%

¿Qué tratamiento ha recibido o recibe para su patología de suelo pélvico?

226 de 226 personas han respondido

Ejercicios	158 res	69.9%
Electroestimulación	89 res	39.4%
Biofeedback	55 res	24.3%
Fármacos	40 res	17.7%
Neuromodulación Tibial posterior	37 res	16.4%
Cirugía	22 res	9.7%
Pesarios	8 res	3.5%
Infiltraciones con corticoides y/o anestésicos	7 res	3.1%
Toxina	7 res	3.1%
Other	23 res	10.2%

¿Dónde ha realizado el tratamiento?

226 de 226 personas han respondido

Hospital	151 res	66.8%
Centro privado	31 res	13.7%

Domicilio (Autotratamiento) 31 res 13.7%

Centro de salud 27 res 11.9%

No he realizado tratamiento 18 res 8%

Other 11 res 4.9%

¿Ha tenido usted que realizar un diario miccional?

226 de 226 personas han respondido

Yes 97 res 42.9%

No 129 res 57.1%

¿Qué es lo que cree que puede dificultar en mayor medida realizar el diario?

97 de 226 personas han respondido

No es complatible con mi vida diaria 31 res 32%

No lo entiendo 13 res 13.4%

Es muy complejo 12 res 12.4%

No me dieron suficiente explicación 9 res 9.3%

Other 32 res 33%

¿Ha recibido tratamiento farmacológico para su incontinencia urinaria?

226 de 226 personas han respondido

Yes 39 res 17.3%

No 187 res 82.7%

Si lo ha dejado de tomar ¿cuál ha sido el motivo?

39 de 226 personas han respondido

Continuo tomándolo 15 res 38.5%

Efectos secundarios 9 res 23.1%

Me lo ha retirado un especialista 7 res 17.9%

No mejoraba 6 res 15.4%

Me lo ha retirado mi medico de atención primaria 4 res 10.3%

Me he cansado de tomarlo 2 res 5.1%

Es caro 1 res 2.6%

Me lo han cambiado por otro 1 res 2.6%

Olvido 1 res 2.6%

¿Durante cuánto tiempo lo tomó?

39 de 226 personas han respondido

Continuo tomándolo 12 res 30.8%

Entre 3 y 6 meses 11 res 28.2%

Entre 6 meses y 1 año 7 res 17.9%

Menos de 1 mes 5 res 12.8%

Mas de 1 año 4 res 10.3%

¿Aplica en su vida diaria las recomendaciones dadas por los profesionales para su problema de suelo pélvico?

224 de 226 personas han respondido

4.5 Puntuación media

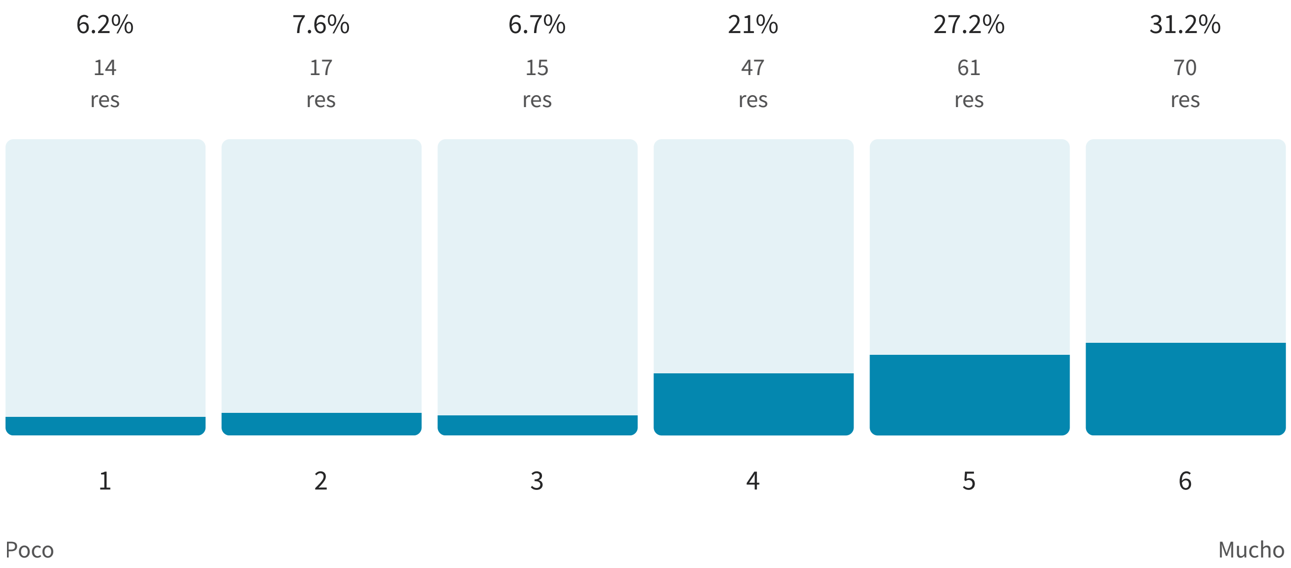

¿Su urólogo o ginecólogo le entregó una hoja de ejercicios de suelo pélvico para realizar en su domicilio?

226 de 226 personas han respondido

No 112 res 49.6%

Si y me enseñó a realizarlos 81 res 35.8%

Si y no me enseñó a realizarlos 33 res 14.6%

¿Mejoró su incontinencia con esos ejercicios entregados en papel?

114 de 226 personas han respondido

Yes 61 res 53.5%

No 53 res 46.5%

¿Tiene alguna enfermedad crónica además de su patología de suelo pélvico?

226 de 226 personas han respondido

Yes 92 res 40.7%

No 134 res 59.3%

¿Tiene dificultades de movilidad?

226 de 226 personas han respondido

Yes 31 res 13.7%

No 195 res 86.3%

¿Trabajaba usted antes de comenzar con su problema de suelo pélvico?

226 de 226 personas han respondido

Yes 172 res 76.1%

No 54 res 23.9%

¿Ha perdido su trabajo debido a su problema de suelo pélvico?

172 de 226 personas han respondido

Yes 17 res 9.9%

No 155 res 90.1%

¿Ha cambiado de trabajo como consecuencia de su problema de suelo pélvico?

17 de 226 personas han respondido

Yes 10 res 58.8%

No 7 res 41.2%

¿Tiene usted reconocida una incapacidad laboral como consecuencia de su patología de suelo pélvico?

226 de 226 personas han respondido

Yes 25 res 11.1%

No 201 res 88.9%

Indique las situaciones en las que la patología de suelo pélvico le provoca dificultades

226 de 226 personas han respondido

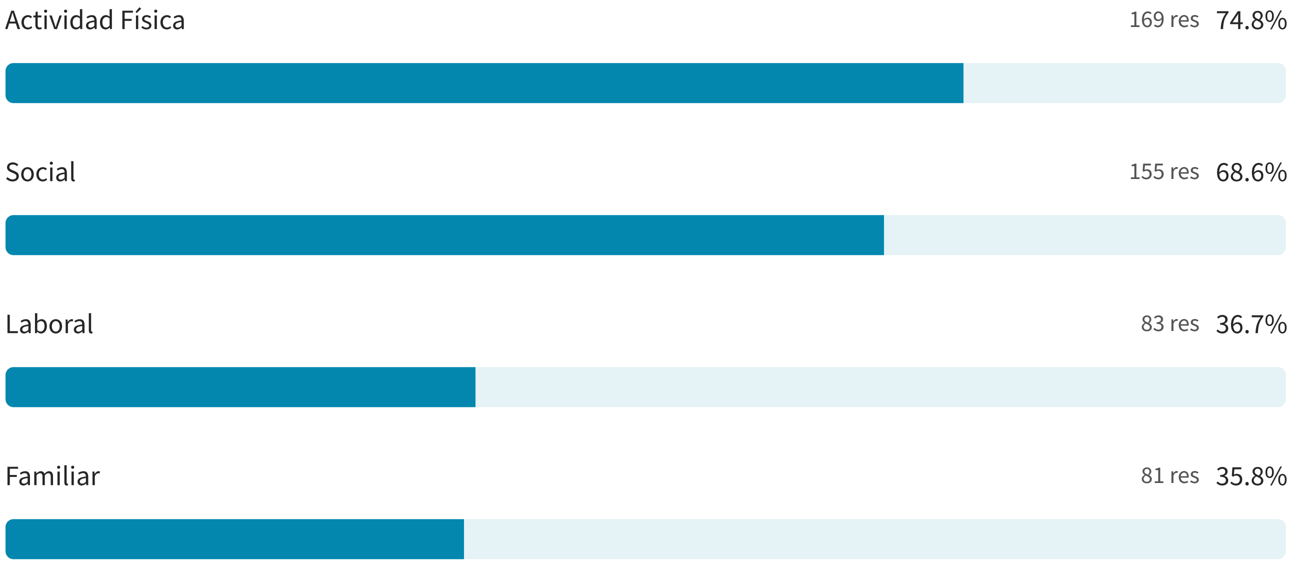

¿En qué medida el equipo de Medicina Física y Rehabilitación se implica en su atención?

180 de 226 personas han respondido

4.8 Puntuación media

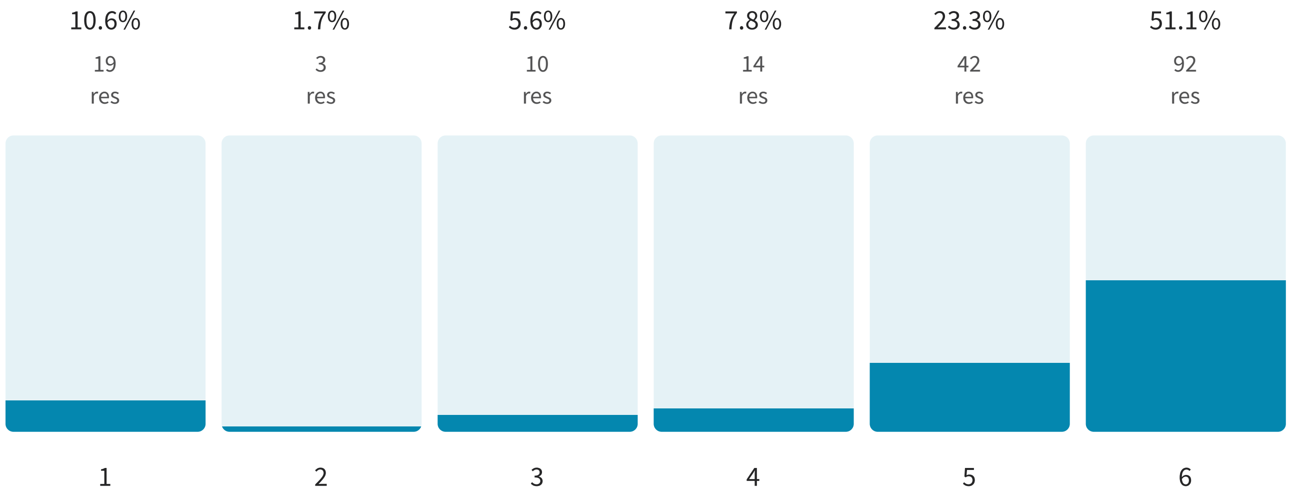

Poco Mucho

¿En qué medida las revisiones presenciales en consulta de rehabilitación cumplen sus expectativas?

180 de 226 personas han respondido

4.6 Puntuación media

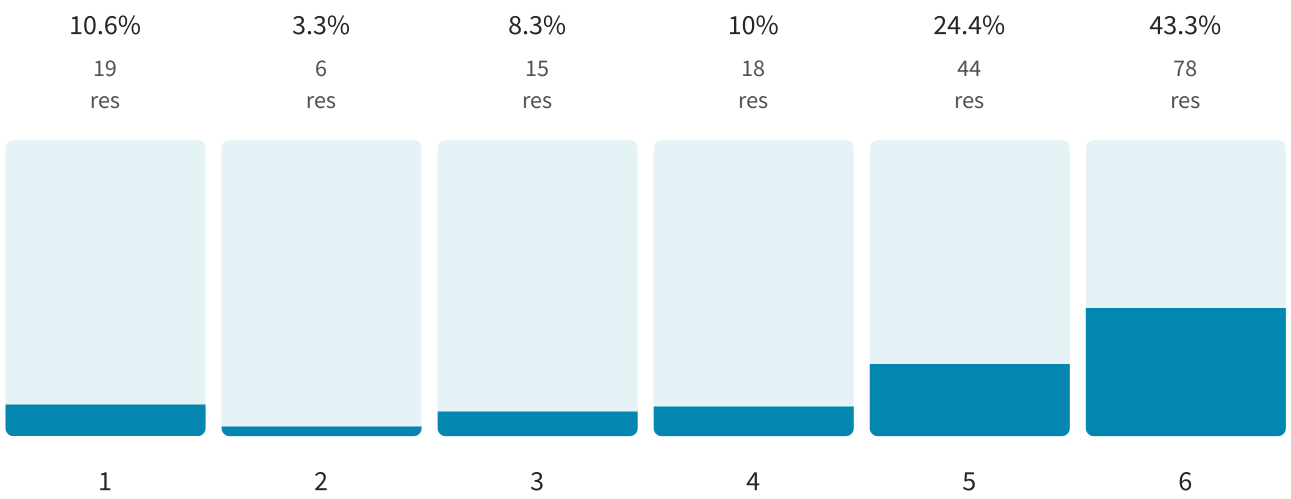

Poco Mucho

¿En qué medida se considera usted capacitado para usar Internet en relación a su patología?

226 de 226 personas han respondido

4.7 Puntuación media

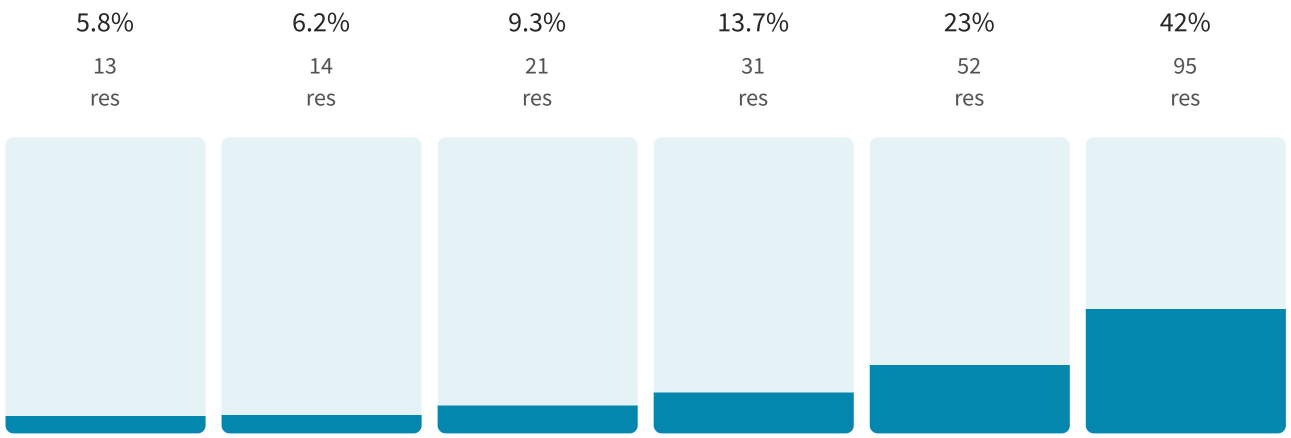

1 2 3 4 5 6

Poco Mucho

¿En qué medida considera útil la prescripción de programas de tratamiento para hacer en su casa?

226 de 226 personas han respondido

4.6 Puntuación media

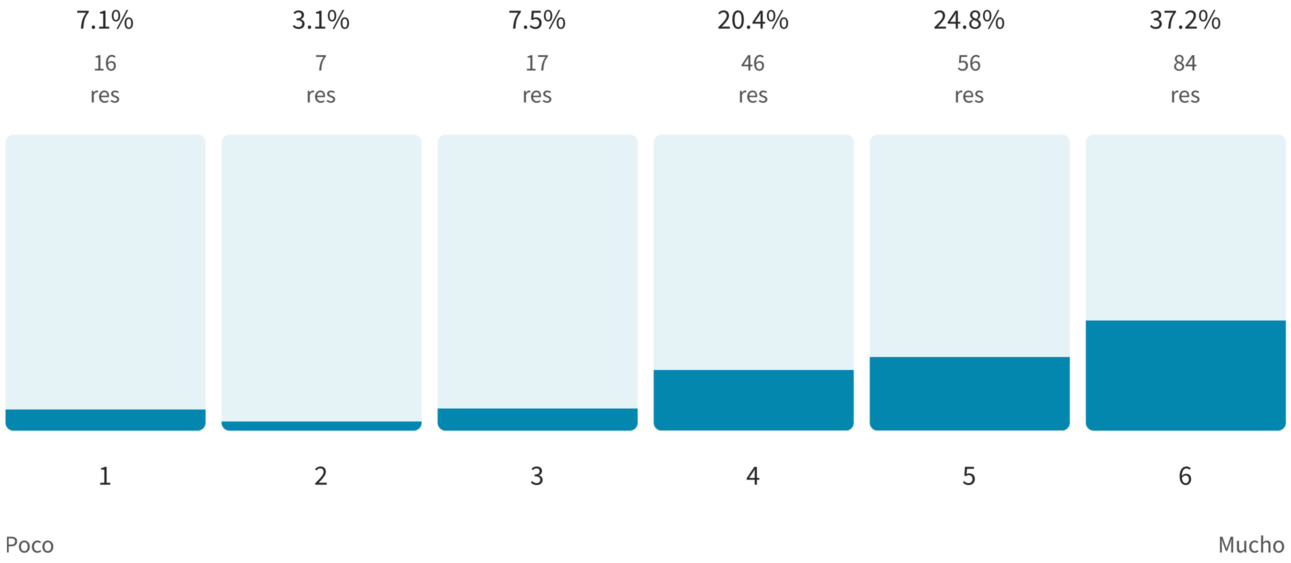

¿En qué grado cumple los programas de tratamiento que le han pautado?

226 de 226 personas han respondido

4.4 Puntuación media

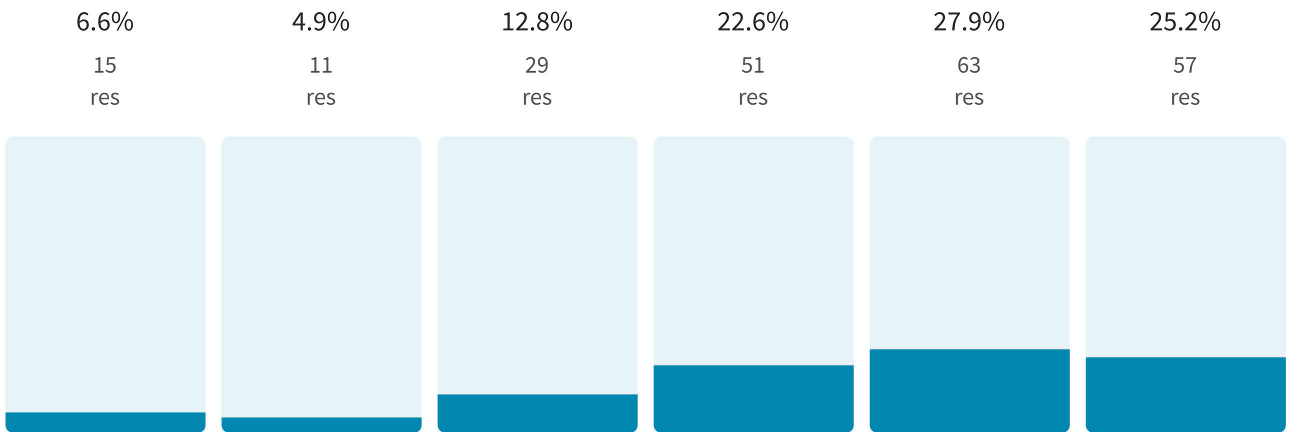

1	2	3	4	5	6

Poco — Mucho

¿Cuál es la principal dificultad que encuentra para cumplirlos?

226 de 226 personas han respondido

Respuesta	Respuestas	Porcentaje
No tengo dificultad	92 res	40.7%
No tengo tiempo	57 res	25.2%
No me acuerdo	40 res	17.7%
Son difíciles	21 res	9.3%
No los he aprendido	16 res	7.1%

¿En qué medida considera útil comunicarse a distancia con el equipo de Medicina Física y Rehabilitación para realizar los ejercicios pautados?

226 de 226 personas han respondido

4.3 Puntuación media

10.2%	5.8%	9.7%	17.7%	27.4%	29.2%
23 res	13 res	22 res	40 res	62 res	66 res

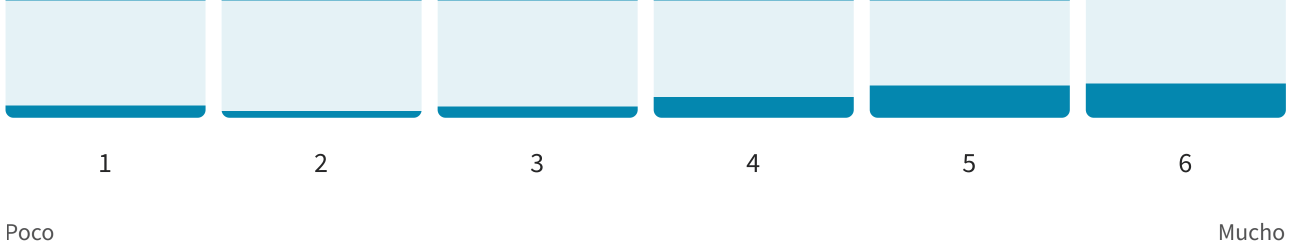

¿Cuál de los siguientes formatos prefiere para realizar la terapia desde su casa?

226 de 226 personas han respondido

Video modelo 116 res 51.3%

Instrucciones escritas 50 res 22.1%

App 48 res 21.2%

Other 12 res 5.3%

¿Cuál de los siguientes canales prefiere para comunicarse con el equipo de Medicina Física y Rehabilitación?

226 de 226 personas han respondido

Whatsapp 84 res 37.2%

Teléfono 42 res 18.6%

Videollamada 31 res 13.7%

Correo electrónico 26 res 11.5%

Plataforma propia 18 res 8%

App 11 res 4.9%

Facebook / Redes Sociales 0 res 0%

Other 14 res 6.2%

¿De cuáles de las siguientes opciones dispone para comunicación con su médico?

226 de 226 personas han respondido

Teléfono 143 res 63.3%

Correo electrónico 78 res 34.5%

Whatsapp 75 res 33.2%

Videollamada 46 res 20.4%

Facebook / Redes Sociales 22 res 9.7%

App 13 res 5.8%

Plataforma propia 5 res 2.2%

Other 63 res 27.9%

¿En qué medida necesita citas presenciales ocasionales con el equipo de Medicina Física y Rehabilitación para seguir la terapia en casa?

226 de 226 personas han respondido

3.8 Puntuación media

1	2	3	4	5	6
14.6%	10.2%	17.7%	19.9%	15.5%	22.1%
33 res	23 res	40 res	45 res	35 res	50 res

Poco — Mucho

¿En qué medida le sería suficiente la cita telefónica con el equipo de Medicina Física y Rehabilitación para seguir la terapia en casa?

226 de 226 personas han respondido

3.5 Puntuación media

12.8%	12.8%	24.3%	24.3%	14.2%	11.5%
29 res	29 res	55 res	55 res	32 res	26 res

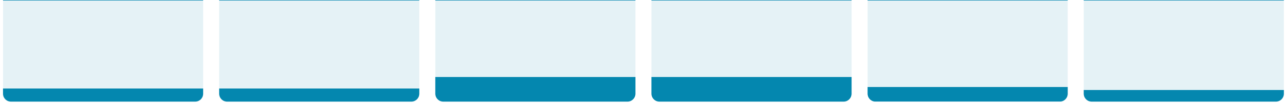

1 2 3 4 5 6

Poco Mucho

¿En qué medida le sería suficiente la videollamada con el equipo de Medicina Física y Rehabilitación para seguir la terapia en casa?

226 de 226 personas han respondido

3.7 Puntuación media

1	2	3	4	5	6
11.5%	9.7%	24.3%	21.2%	18.6%	14.6%
26 res	22 res	55 res	48 res	42 res	33 res

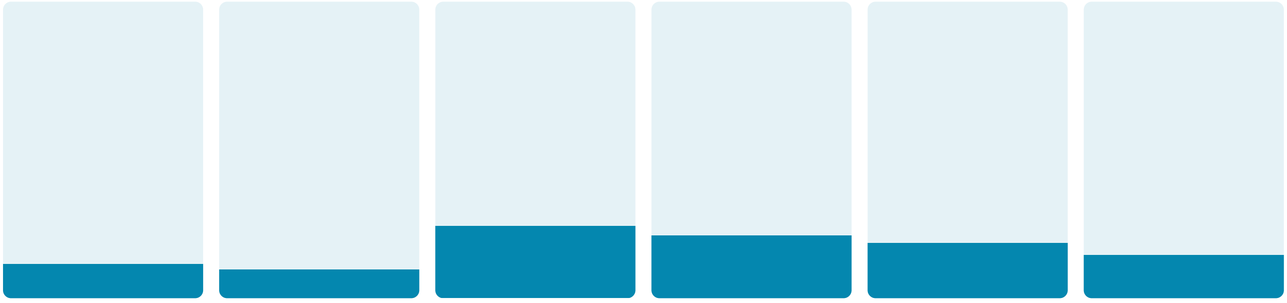

1 2 3 4 5 6

Poco Mucho

¿En qué medida un programa mixto de tratamiento presencial y no-presencial cubriría sus necesidades?

226 de 226 personas han respondido

4.4 Puntuación media

1	2	3	4	5	6
8.4%	4.4%	9.7%	21.2%	25.7%	30.5%
19 res	10 res	22 res	48 res	58 res	69 res

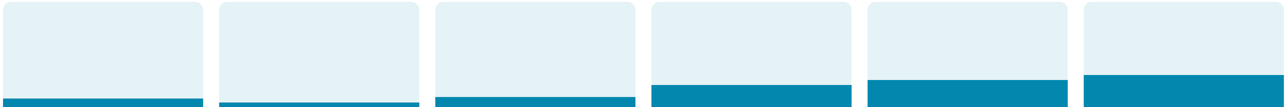

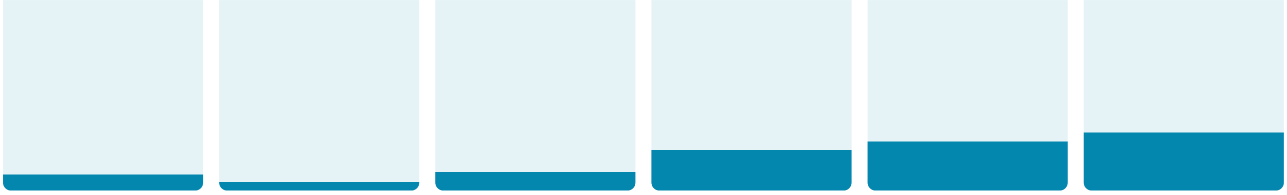

1 2 3 4 5 6

Poco Mucho

¿En qué medida le gustaría compartir sus experiencias con otros pacientes de forma virtual?

226 de 226 personas han respondido

3.5 Puntuación media

1	2	3	4	5	6
20.4%	11.5%	14.2%	21.2%	15.5%	17.3%
46 res	26 res	32 res	48 res	35 res	39 res

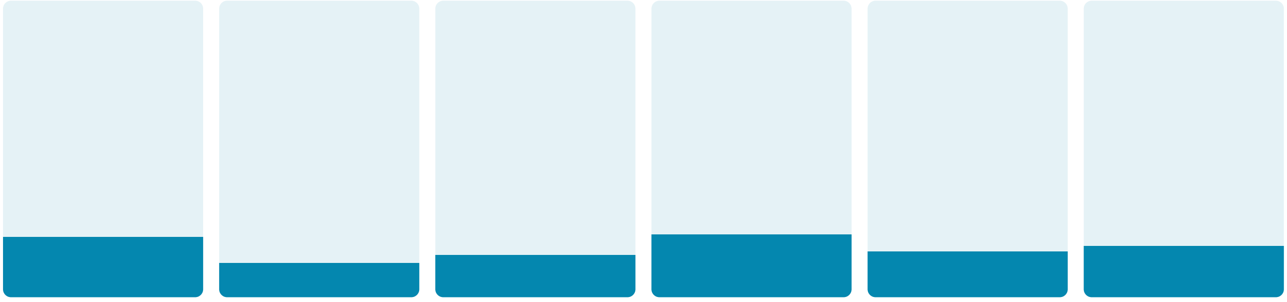

1 2 3 4 5 6

Poco Mucho

¿En qué medida considera que le motivaría hacer terapia de forma virtual con otros pacientes?

226 de 226 personas han respondido

3.4 Puntuación media

1	2	3	4	5	6
24.3%	11.5%	14.6%	18.1%	16.8%	14.6%
55 res	26 res	33 res	41 res	38 res	33 res

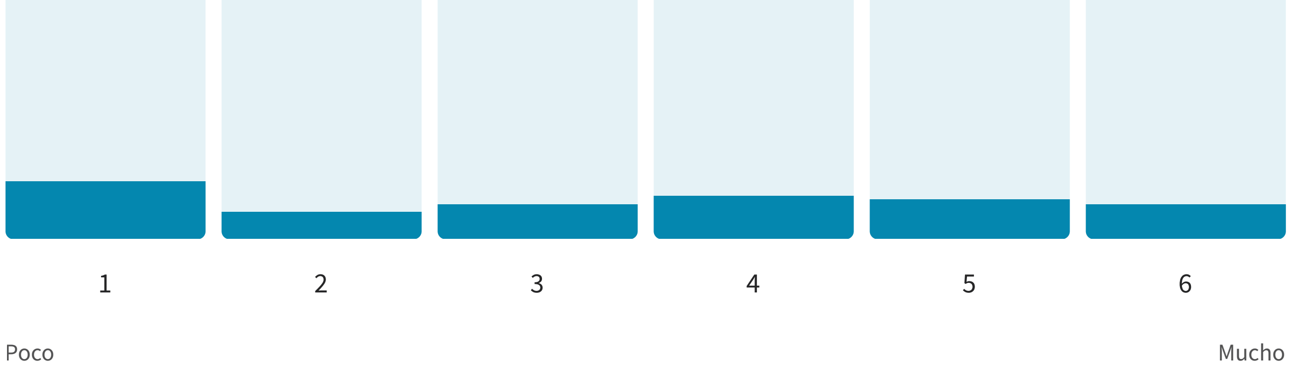

¿En qué medida considera útil poder solicitar consultas no presenciales?

226 de 226 personas han respondido

3.7 Puntuación media

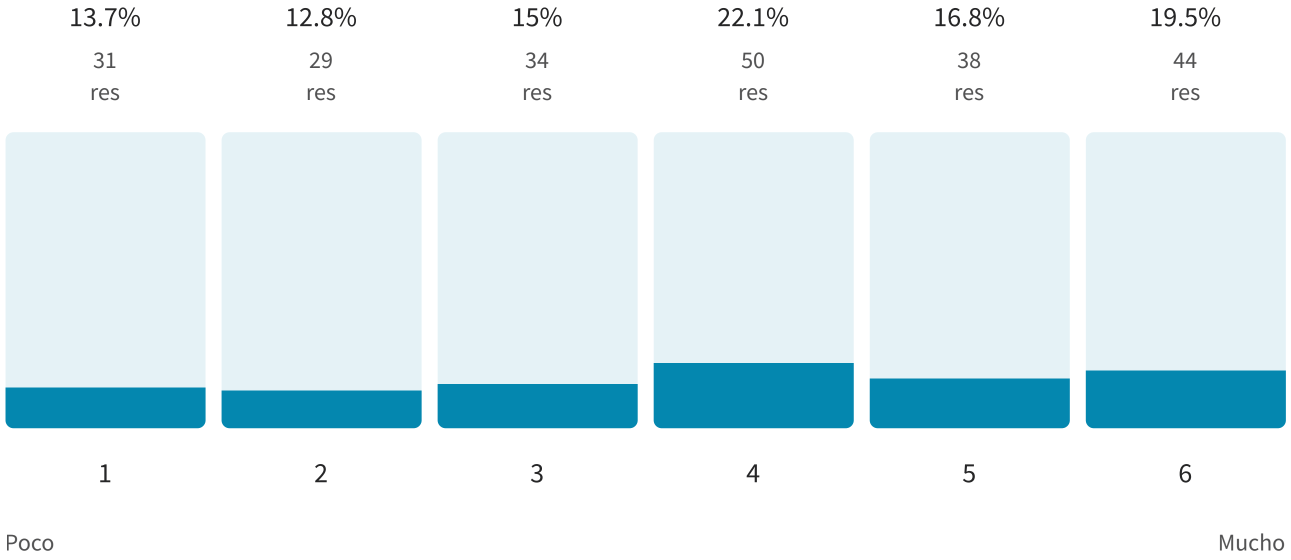

¿En qué medida considera que la visita no presencial sustituye a alguna de las visitas convencionales presenciales?

226 de 226 personas han respondido

3.0 Puntuación media

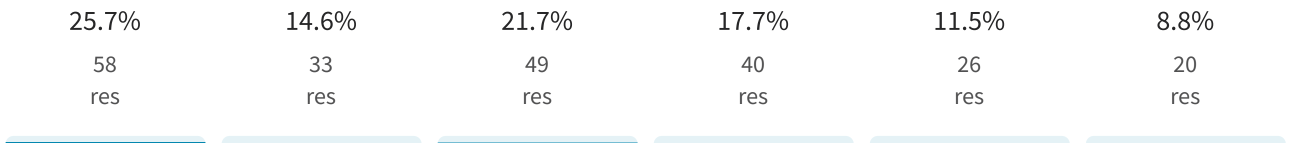

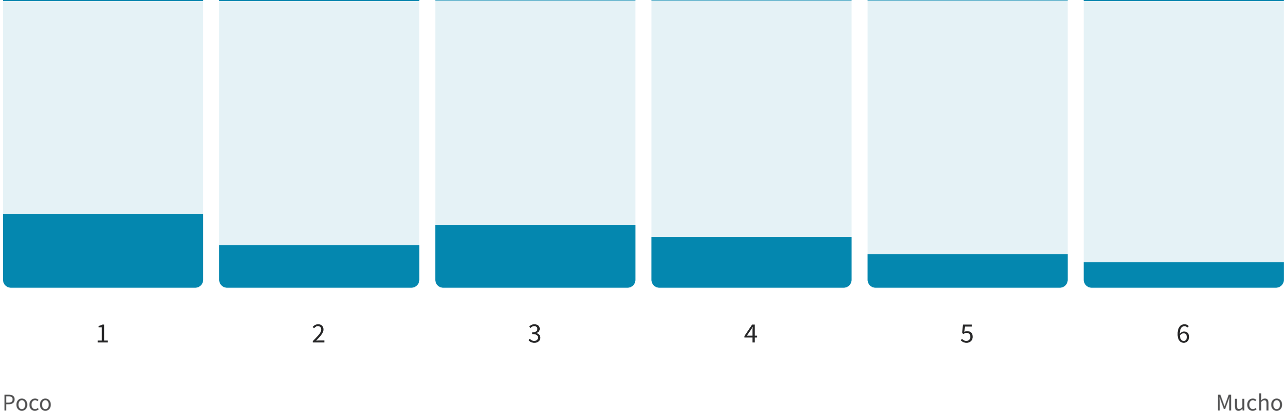

¿En qué medida le ayudaría disponer de unas instrucciones previas antes de la consulta no presencial?

226 de 226 personas han respondido

4.0 Puntuación media

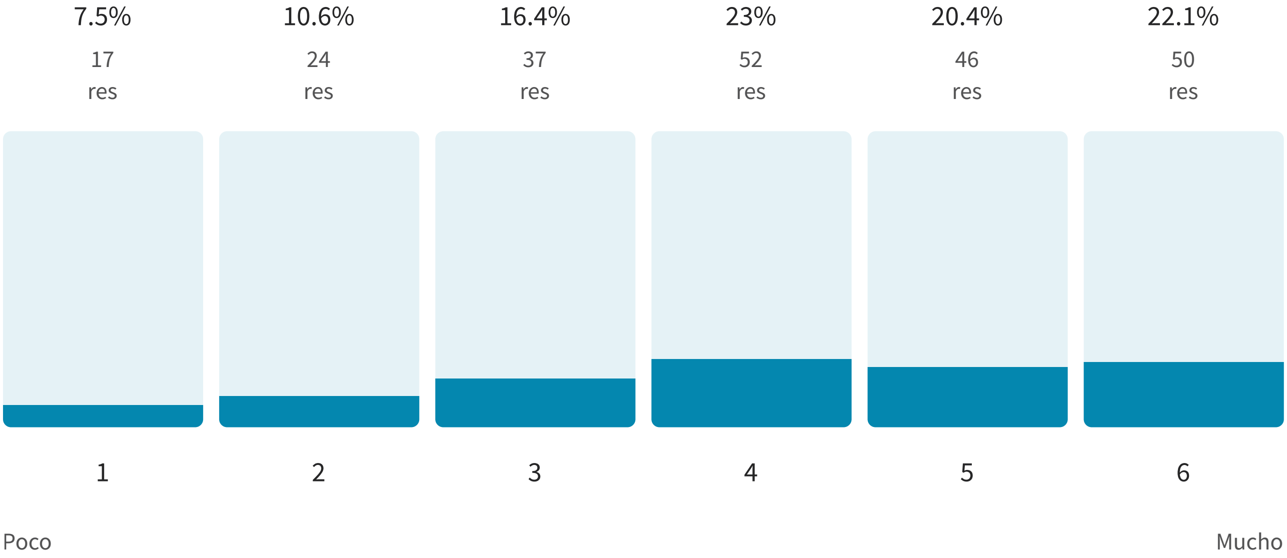

¿En qué medida resuelve sus dudas/necesidades con la consulta no presencial?

226 de 226 personas han respondido

3.4 Puntuación media

15% 11.9% 23.9% 21.2% 18.6% 9.3%

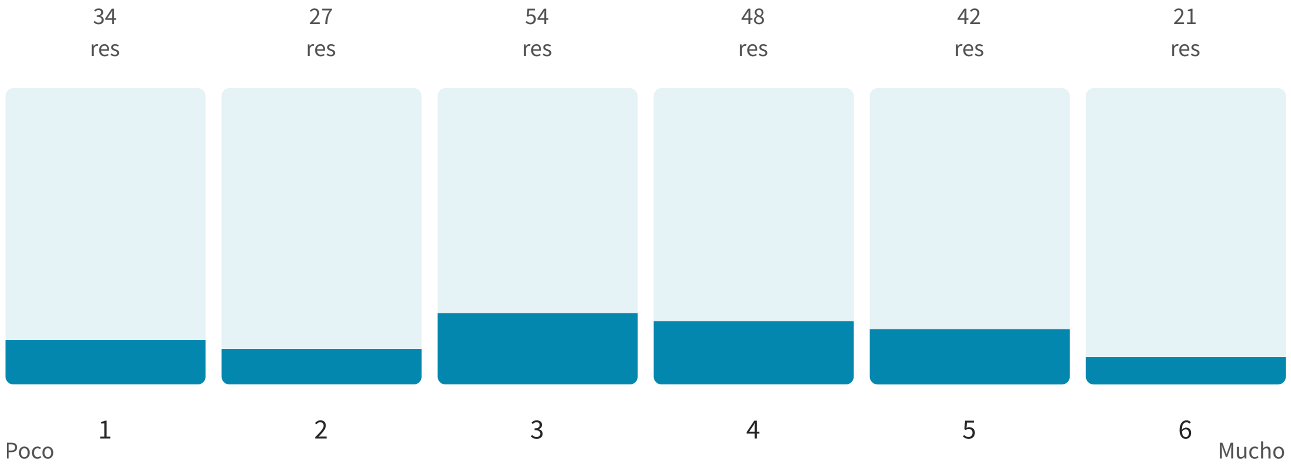

¿Consensua con su médico rehabilitador objetivos a alcanzar?

164 de 226 personas han respondido

4.2 Puntuación media

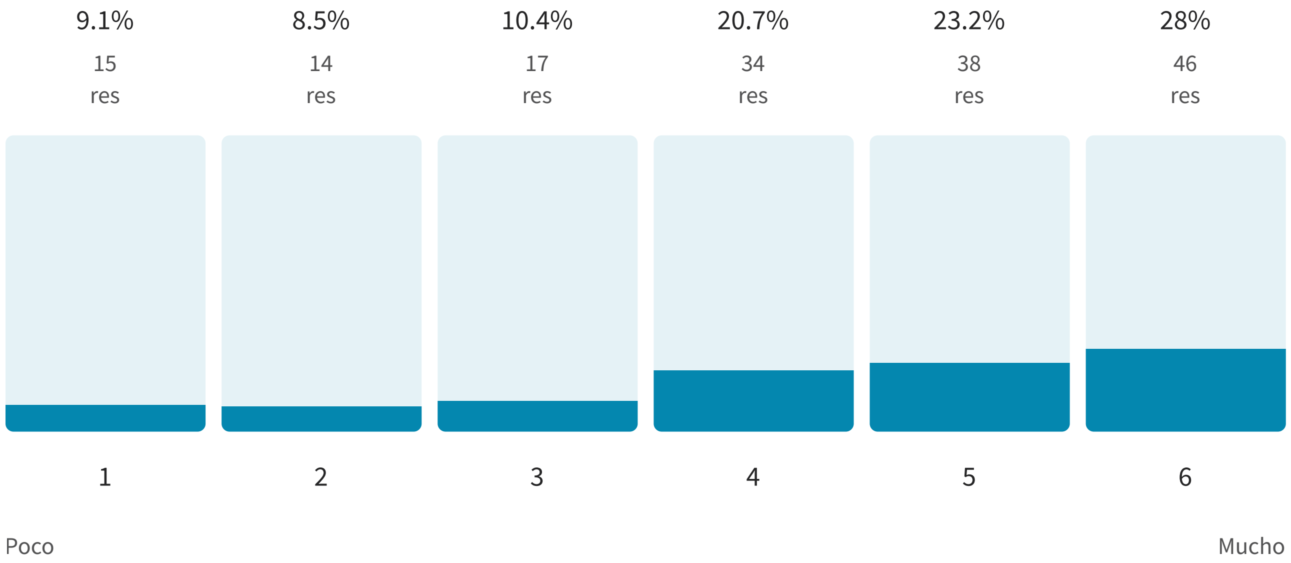

¿En qué medida considera que su patología afecta a su vida social?

226 de 226 personas han respondido

4.6 Puntuación media

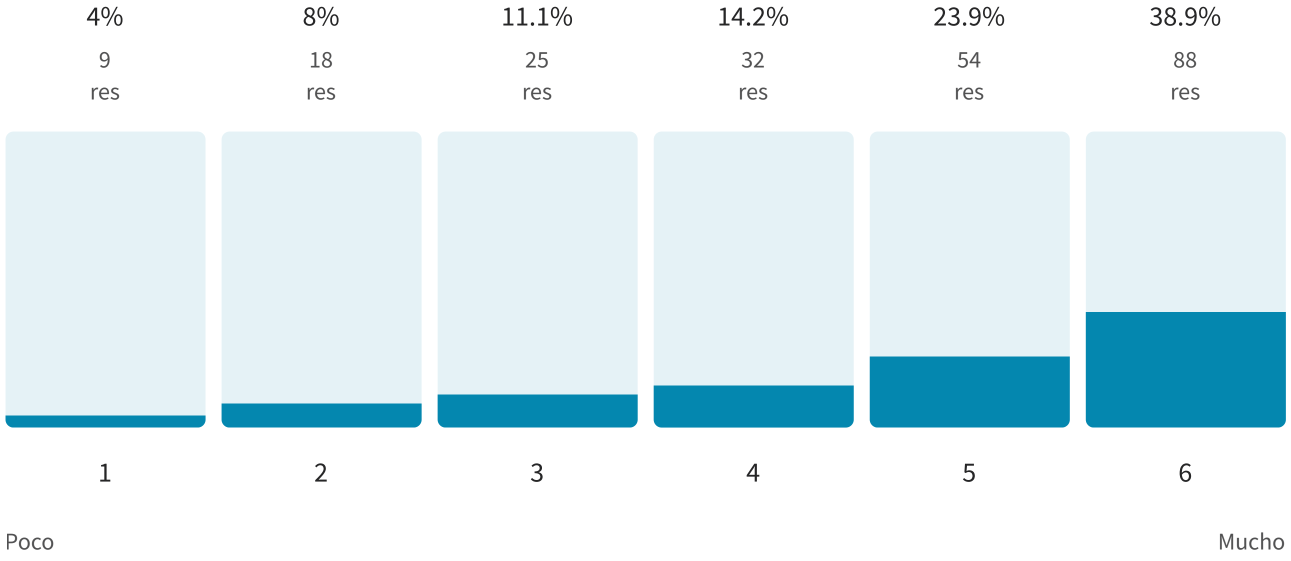

¿En qué medida considera que su patología afecta a su calidad de vida?

226 de 226 personas han respondido

4.7 Puntuación media

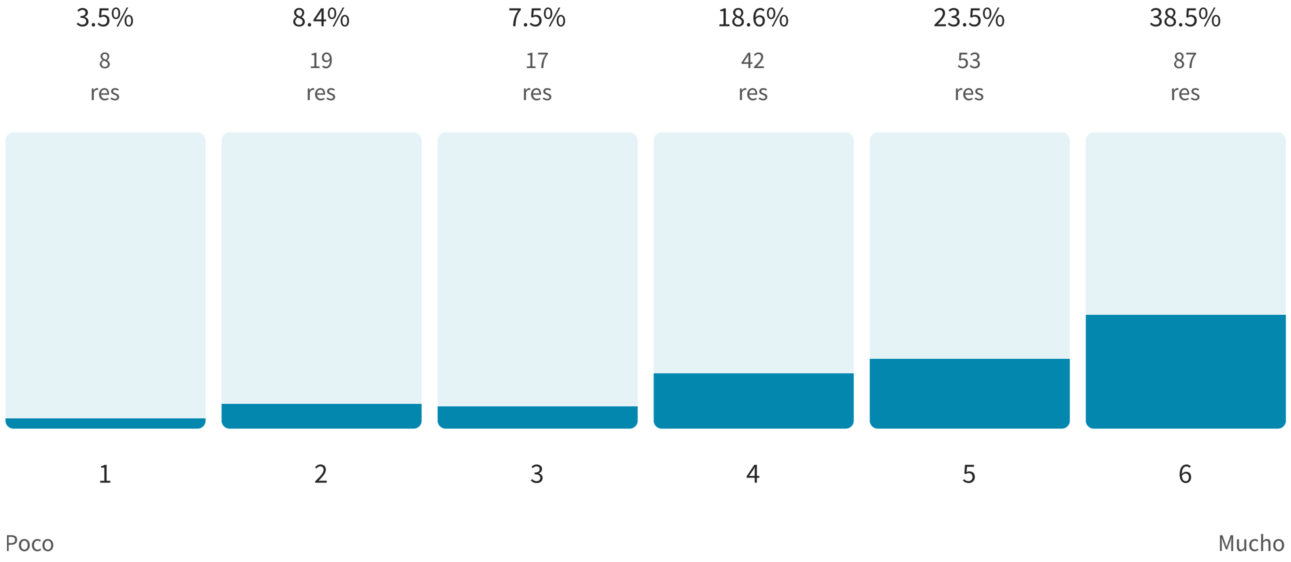

¿En qué medida está conforme con el tiempo de atención en consulta de rehabilitación?

178 de 226 personas han respondido

4.6 Puntuación media

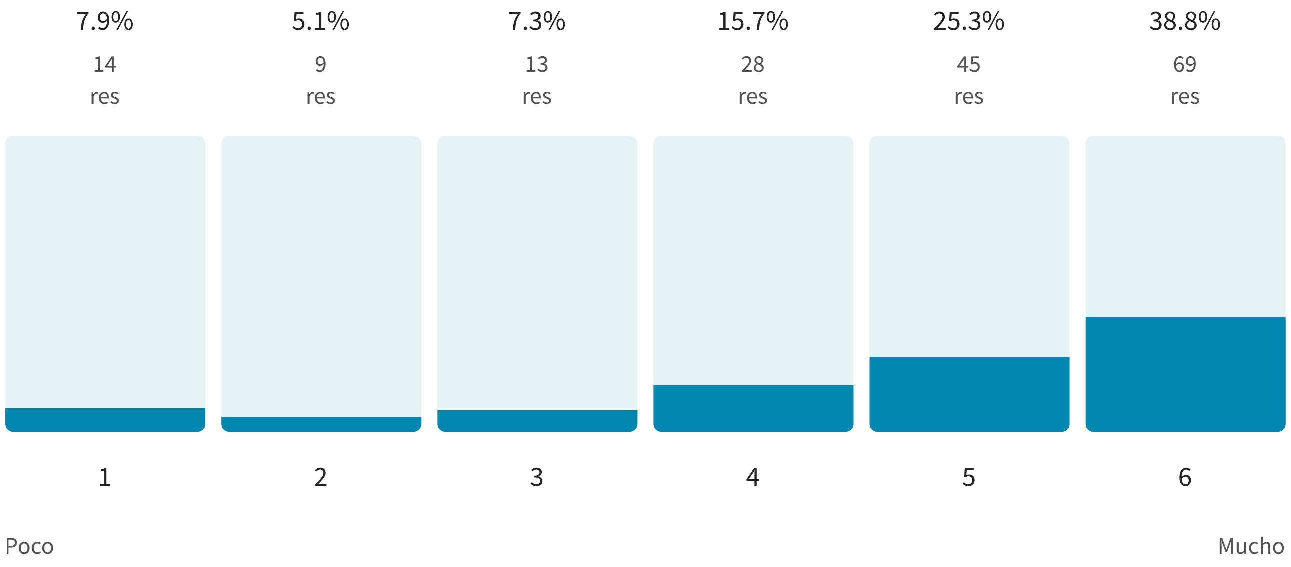

¿En qué medida cree que las revisiones en consulta con el médico rehabilitador le ayudan a continuar realizando los ejercicios en domicilio?

226 de 226 personas han respondido

4.6 Puntuación media

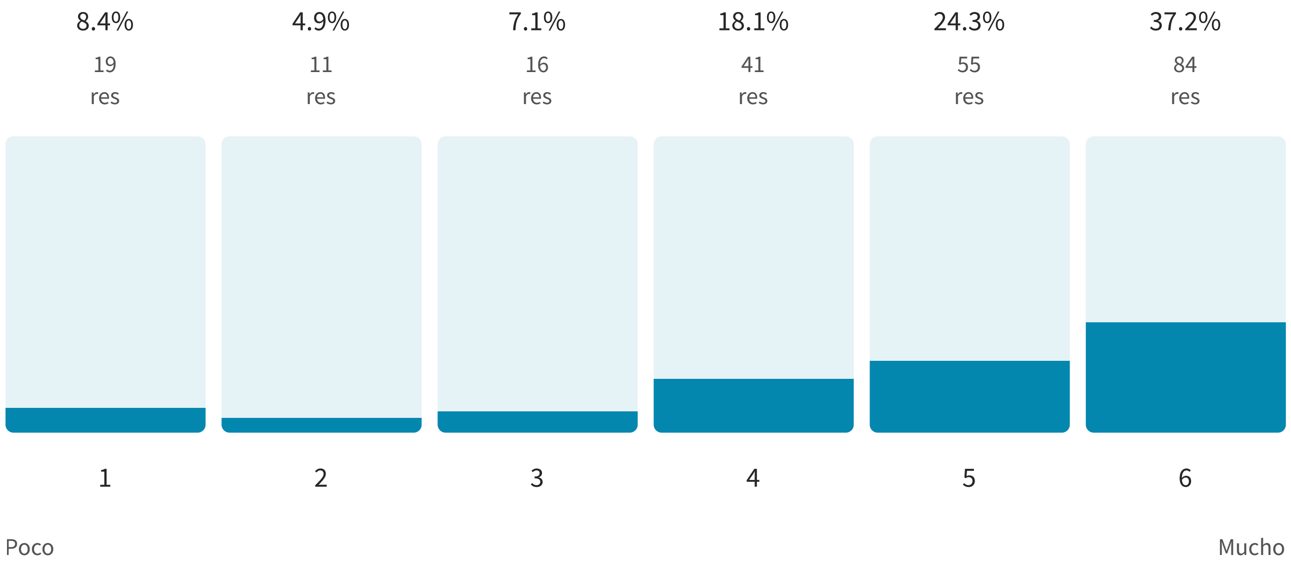

¿En qué medida le gustaría que hubiera algún tipo de aplicación en la que se volcara su entrenamiento domiciliario y su equipo de rehabilitación le realizara un seguimiento?

226 de 226 personas han respondido

4.7 Puntuación media

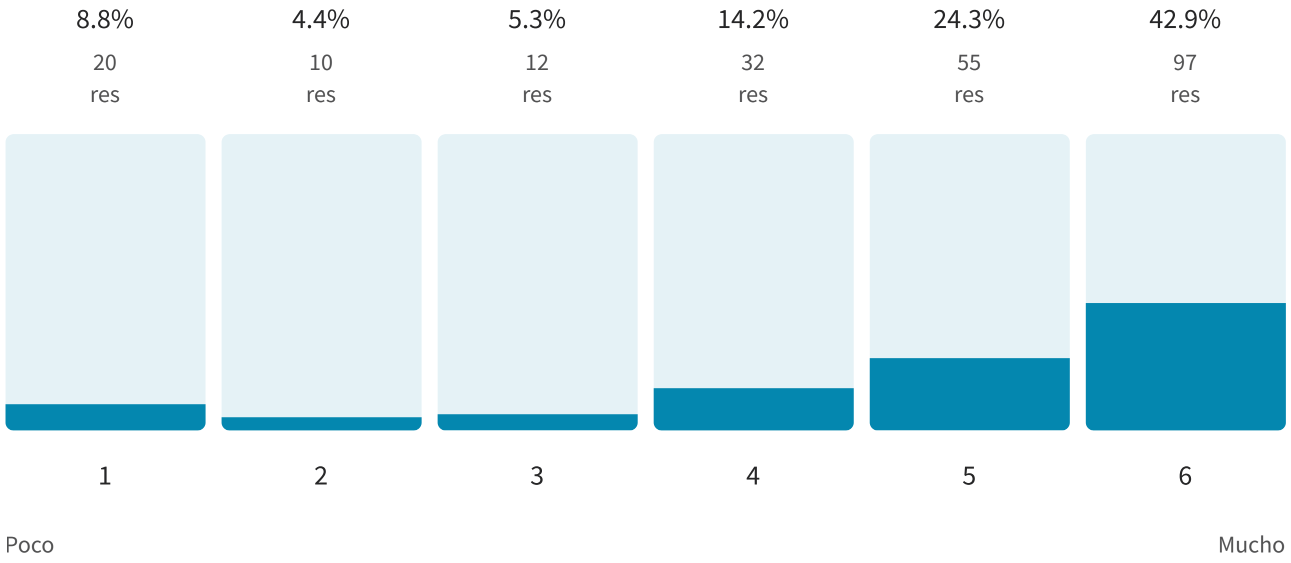

¿Considera que el médico rehabilitador que le ha atendido ha tenido en consideración la limitación social y la repercusión psicológica producida por la incontinencia?

174 de 226 personas han respondido

4.8 Puntuación media

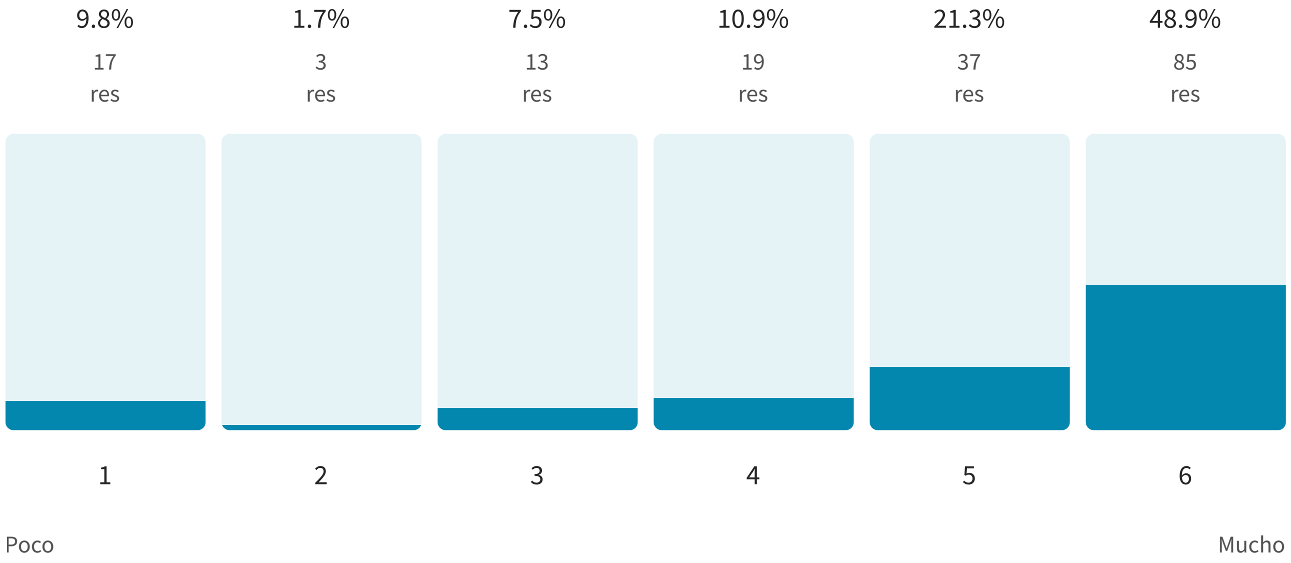

¿En qué grado considera usted que puede necesitar tratamiento de Psicología/Psiquiatría para complementar su tratamiento de la patología de suelo pélvico?

226 de 226 personas han respondido

3.1 Puntuación media

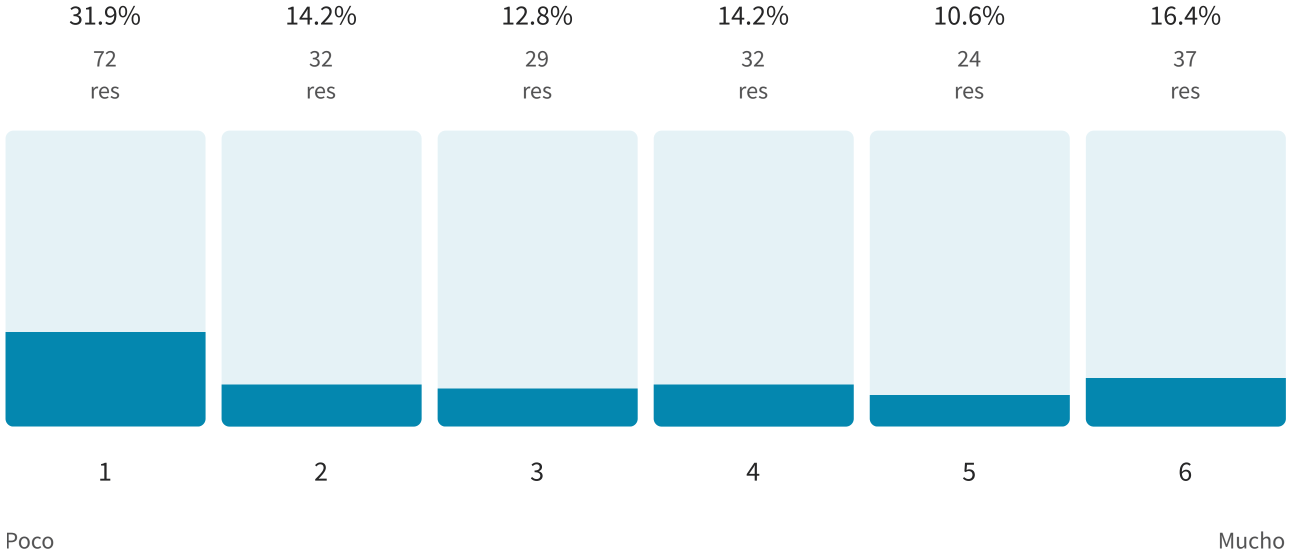

¿En su ámbito sanitario ha tenido facilidad para acceder a una unidad de rehabilitación de suelo pélvico?

226 de 226 personas han respondido

3.6 Puntuación media

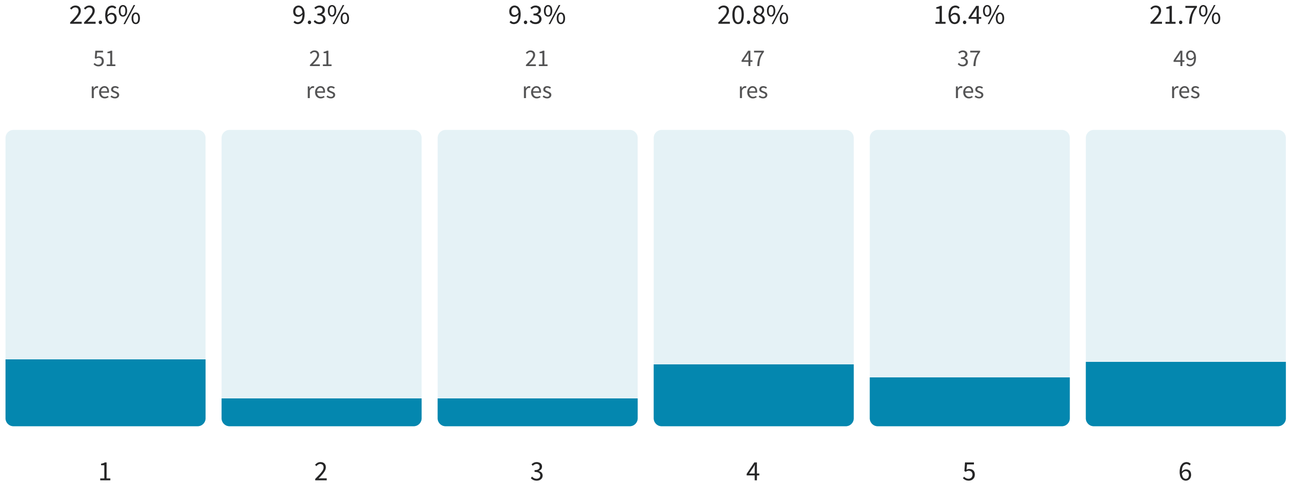

Poco Mucho

¿Considera que debería haber sido derivado antes a la unidad de rehabilitación de suelo pélvico?

226 de 226 personas han respondido

4.5 Puntuación media

1	2	3	4	5	6
12.8%	6.6%	7.5%	9.7%	16.8%	46.5%
29 res	15 res	17 res	22 res	38 res	105 res

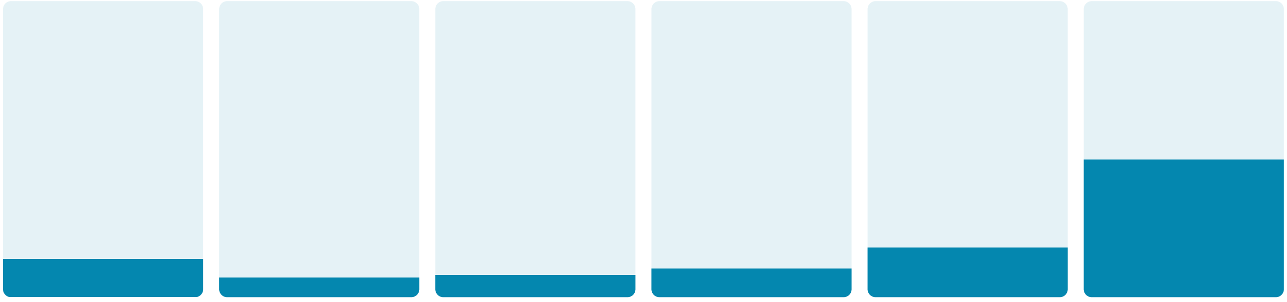

Poco Mucho

Si ha tenido usted que realizar un diario miccional/defecatorio ¿Tiene dificultades para cumplimentarlo?

133 de 226 personas han respondido

2.6 Puntuación media

1	2	3	4	5	6
42.1%	12.8%	12%	15%	8.3%	9.8%
56 res	17 res	16 res	20 res	11 res	13 res

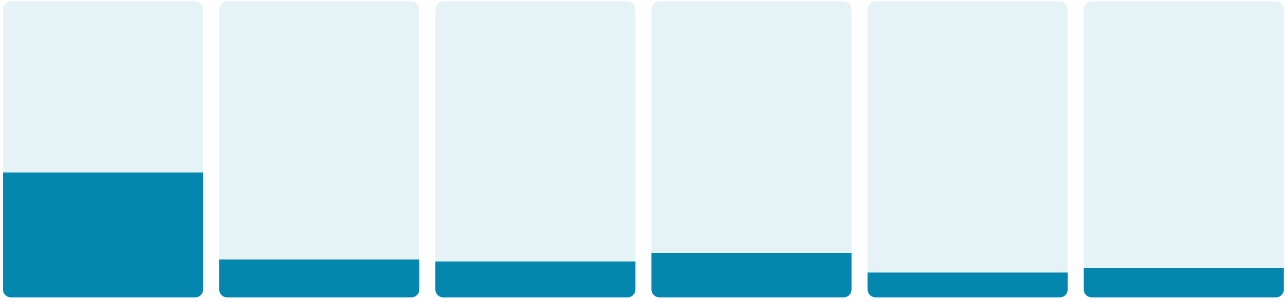

1 2 3 4 5 6

Poco Mucho

Si ha tenido usted que realizar un diario miccional/defecatorio ¿Cree que le ha sido de utilidad en la reducción/mejoría de su incontinencia?

123 de 226 personas han respondido

2.9 Puntuación media

29.3%	14.6%	19.5%	16.3%	10.6%	9.8%
36 res	18 res	24 res	20 res	13 res	12 res

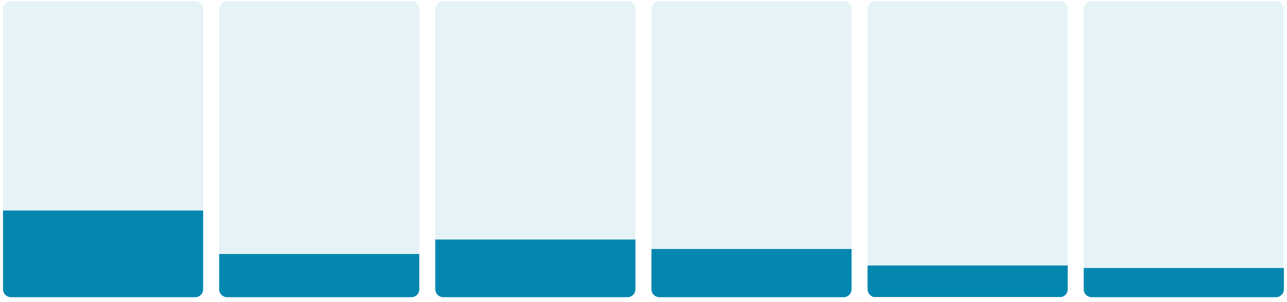

1 2 3 4 5 6

Poco Mucho

Si tiene diagnosticada una vejiga hiperactiva/ incontinencia por urgencia/ nocturia o incremento de la frecuencia miccional y le han puesto un tratamiento con fármacos ¿En qué grado cumple con el tratamiento?

66 de 226 personas han respondido

3.9 Puntuación media

25.8%	7.6%	6.1%	13.6%	9.1%	37.9%
17 res	5 res	4 res	9 res	6 res	25 res

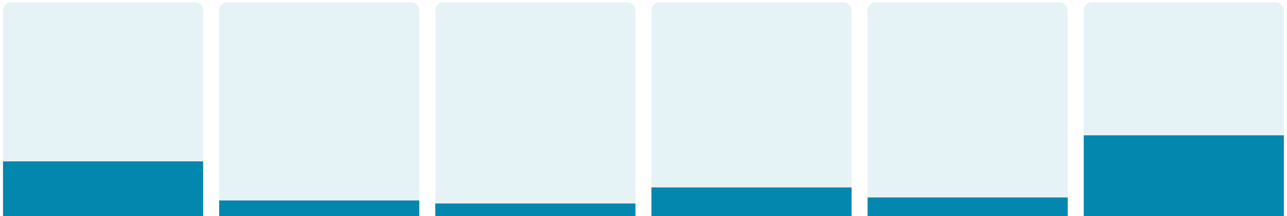

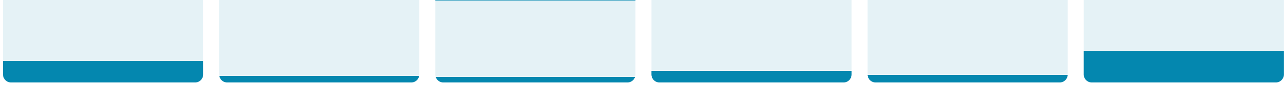

1 2 3 4 5 6

Nunca — Siempre

¿En qué medida cree que las instrucciones escritas entregadas en la consulta de rehabilitación pueden ayudarle a realizar los ejercicios en su domicilio?

226 de 226 personas han respondido

4.2 Puntuación media

1	2	3	4	5	6
10.6%	5.8%	11.5%	19.9%	25.7%	26.5%
24 res	13 res	26 res	45 res	58 res	60 res

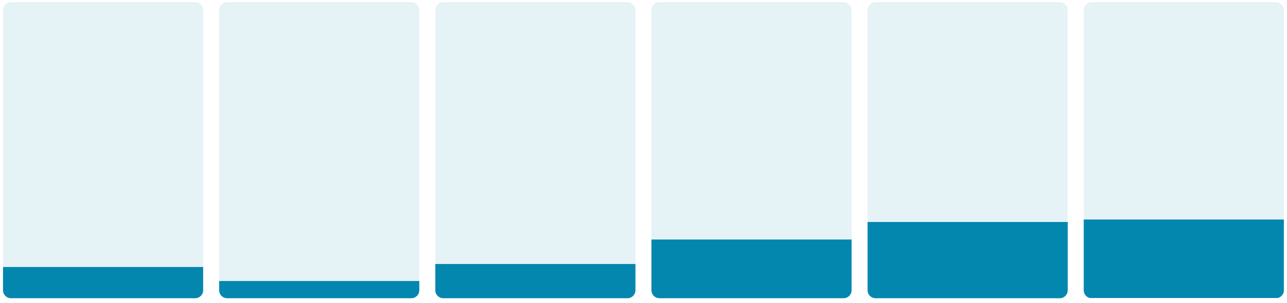

1 2 3 4 5 6

Poco — Mucho

¿En qué medida cree que los ejercicios en video pueden ayudarle a realizar los ejercicios en su domicilio?

226 de 226 personas han respondido

4.6 Puntuación media

1	2	3	4	5	6
6.2%	4.9%	6.2%	20.4%	26.1%	36.3%
14 res	11 res	14 res	46 res	59 res	82 res

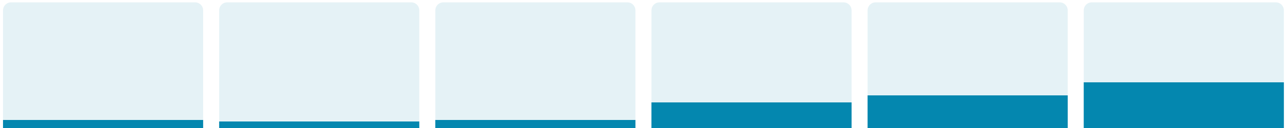

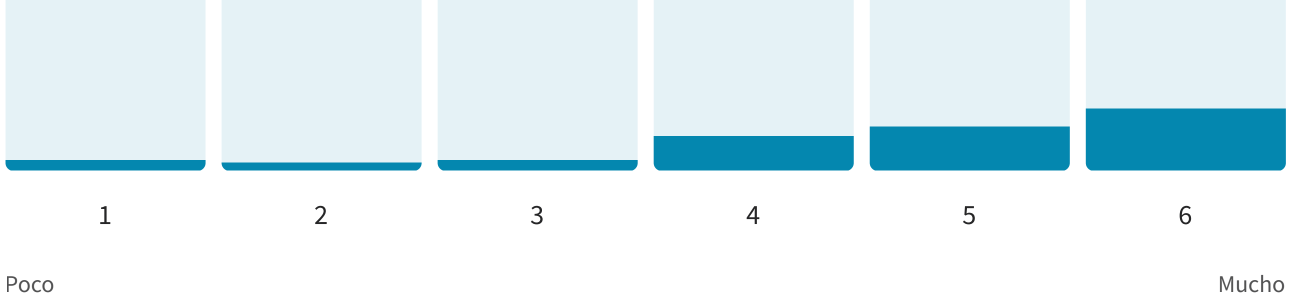

¿En qué medida le gustaría asistir a un programa de charlas y tratamiento grupal?

226 de 226 personas han respondido

3.7 Puntuación media

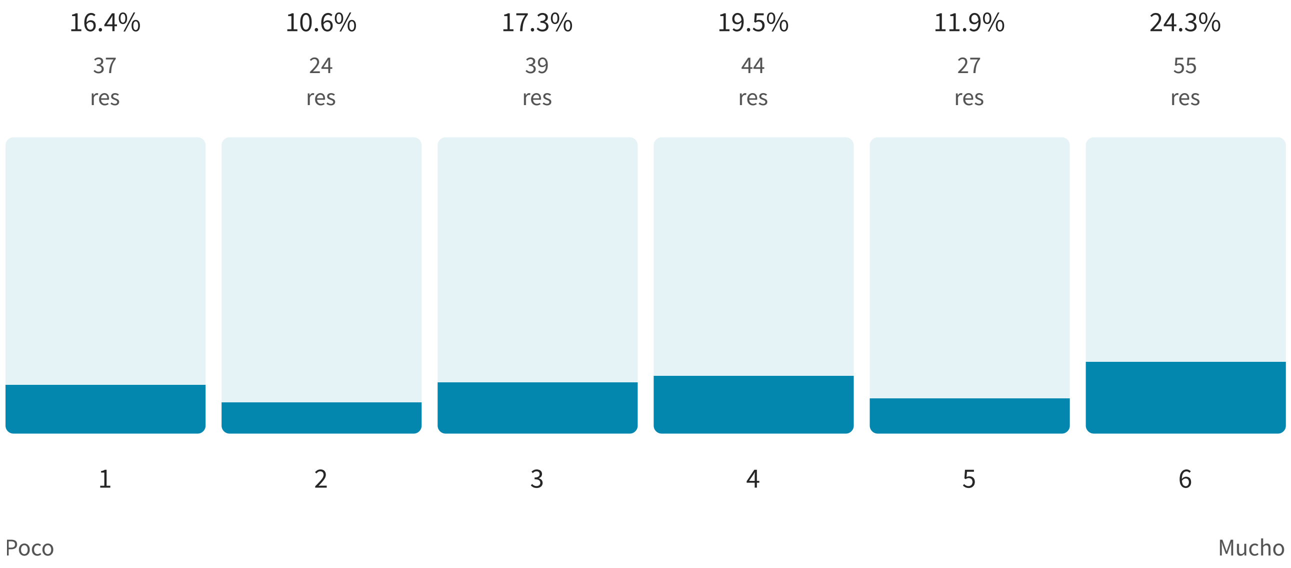

Si ya ha realizado tratamiento grupal ¿le ha sido útil?

60 de 226 personas han respondido

3.3 Puntuación media

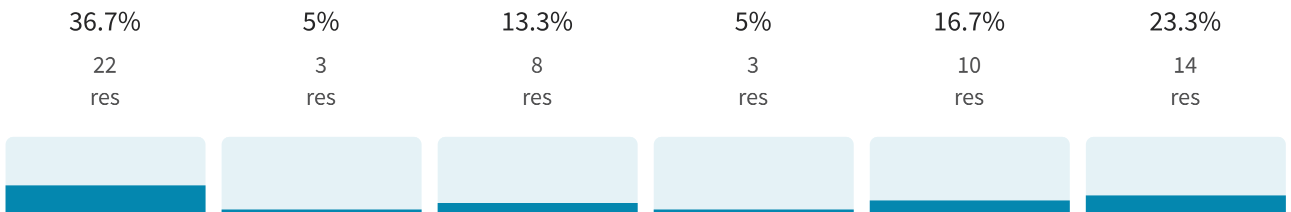

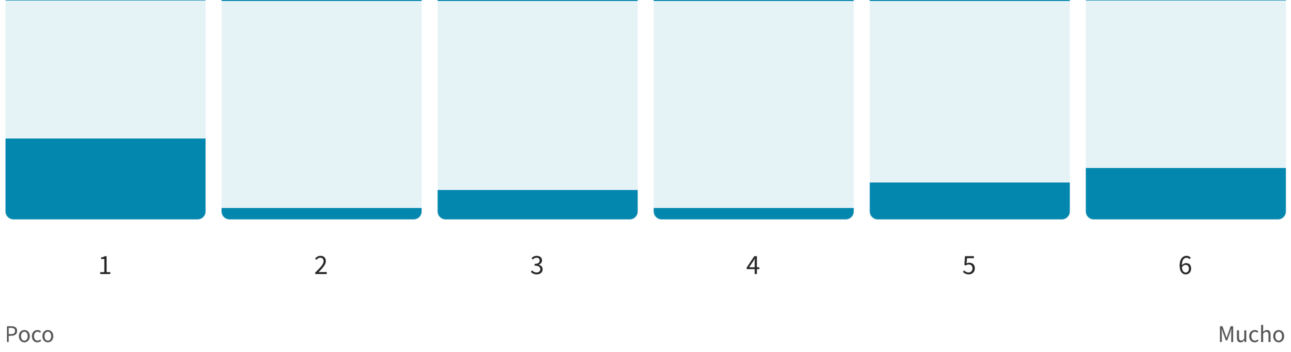

¿Ha sido informado por su médico rehabilitador o fisioterapeuta sobre la posibilidad de complementar los ejercicios de SP en su domicilio con otros dispositivos?

226 de 226 personas han respondido

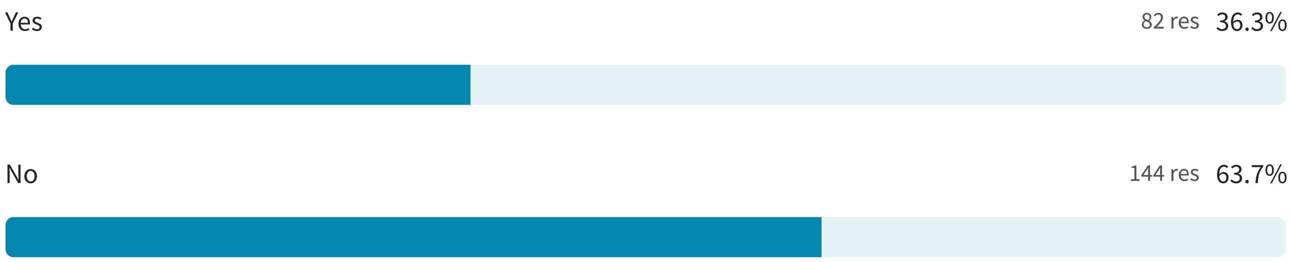

¿En qué medida el uso de otros dispositivos pueden ayudar que usted realice los ejercicios en su domicilio?

78 de 226 personas han respondido

4.6 Puntuación media

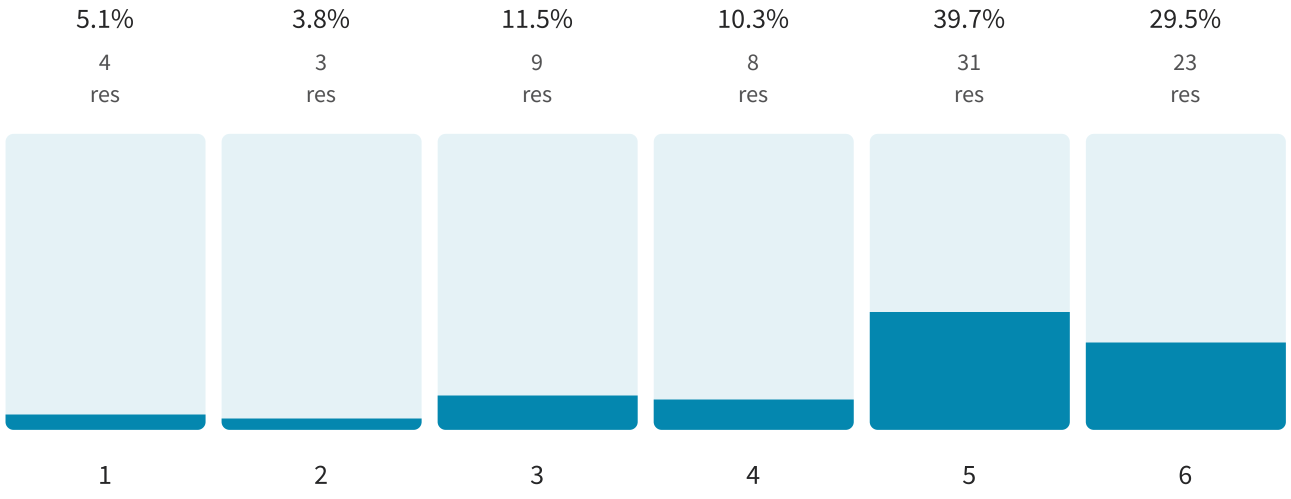

Poco Mucho

¿En qué medida cree que el uso de aplicaciones móviles, recomendadas por un profesional, pueden ayudar que usted realice los ejercicios en su domicilio?

226 de 226 personas han respondido

4.3 Puntuación media

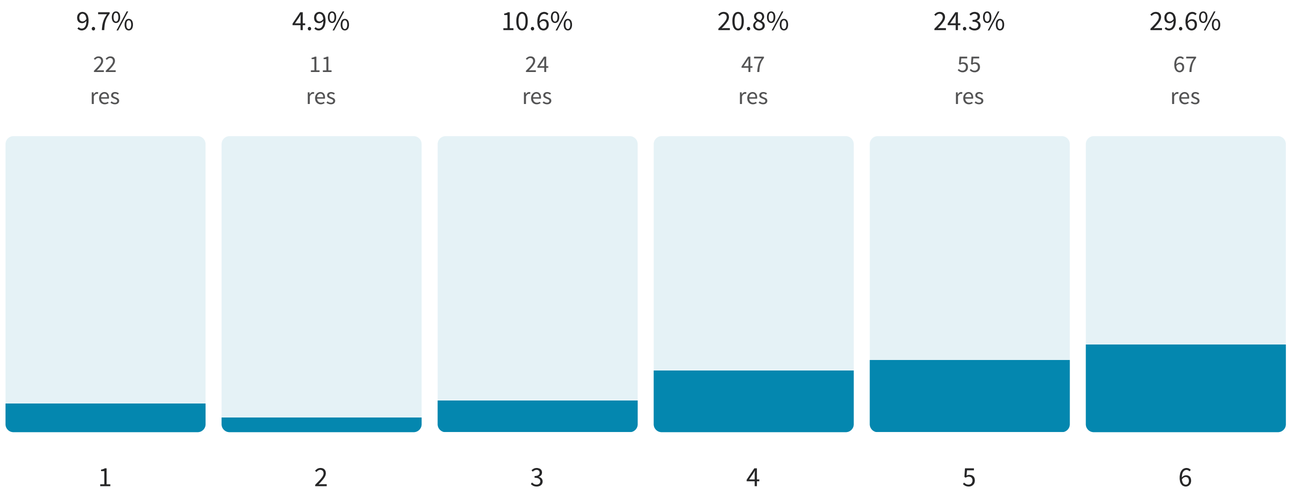

Poco Mucho
